# An unusual titanosaur axis from the Upper Cretaceous of Brazil and its significance for sauropod anatomy and systematics

**DOI:** 10.1002/ar.70118

**Published:** 2026-01-06

**Authors:** Bruno A. Navarro, Jeffrey A. Wilson Mantilla, Jhonata H. A. Martins, Fabiano V. Iori, Leonardo S. Paschoa, Alberto B. Carvalho, Joseli M. Piranha, Hussam Zaher

**Affiliations:** ^1^ Graduate Program in Zoology Instituto de Biociências, Universidade de São Paulo São Paulo São Paulo Brazil; ^2^ Laboratório de Herpetologia e Paleontologia Museu de Zoologia, Universidade de São Paulo São Paulo Sao Paulo Brazil; ^3^ Museum of Paleontology, University of Michigan Ann Arbor Michigan USA; ^4^ Department of Earth and Environmental Sciences University of Michigan Ann Arbor Michigan USA; ^5^ Graduate Program in Animal Biodiversity Universidade Federal de Santa Maria Santa Maria Rio Grande do Sul Brazil; ^6^ Centro de Apoio à Pesquisa Paleontológica da Quarta Colônia (CAPPA) Universidade Federal de Santa Maria São João do Polêsine Rio Grande do Sul Brazil; ^7^ Museu de Paleontologia ‘Pedro Candolo’ Uchoa São Paulo Brazil; ^8^ Museu de Paleontologia ‘Professor Antonio Celso de Arruda Campos’ Monte Alto São Paulo Brazil; ^9^ Instituto de Biociências, Letras e Ciências Exatas Universidade Estadual Paulista ‘Júlio de Mesquita Filho’ São José do Rio Preto São Paulo Brazil

**Keywords:** axial pneumaticity, Bauru Group, cervical series, comparative anatomy, São José do Rio Preto Formation, Titanosauria

## Abstract

The Upper Cretaceous São José do Rio Preto Formation (Bauru Group, southeastern Brazil) has yielded a fragmentary but taxonomically diverse record of titanosaur sauropods, although elements from cervical series remain scarce. Here, we describe a nearly complete sauropod axis from the Vila Ventura Paleontological Area, representing an uncommon occurrence within this unit and the Bauru Group as a whole. The Vila Ventura axis exhibits a broad axial intercentrum with paired ventral fossae, an elongated, goblet‐shaped axial pleurocentrum that is longer than high, and a tall neural arch. Microcomputed tomography imaging reveals extensive camellate‐type internal pneumaticity, including radial and circumferential camellae in the pleurocentrum with incipient development of internal bony plates—traits that are previously found in highly nested titanosaurs, such as saltasaurines. Comparative osteological analysis, however, indicates closer affinities with colossosaurians (e.g., *Bonitasaura*, *Futalognkosaurus*, *Pitekunsaurus*), particularly in centrum proportions and laminar configuration, including an enlarged odontoid, a V‐shaped ventral median keel with ventrolateral excavations in the pleurocentrum, a stout prespinal lamina terminating in an anterior spinal projection, and well‐developed pneumatopores in the spinodiapophyseal fossae. Additionally, the specimen exhibits unique features, such as a stranded and laterally expanded spinoprezygapophyseal lamina and a transversely broad anterior spinal projection. An evaluation of axis morphology highlights the underrepresentation of phylogenetic characters from this element in current datasets, despite its potential diagnostic value for assessing lower‐level relationships within Titanosauria. This discovery increases the diversity of Brazilian titanosaur records and emphasizes the importance of axial elements in understanding sauropod functional morphology and phylogeny.

## 1 INTRODUCTION

Titanosauria accounts for about one‐third of the total diversity of sauropod dinosaurs (Curry Rogers, 2005; Wilson, 2006a). Titanosaurs were distributed globally and represent the predominant large herbivores in the Upper Cretaceous faunas of Gondwanan landmasses—particularly in South America, where over 60 currently valid species have been identified (Carballido et al., 2022; Cerda et al., 2012). Despite its richness and spatiotemporal range, the titanosaur fossil record is characterized by uneven sampling. Complete titanosaur skeletons are exceptionally rare, which constrains our understanding of their anatomy and phylogenetic relationships (Cashmore et al., 2020; Mannion & Upchurch, 2010).

This discrepancy is related to intrinsic factors that influence preservation, including the number, size, and structural integrity of skeletal elements, as well as extrinsic factors, such as the substantial sedimentary supply required to bury large‐bodied individuals (González Riga et al., 2022; Mannion et al., 2011). Consequently, compact axial elements (e.g., caudal vertebrae) and large limb bones (e.g., humeri and femora) are the most common elements found at titanosaur sites. By contrast, smaller and delicate elements are comparably rare, especially those on extremities of the skeleton, such as the skull, anteriormost cervical vertebrae, distalmost caudal vertebrae, manus, and pes (González Riga, 2010). This differential pattern of preservation negatively impacts phylogenetic analyses due to the loss of character data, and the non‐overlap of anatomical data hinders direct comparisons among taxa, limiting character scoring and recovery of potential synapomorphies that could discriminate lower‐level relationships (Wilson et al., 2016).

Important anatomical modules of the titanosaur skeleton remain unknown or underrepresented in several specimens. For instance, the rarity of complete cervical series is a predominant factor in Sauropoda (Taylor, 2022), where most complete known cervical series often exhibit taphonomic distortion or lack elements anatomically informative (e.g., atlantoaxial elements). The scarcity of key anatomical elements contrasts with the abundance of titanosaur fossils. In Brazil, for example, the record of titanosaurs is numerous and spans much of the Cretaceous interval. Lower to “middle” Cretaceous occurrences are predominantly in northeast Brazil (e.g., Carvalho et al., 2017; Ghilardi et al., 2016; Lindoso et al., 2013; Medeiros et al., 2014; Pereira et al., 2018, 2024; Zaher et al., 2011), whereas Upper Cretaceous occurrences are mainly from the mid‐west and southeast regions of the country (e.g., Bandeira et al., 2019; Gil et al., 2020). Most titanosaur occurrences come from deposits of the Bauru Group (Paraná Basin), from which 11 species have been described (Santucci & Filippi, 2022). Although most of the titanosaur species from the Bauru Group preserve cervical vertebrae, these are predominantly post‐axial elements, in particular middle‐to‐posterior cervical vertebrae (Bandeira et al., 2016; Campos et al., 2005; Kellner et al., 2006; Kellner & de Azevedo, 1999; Machado et al., 2013; Navarro et al., 2022; Salgado & Carvalho, 2008; Santucci & Arruda‐Campos, 2011; Silva Junior et al., 2019). Apart from a few unnamed specimens (e.g., Martinelli et al., 2015; Powell, 2003), none of the nominal taxa from the Bauru Group preserved atlantoaxial elements.

In this contribution, we describe a nearly complete sauropod axis collected from the São José do Rio Preto Formation, at the Vila Ventura Paleontological Area (Bauru Group, northeastern São Paulo State, Brazil; Figure 1). We refer the specimen to Titanosauria based on its high degree of internal pneumaticity and the age of the deposits (Santonian–Campanian), from which only titanosaurs have been recovered in the Upper Cretaceous of South America (Carballido et al., 2022). We provide osteological comparisons to a broad range of sauropod axes to assess the main anatomical aspects of the Vila Ventura specimen. Additionally, we explore the phylogenetic significance of the axial morphology for titanosaur systematics, evaluating its potential utility as character data in cladistic analyses and their possible implications on neck structure within Sauropoda.

**FIGURE 1:**
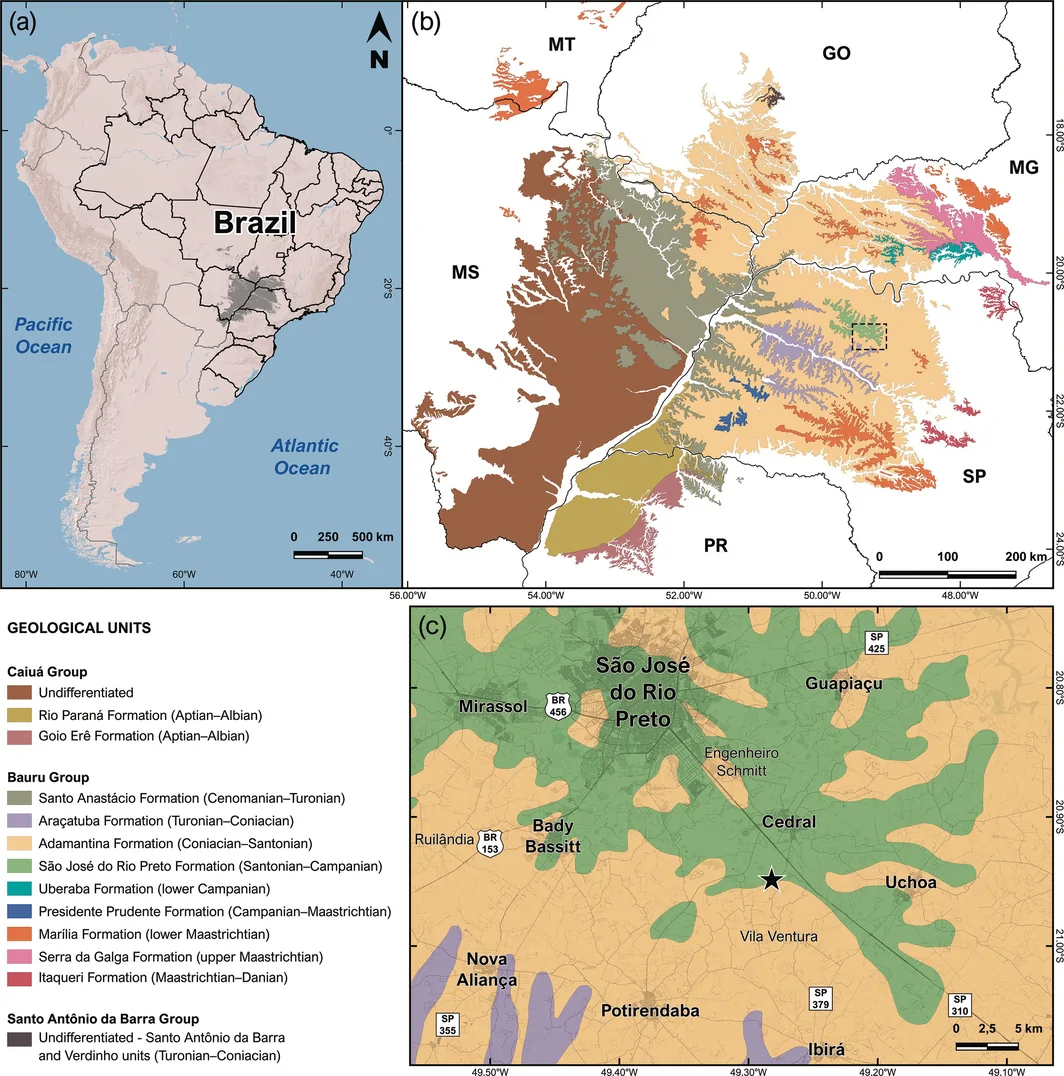
Geographic and stratigraphic provenance of the Vila Ventura axis (MPPC/GPRD 25‐013). Extent of the Bauru Supersequence across South America (a) and central Brazil (b). The dashed rectangle in (b) indicates the enlarged area (c), where the black star corresponds to the fossil site. Abbreviations: GO, Goiás State; MG, Minas Gerais State; MS, Mato Grosso do Sul State; MT, Mato Grosso State; PR, Paraná State; SP, São Paulo State.

## 2 GEOLOGICAL AND PALEONTOLOGICAL BACKGROUND

The Bauru Supersequence comprises a large continental inner sag covering approximately 380,000 km^2^ of central Brazil and part of southeastern Paraguay (Figure 1a). These deposits reach approximately 300 m of maximum thickness, preserving a predominantly sandy sedimentary sequence formed under semiarid to arid climatic conditions (Basilici & Sgarbi, 2012; Dias‐Brito et al., 2001). This sedimentary sequence is divided into two main geological units, the Caiuá and Bauru groups, which are separated from each other by erosive unconformities (Batezelli, 2015; de Paula e Silva et al., 2005, 2009).

The Caiuá Group comprises possibly Aptian‐to‐Albian‐aged eolian sandstones deposited under sand sea and wadi environments (Figure 1b) and is characterized by sparse fossil occurrences (e.g., De Souza et al., 2021; Kellner et al., 2019; Langer et al., 2019; Manzig et al., 2014; Pêgas, 2024; Simões et al., 2015). By contrast, the Bauru Group preserves one of the most prolific fossiliferous assemblages from South America (see Langer et al., 2022; Martinelli & Teixeira, 2015; Menegazzo et al., 2016 for an overview) and includes sandstones, siltstones, and mudstones deposited under palustrine, fluvial, and alluvial environments (Batezelli, 2010; Fernandes & Basilici, 2009). Age estimates range from the Cenomanian to the Maastrichtian, although some authors suggest it may extend into the early Danian (Fernandes & Magalhães Ribeiro, 2015; Menegazzo et al., 2016).

The Bauru Group crops out across nearly half of the territorial extent of São Paulo State (Figure 1b) and is represented by the Santo Anastácio, Birigui (only in subsurface), Araçatuba, Adamantina, São Carlos, São José do Rio Preto, Presidente Prudente, Marília, and Itaqueri formations (see Arai & Dias‐Brito, 2023; Navarro et al., 2025). The new specimen described here was recovered from deposits of the São José do Rio Preto Formation, in northeastern São Paulo State (Figure 1c). This unit comprises immature sandstones that are often conglomeratic, accumulating in fluvial bars and plains of wide and shallow channels of intertwined fluvial systems with considerable energy. The São José de Rio Preto Formation crops out predominantly in the region of the eponymous city (Fernandes & Coimbra, 2000), but it is also exposed in other areas (see Goldberg & Garcia, 2000). The conglomeratic facies preserve most of the fossil content from this unit, although some specimens are also recovered from fine‐grained facies associated with slower‐flow conditions. The fossil record of the São José do Rio Preto Formation comprises bone fragments, teeth, scales, shells, and coprolites, pertaining to fishes, anurans, testudines, crocodyliforms, sauropods, and theropod dinosaurs (Iori et al., 2017). Based on biostratigraphic markers, including ostracods and charophytes (e.g., Dias‐Brito et al., 2001), and radiometric dating from both underlying and overlying units (Castro et al., 2018; Dias et al., 2021), the São José do Rio Preto Formation probably was deposited during the late Santonian–early Campanian (see Navarro et al., 2025: figure S1).

Titanosaur material from the São José do Rio Preto Formation has been reported since the early 1960s (e.g., Arid et al., 1962; Arid & Vizotto, 1963). These specimens are represented mainly by isolated caudal remains, with few occurrences of cervicodorsal and appendicular elements, or more rarely associated individuals. Exceptions include the associated specimens referred to “*Antarctosaurus brasiliensis*” (Arid & Vizotto, 1971), currently regarded as a *nomen dubium* (Powell, 2003), and the nanoid saltasaurine *Ibirania parva* (Navarro et al., 2022). Additional titanosaur material from this unit includes an isolated osteoderm of a large individual (Marinho & Iori, 2011), isolated ribs with bite marks (Reis et al., 2022), and multiple morphotypes of caudal vertebrae and appendicular elements, including specimens displaying severe pathologies, such as acute osteomyelitis and blood parasites (Aureliano et al., 2026; Aureliano, Ghilardi, et al., 2021; Barbosa et al., 2019).

## 3 MATERIALS AND METHODS

### 3.1 Repository

The new specimen MPPC/GPRD 25‐013 is housed at the Museu de Paleontologia ‘Pedro Candolo’, in Uchoa Municipality, São Paulo State, Brazil, and can be accessed through a request to the collection's curators (FVI & LSP).

### 3.2 Microcomputed tomography analysis and imagery

X‐ray CT scans procedures were done on an X‐ray source microcomputed tomography Phoenix V|Tome|X M 300 (General Electric) at the Laboratório de Microtomografia of the Museu de Zoologia, Universidade de São Paulo. X‐ray projection images were recorded at 1000 ms per image, with 2000 images acquired during one slow stepwise 360° rotation of the sample, using 180 kV and 300 μA for X‐ray generation, including beam filters of 0.5 mm tin. The reconstructed image stack has an isotropic voxel size of 125 μm.

The three‐dimensional dataset was reconstructed from the digital X‐ray images to generate a volume data set, and reconstruction of volume data was done with system‐supplied Datos|x Reconstruction 3.0 (Baker Hughes Digital Solutions GmbH/Waygate Technologies), using a beam hardening correction module to reduce artifacts, using a correction factor of 2 (values varying from 0 to 10). The acquired scan data was processed on a high‐end computer HP Z8 Fury G5 Workstation with 28‐core Intel Xeon w7‐3465x 2.4 GHz (56 logical processors), 512 GB RAM, with an operating system Windows 11 PRO 64‐bit. Three‐dimensional segmentation, visualization, and analysis were performed using the software Volume Graphics VGStudio Max 2024.1 (Hexagon/Volume Graphics).

The polygonal mesh generated in VGStudio was subsequently refined in ZBrush (Pixologic) to improve surface quality and provide a better visualization of the specimen. The resulting model was virtually restored as a complete element, with the missing portions reconstructed, and was used to identify the potential positions of pneumatic diverticula. These restorations were guided by comparative anatomical comparison with more complete axes, which are detailed below.

### 3.3 Data archiving

Data reported in this article are available at Morphosource (ark:/87602/m4/783841 and ark:/87602/m4/783844) and accessible upon request. A three‐dimensional model of the specimen is freely available for visualization at the University of Michigan Online Repository of Fossils (UMORF-Model-ID=1916).

### 3.4 Anatomical description and comparisons

The anatomical and orientational terminology in the description and comparisons uses traditional or ‘Romerian’ descriptors, following Wilson (2006b). Vertebral laminae and fossae are named following the landmark‐based rationale proposed by Wilson (1999, 2012) and Wilson et al. (2011), respectively. External traits associated with axial skeleton pneumaticity were described following Zurriaguz and Cerda (2017), whereas internal architecture and pneumatic structures were described following the terminology provided by Wedel (2003). Vertebral orientations reference the horizontal as defined by the floor of the neural canal and vertical as the plane of left–right symmetry. General measurements of the specimen MPPC/GPRD 25‐013 were taken with a digital caliper (precision 0.01 mm) and are available in Table 1.

**TABLE 1:** Selected measurements (in mm) of the Vila Ventura axis (MPPC/GPRD 25‐013).

Measurement	Value
Atlantal pleurocentrum (odontoid), maximum transverse breadth	41.5
Atlantal pleurocentrum (odontoid), maximum dorsoventral height	26.4
Atlantal pleurocentrum (odontoid), maximum depth	32.0
Axial intercentrum, maximum transverse breadth of articular surface	64.0
Axial intercentrum, maximum dorsoventral height of articular surface	52.0
Axial pleurocentrum, maximum transverse breadth of posterior articular surface	78.7
Axial pleurocentrum, maximum dorsoventral height of posterior articular surface	73.3
Axial pleurocentrum, transverse breadth in the anteroventral surface	33.0
Axial centrum (axial intercentrum + pleurocentrum), minimum anteroposterior length (without odontoid)^1^	206
Axial centrum (axial intercentrum + pleurocentrum), maximum anteroposterior length (with odontoid)	232
Neural arch, maximum dorsoventral height^2^	85.2*
Neural arch, maximum anteroposterior length	143.6*
Neural spine, maximum dorsoventral height^2^	70.3*
Anterior spinal projection, maximum transverse breadth	22.7
Total dorsoventral height^2^	158.5*

*Note*: An asterisk (*) indicates measurements taken from preserved portions. (1) Measured from the ventral surface. (2) Measured from the posterior surface.

### 3.5 Cladistic analyzes

To investigate potential phylogenetic utility of axis morphology, exploratory cladistic analyses were conducted using the dataset published by Navarro et al. (2022). Twenty characters related to axial anatomy were added to the matrix (see Discussion), including those previously proposed in the literature (chars 435, 436, 440, 442, 446, 450, 451, 453) and novel ones identified in this study (chars 437–439, 441, 443–445, 447–449, 452, 454). As in Navarro et al. (2022), 24 multistate characters were treated as ordered (14, 61, 100, 102, 109, 115, 127, 132, 135, 136, 166, 179, 195, 256, 259, 276, 277, 278, 279, 299, 303, 346, 352, 354), along with two of the newly added characters (451 and 453). The final dataset comprises 454 characters scored for 107 OTUs, including specimen MPPC/GPRD 25‐013. It has been deposited in MorphoBank in NEXUS format (P6128). The data matrix (.TNT file), resulting trees (.TRE), and analysis outputs (.OUT) are available as Data S2.

The revised dataset was analyzed using TNT v.1.5 (Goloboff & Catalano, 2016). Maximum parsimony analyses were performed under two approaches: using equal weights and implied weights with a *K* value of 9 (Goloboff, 2014; Goloboff et al., 2018). In both iterations, we followed the search protocol of Poropat et al. (2021), using the “Stabilize Consensus” option in the “New Technology Search” applying sectorial searches, drift, and tree fusion algorithms, with the consensus stabilized five times, random seed set to zero, and all other search parameters left at default. It's worth mentioning that, when using this protocol, a strict consensus of all most parsimonious trees (MPTs) is automatically saved in TNT as the last tree, after running an additional round using the Tree Bisection–Reconnection (TBR) algorithm. Therefore, a separate consensus calculation is not required. The complete consensus trees and list of apomorphies are summarized in the Data S1.

### 3.6 Institutional abbreviations

AMNH, American Museum of Natural History, New York, USA; AODF, Australian Age of Dinosaurs Museum Fossil, Winton, Australia; BIBE, Big Bend National Park, Texas, USA; BYU, Brigham Young University, Provo, USA; CCG, Chengdu College of Geology, Sichuan, China; CMNH, Carnegie Museum of Natural History, Pittsburgh, USA; CPSGM, Collections Paléontologiques du Service Géologique du Maroc, Rabat, Morocco; DINO, Dinosaur National Monument, Utah, USA; GSI, Geological Survey of India, Kolkata, India; GPRD, Departamento de Geologia e Paleontologia, Universidade Estadual Paulista, São José do Rio Preto, Brazil; IGM, Geological Institute of the Mongolian Academy of Sciences, Ulaan Baatar, Mongolia; IVPP, Institute of Vertebrate Paleontology and Paleoanthropology, Beijing, China; JCMF, Jinchang Museum Fossil, Jinchang, China; KLR, Henan Geological Museum, Zhengzhou, China; MACN, Museo Argentino de Ciencias Naturales Bernardino Rivadavia, Buenos Aires, Argentina; MAU, Museo Argentino Urquiza, Rincón de los Sauces, Argentina; MB, Museum für Naturkunde der Humboldt‐Universität, Berlin, Germany; MCS, Museo de Cinco Saltos, Cinco Saltos, Argentina; MCT, Museu de Ciências da Terra, Rio de Janeiro, Brazil; MDS, Museo de Dinosaurios de Salas de los Infantes, Salas de los Infantes, Spain; MDT, Museo Desiderio Torres, Sarmiento, Argentina; MLP, Museo de La Plata, La Plata, Argentina; MMCh, Museo Municipal Ernesto Bachmann, Villa El Chocón, Argentina; MOZ, Museo Provincial de Ciencias Naturales Dr. Prof. Juan A. Olsacher, Zapala, Argentina; MPCA, Museo Provincial Carlos Ameghino, Cipolletti, Argentina; MPCN, Museo Patagónico de Ciencias Naturales, General Roca, Argentina; MPEF, Museo Paleontológico Egidio Feruglio, Trelew, Argentina; MPPC, Museu de Paleontologia ‘Pedro Candolo’, Uchoa, Brazil; MUC, Museo de la Universidade del Comahue; Neuquén, Argentina; MZSP, Museu de Zoologia da Universidade de São Paulo, São Paulo, Brazil; NMB, Staatliches Naturhistorisches Museum, Braunschweig, Germany; OMNH, Oklahoma Museum of Natural History, Norman, USA; PMU, Palaeontological Museum, Uppsala, Sweden; PVL, Fundación Miguel Lillo, Universidad Nacional de Tucumán, San Miguel de Tucumán, Argentina; SMA, Sauriermuseum Aathal, Seegräben, Switzerland; SSV, Shanshan Geological Museum, Shanshan, China; TMM, Texas Memorial Museum, Austin, USA; USNM, United States National Museum, Washington D. C., USA; UUVP, University of Utah, Salt Lake City, USA; YPM, Yale Peabody Museum, New Haven, USA; ZDM, Zigong Dinosaur Museum, Zigong, China.

## 4 RESULTS

### 4.1 Systematic paleontology

dinosauria Owen, 1842.

sauropoda Marsh, 1878.

titanosauriformes Salgado et al., 1997.

somphospondyli Wilson & Sereno, 1998.

titanosauria Bonaparte & Coria, 1993.

lithostrotia Upchurch et al., 2004.

*Gen. et* *sp*. *indet*. (Figures 2, 3, 4, 5, 6 and Table 1).

**FIGURE 2:**
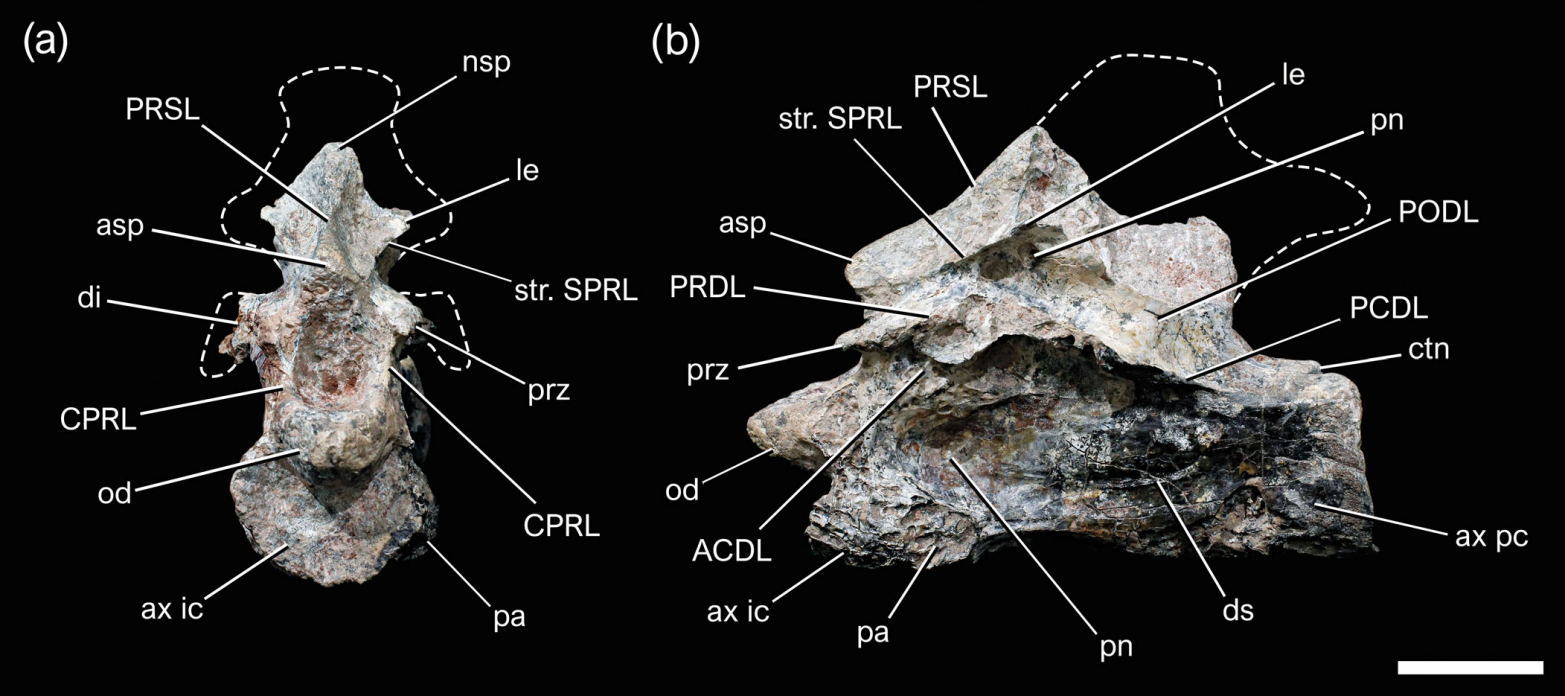
The Vila Ventura axis (MPPC/GPRD 25‐013) in anterior (a) and left lateral (b) views. Dashed lines represent reconstructed areas, based on the morphology of closely related taxa. Abbreviations: ACDL, anterior centrodiapophyseal lamina; asp, anterior spinal projection; ax ic, axial intercentrum; ax pc, axial pleurocentrum; CPRL, centroprezygapophyseal lamina; ctn, cotylar notch; di, diapophysis; ds, diverticulum scar; le, lateral expansion; nsp, neural spine; od, odontoid; pa, parapophysis; PCDL, posterior centrodiapophyseal lamina; pn, pneumatopore; PODL, postzygodiapophyseal lamina; PRDL, prezygodiapophyseal lamina; PRSL, prespinal lamina; prz, prezygapophysis; str. SPRL, stranded spinoprezygapophyseal lamina. Scale bar equals 5 cm.

**FIGURE 3:**
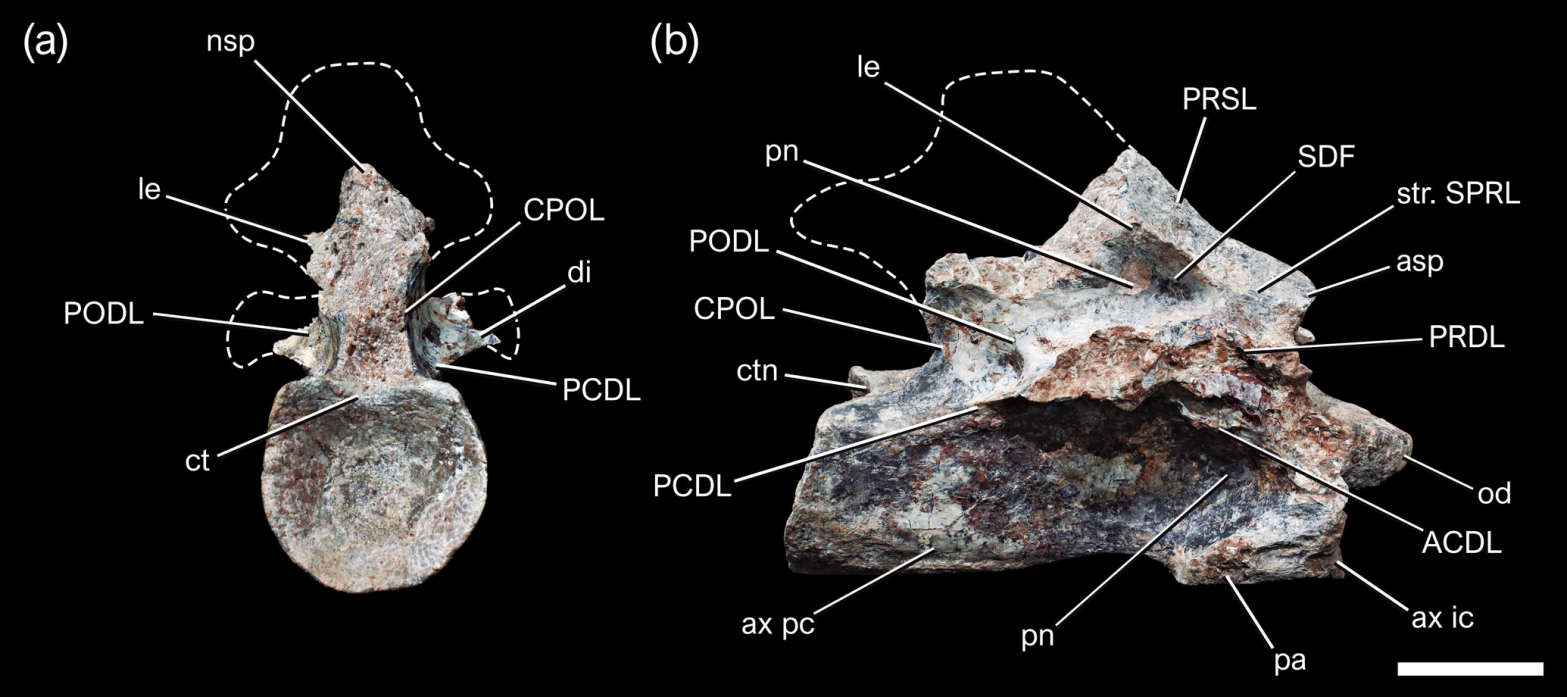
The Vila Ventura axis (MPPC/GPRD 25‐013) in posterior (a) and right lateral (b) views. Dashed lines represent reconstructed areas, based on the morphology of closely related taxa. Abbreviations: ACDL, anterior centrodiapophyseal lamina; asp, anterior spinal projection; ax ic, axial intercentrum; ax pc, axial pleurocentrum; CPOL, centropostzygapophyseal lamina; ctn, cotylar notch; di, diapophysis; ds, diverticulum scar; le, lateral expansion; nsp, neural spine; od, odontoid; pa, parapophysis; PCDL, posterior centrodiapophyseal lamina; pn, pneumatopore; PODL, postzygodiapophyseal lamina; PRDL, prezygodiapophyseal lamina; PRSL, prespinal lamina; prz, prezygapophysis; SDF, spinodiapophyseal fossa; str. SPRL, stranded spinoprezygapophyseal lamina. Scale bar equals 5 cm.

**FIGURE 4:**
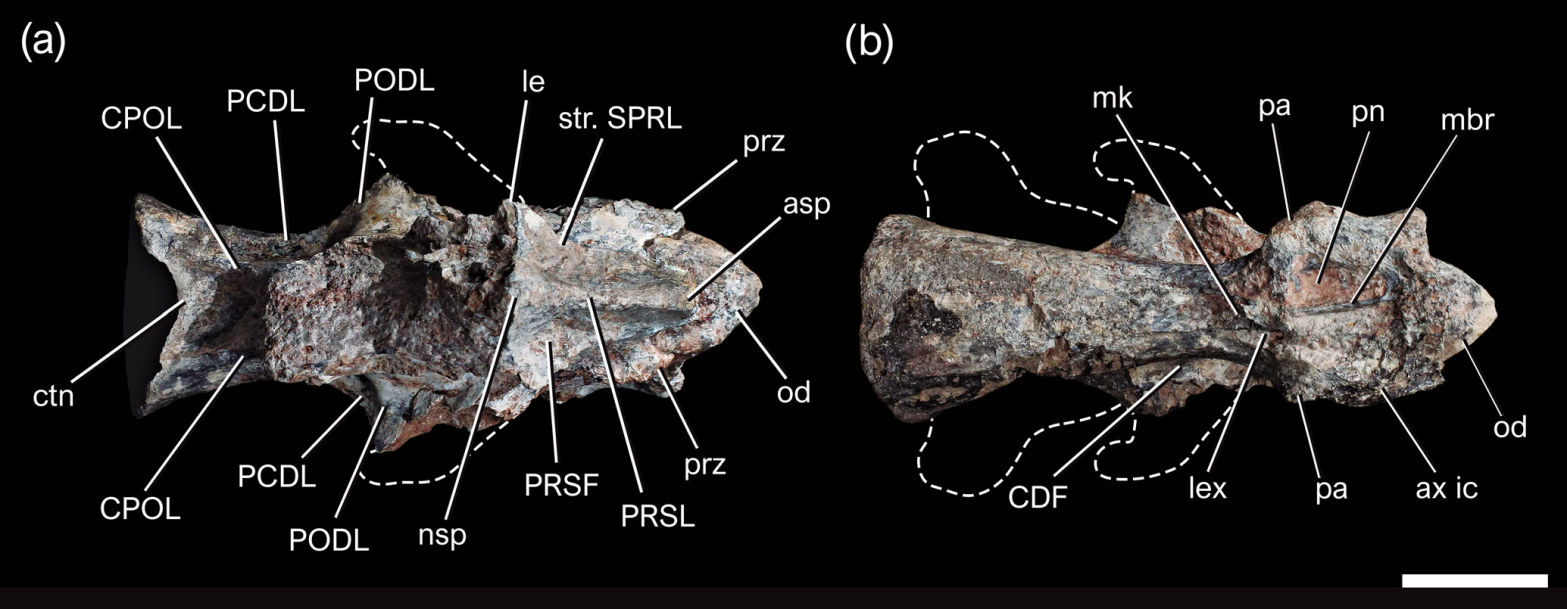
The Vila Ventura axis (MPPC/GPRD 25‐013) in dorsal (a) and ventral (b) views. Dashed lines represent reconstructed areas, based on the morphology of closely related taxa. Abbreviations: asp, anterior spinal projection; ax ic, axial intercentrum; CDF, centrodiapophyseal lamina; CPOL, centropostzygapophyseal lamina; ctn, cotylar notch; le, lateral expansion; lex, lateral excavation; mbr, median bony rim; mk, midline ventral keel; nsp, neural spine; od, odontoid; pa, parapophysis; PCDL, posterior centrodiapophyseal lamina; pn, pneumatopore; PODL, postzygodiapophyseal lamina; PRSF, prespinal fossa; PRSL, prespinal lamina; prz, prezygapophysis; str. SPRL, stranded spinoprezygapophyseal lamina. Scale bar equals 5 cm.

**FIGURE 5:**
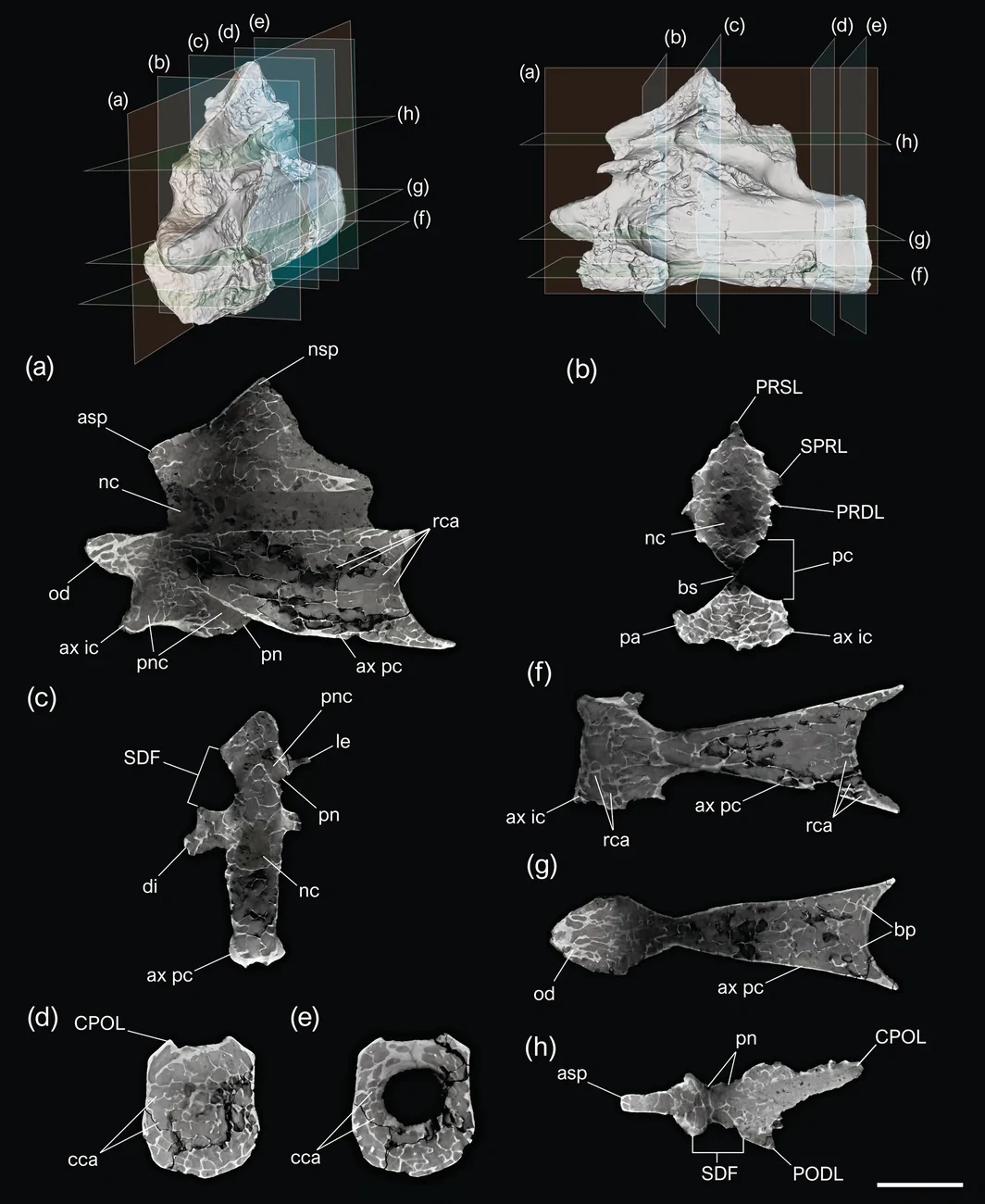
Microcomputed tomography of the Vila Ventura axis (MPPC/GPRD 25‐013). Slices in sagittal/longitudinal (a), frontal (b–e), and transverse (f–h) planes. Abbreviations: asp, anterior spinal projection; ax ic, axial intercentrum; ax pc, axial pleurocentrum; bp, bony plate; bs, bony septum; cca, circumferential camellae; CPOL, centropostzygapophyseal lamina; di, diapophysis; le, lateral expansion; nc, neural canal; nsp, neural spine; od, odontoid; pa, parapophysis; pc, pleurocoelic fossa; pn, pneumatopore; pnc, pneumatic canal; PODL, postzygodiapophyseal lamina; PRDL, prezygodiapophyseal lamina; PRSL, prespinal lamina; rca, radial camellae; SDF, spinodiapophyseal fossa; SPRL, spinoprezygapophyseal lamina. Scale bar equals 5 cm.

**FIGURE 6:**
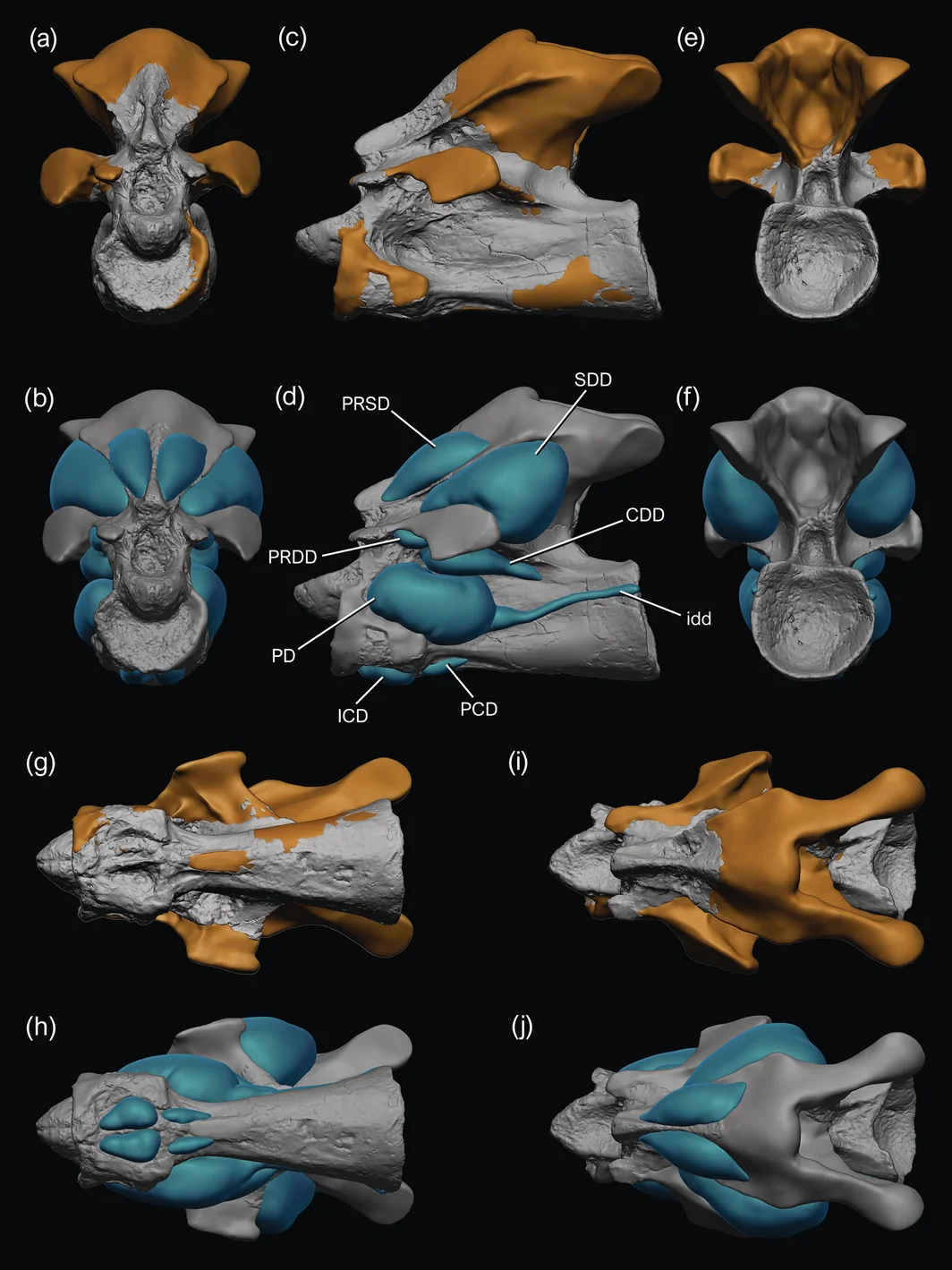
Digital reconstruction of the Vila Ventura axis (MPPC/GPRD 25‐013) and reconstruction of air sac diverticula in anterior (a–b), left lateral (c–d), posterior (e–f), ventral (g–h), and dorsal (i–j) views. The distribution of air sac diverticula is inferred from both external and internal pneumatic features of the specimen, whereas reconstructed portions were modeled based on closely related taxa (see discussion). Abbreviations: CDD, centrodiapophyseal diverticulum; ICD, intercentral diverticulum; idd, interdiverticular duct; PCD, pleurocentral diverticulum; PD, pleurocoelic diverticulum; PRDD, prezygodiapophyseal diverticulum; PRSD, prespinal diverticulum; SDD, spinodiapophyseal diverticulum.

#### 4.1.1 Referred specimen

MPPC/GPRD 25‐013, an almost complete axis lacking only the posterodorsal portion of the neural spine and the postzygapophyses.

#### 4.1.2 Locality

Vila Ventura Paleontological Area, a rural zone between the municipalities of Ibirá, Uchoa, and Cedral, northeastern São Paulo State, Brazil (Figure 1c).

#### 4.1.3 Stratigraphic horizon and age

The Vila Ventura axis was preserved in a carbonate‐cemented conglomeratic sandstone of the São José do Rio Preto Formation (Bauru Group, Paraná Basin). This unit is constrained in the late Santonian to early Campanian interval (Dias‐Brito et al., 2001).

### 4.2 Description

#### 4.2.1 Preservation

The Vila Ventura axis is nearly complete, lacking only the posterodorsal portion of the neural arch. It shows no apparent taphonomic distortion. However, it is damaged in several areas, including most of the left side of the intercentrum and parapophysis, and small portions of the pleurocentrum. It is missing most of the right prezygapophysis, the distal end of the diapophyses and the postzygapophyses, as well as the dorsalmost portion of the neural spine (Figures 2, 3, 4).

#### 4.2.2 Ontogenetic assessment

The Vila Ventura axis is a single element that appears to be fully co‐ossified at its respective points of contact. There are no discernible sutures between the atlantal pleurocentrum (i.e., the odontoid), axial intercentrum, axial pleurocentrum, and axial neural arch. Boundaries between these elements are inferred here at their approximate positions, and we refer to these parts individually in the description below.

The degree of fusion within and among vertebral elements has been used as a means to assess ontogenetic maturity, but problems emerge when the sequence of fusion for a species is not known or ambiguous, or when only single elements are available (see Griffin et al., 2021; Ikejiri et al., 2005; Irmis, 2007). In cases where the fusion sequence is known, however, isolated elements can be informative when their fusional state and expected timing of fusion combine to provide unambiguous evidence of relative age. For example, an unfused element from a region that typically fuses early in the ontogenetic sequence indicates a young individual, and conversely, a fused element from a region that typically fuses late in ontogeny indicates an older individual.

The sequence of vertebral fusion in titanosaurs is best known in *Bonitasaura salgadoi*, from the Upper Cretaceous (Santonian) of Argentina (Apesteguía, 2004). In the holotypic specimen (MPCA 460), the neurocentral suture is fully fused in the axis and caudal vertebrae but not in the postaxial cervical and dorsal vertebrae (Gallina, 2011; Gallina & Apesteguía, 2015). Bone microstructural features in that individual indicate a histological ontogenetic stage of 9 (HOS sensu Klein & Sander, 2008), which is towards the middle of the ontogenetic trajectory (Gallina, 2012). This state of fusion matches that in the Vila Ventura axis, suggesting that individual is at least nearly mature. Nevertheless, we cannot yet say whether it has attained somatic maturity. Further histological analyses are necessary to determine the HOS of this specimen.

#### 4.2.3 Centrum

The odontoid process (i.e., atlantal pleurocentrum) is approximately twice as broad transversely as it is dorsoventrally tall and slightly longer anteroposteriorly than it is tall (Figures 2 and 3 and Table 1). It is crescent‐shaped in anterior view, with a convex ventrolateral margin and concave dorsal margin, with a sagittal groove marking its ventral surface (Figure 4). There is no trace of a suture between the odontoid and axial pleurocentrum, but on the dorsolateral margins of their presumed junction, well‐developed centroprezygapophyseal laminae emerge and extend towards the neural arch forming sharp edges around the neural canal. The dorsal margin of the odontoid is contiguous with the floor of the neural canal (Figure 4a), which is ellipsoid in shape and taller than it is wide (Figure 2).

The axial intercentrum has an approximately crescentic outline and is roughly 20% broader transversely than it is dorsoventrally tall (Figure 2a). The articular facet for the atlantal intercentrum is mildly concave transversely and has an ellipsoid shape. The ventrolateral margins of the axial intercentrum are expanded and contiguous with the parapophyses, which are positioned just posterior to them. In ventral view, the intercentrum has approximately 75% the transverse breadth of the posterior articular surface of the pleurocentrum (Figure 4b and Table 1).

The conjoined axial intercentrum and pleurocentrum is relatively elongate, with its length more than three times larger than its posterior height—and slightly less than three times as long if the odontoid process is not considered (Table 1). In ventral view, the pleurocentrum appears strongly constricted transversely just posterior to the parapophyses, acquiring a goblet‐like shape. Anterior to this constriction, a blunt midline ventral keel is present, in which it bifurcates into two sharp ridges towards the posterior centrum, delimiting a ‘V' shaped excavation (Figure 4b). The centrum is rectangular in lateral view and predominated by a pleurocoelic fossa that deepens anteriorly and ends in a well‐defined pneumatopore surrounded by a subcircular fossa (Figures 2b and 3b). The lateral pneumatopores extend interiorly nearly to the midline of the centrum, where they are separated from their opposites by a thin median bony wall.

The parapophysis is positioned just ventral to the deepest part of the pleurocoel. It appears to be shared between the axial intercentrum and axial pleurocentrum, but the relative contribution of each is not known. The transverse expansion of the parapophysis, visible in ventral view, extends anteriorly to the margin of the axial intercentrum (Figure 4b). The limits between the parapophysis and intercentrum are not well preserved on either side, and so its anteroposterior length is not known with certainty. In the right parapophysis, small coel‐like excavations have developed, forming a ridge that extends anteriorly to the articular facet of the intercentrum (Figure 3b).

The posterior concavity, or cotyle, is as tall as it is wide (Table 1). Its outline in posterior view is rounded ventrally but flattened laterally and dorsally (Figure 3a). The cotyle is smoothly concave, with its depth representing approximately half its height or width. Its dorsal margin is marked by a broad notch that is visible in dorsal view (Figure 4a). In lateral view, the cotyle is slightly canted anteriorly relative to the horizontal plane (Figures 2 and 3).

#### 4.2.4 Neural arch

The neural arch is almost triangular in lateral and dorsal views (Figures 3a and 4a). As preserved, the neural arch occupies at least 70% of the pleurocentrum length, but with the postzygapophyses it would have been subequal in length (Table 1). At its tallest preserved point, the neural arch exceeds the height of the posterior centrum. From that point, it tapers anteriorly, following the median prespinal lamina, reaching a height that is less than that of the posterior centrum. The infradiapophyseal portion of the neural arch is marked by a deep centrodiapophyseal fossa, of which the roof is formed mostly by the posterior centrodiapophyseal lamina (Figures 2b and 3b).

The prezygapophysis is better preserved on the left side, where it is relatively well developed (preserved dimensions: 45 mm × 19 mm). It is hook‐shaped in dorsal view and extends to the lateral margin of the intercentrum in lateral view (Figure 2b). The prezygapophysis is supported by a centroprezygapophyseal lamina that extends to the dorsolateral margin of the odontoid process and the dorsolateral margin of the intercentrum (Figure 2). The prezygodiapophyseal lamina extends as a stout ridge contacting the diapophyses anterodorsally. By contrast, the centropostzygapophyseal laminae emerge as sharp structures that extend posteriorly to the dorsolateral margins of the cotyle. However, most of their dorsal development is unknown due to the postzygapophyses not being preserved (Figure 3).

The diapophyses are incomplete, but their corresponding laminae (i.e., ACDL, PCDL, PRDL, and PODL) are preserved and discernible. Despite their incompleteness, they appear to extend farther laterally than the parapophyses and above the level of the neurocentral junction (Figures 2 and 3). The preserved portions suggest a posterolateral development of the diapophyses, indicating the absence of bicapitate axial ribs, although these are not preserved in the specimen. The anterior centrodiapophyseal laminae extend ventral to the prezygodiapophyseal laminae, although they are very damaged on both sides. The posterior centrodiapophyseal lamina is sharp but anteroposteriorly short, and it strongly projects laterally. In turn, the postzygodiapophyseal lamina emerges as a stout prominence that intersects the posterior centrodiapophyseal lamina and fades towards its dorsal portion (Figures 2b and 3b).

Spinoprezygapophyseal laminae are well‐developed, emerging in a stranded pattern parallel to the lateral profile of the neural spine (Figures 2 and 3). Hence, this lamina does not contact the prezygapophysis nor the prezygodiapophyseal lamina but extends anteriorly until the ventrolateral surface of an anterior spinal projection. The spinoprezygapophyseal laminae form sharp ridges, projecting laterally into pointed lateral processes near the level of the neural spine (Figure 2a). Dorsally, these laminae serve as the floor of a shallow but well‐defined prespinal fossa, which is divided sagitally by a robust prespinal lamina (Figure 4a). The prespinal lamina thickens posteriorly towards the neural spine, whereas anteriorly it projects close to the level of the prezygapophysis and forms a prominent anterior spinal projection. In anterior view, this process is broad and represents 50% of the odontoid breadth (Figure 2a). The neural spine is incomplete but appears to be pyramidal in dorsal view, probably being slightly longer anteroposteriorly than it is broad (Figure 4a).

#### 4.2.5 Pneumaticity

The Vila Ventura axis displays multiple features indicative of a high degree of postcranial skeletal pneumaticity, both on its external surface and internal microstructure. Externally, the pleurocentrum exhibits well‐defined lateral pneumatopores within deep pleurocoels (Figures 2b and 3b), accompanied posteriorly by grooves that potentially represent paths of air sac diverticula. These grooves, which are more evident on the left side, traverse the lateral surface of the centrum and extend posterodorsally onto the posterior centrodiapophyseal lamina (Figure 2a). Anterior to the pleurocoels, both sides of the centrum also display additional excavations in the transition between the axial intercentrum and pleurocentrum, lateral to the median keel of the pleurocentrum (Figure 4b), as well as anterior to the right parapophysis and left diapophysis. Ventrally, the intercentrum has enlarged paired fossae that are ellipsoid in shape and separated by a sharp median rim.

The diapophyseal fossae on the neural arch are well‐marked but relatively shallow (Figures 2b and 3b). The centrodiapophyseal fossa is triangular in shape, framed anteroventrally by the dorsal rim of the pleurocoel and dorsally by the diapophyseal process and the posterior centrodiapophyseal lamina, and lacks evident pneumatic foramina. The spinodiapophyseal fossae, in turn, are symmetrically perforated anteriorly with a small pneumatopore, accompanied posteriorly by a large and D‐shaped one (Figures 2b and 3b). The prespinal fossa appears as a marked depression, parallel and between the spinoprezygapophyseal and the median prespinal laminae, and it is pierced by several foramina on each of them (Figure 4a). In contrast, the postzygapophyseal‐centrodiapophyseal fossa develops as a broad but shallow depression, being dorsoventrally tall and without pneumatic foramina (Figures 2b and 3).

The internal structure of the Vila Ventura axis has a bone tissue with camellate‐type structure, characterized by an irregular array of small, angular air spaces bounded by thin trabeculae (Figure 5). Bone cortex is thicker in the odontoid and ventral surface of centrum, compared to similar areas in the neural arch (Figure 5a). In the axial intercentrum, the trabeculae form a scaffold‐like pattern, organized in both vertical and horizontal planes (Figure 5b). The camellae are transversely elongated, dorsoventrally compacted, and comparatively smaller (ca. 5–7 mm diameter) than those in the odontoid (ca. 10–12 mm diameter) and axial pleurocentrum (up to 16 mm diameter). Both the odontoid and axial pleurocentrum exhibit large, anteroposteriorly elongated camellae (up to 34 mm length; Figure 5c–f). In the cotyle, architectural patterns are characterized by the presence of radial camellae interspersed with incipient internal bony plates (Figure 5d, e). In the vertical plane, these appear as circumferential camellae entering into the axial pleurocentrum one‐third of its anteroposterior length (Figure 5f). The large external pneumatopores present on the intercentrum and spinodiapophyseal fossae (Figure 5a,c,g) correspond internally to the development of pneumatic canals interconnecting major chambers within camellae. These structures likely form an extensive pneumatic network that may have communicated directly with air sac diverticula (Figure 6).

## 5 DISCUSSION

### 5.1 Comparisons and systematic affinities

Sauropod axes exhibit considerable morphological and morphometric variation (Table 2), highlighting the phylogenetic diversity and different functional adaptations in the group. The Vila Ventura axis (MPPC/GPRD 25‐013) is a nearly complete element, allowing direct comparisons with other sauropod taxa (Figure 7). Its morphology, particularly the general structure of the intercentrum, neural arch, and internal pneumatic architecture, provides a meaningful basis for assessing its potential phylogenetic affinities.

**TABLE 2:** Selected metrics of sauropod axes ordered (in ascending order) by their aEI values.

Taxon	Specimen	CL^a^	CW^b^	CH^b^	TH^b^	CL:TH	CW:CH	CL:CW^c^	CL:CH^d^	aEI^e^
**Basal Sauropoda**										
*Spinophorosaurus nigerensis*	NMB‐1699‐R	155.5	69.0	71.0	148.0	1.1	1.0	2.3	2.2	2.2
*Tazoudasaurus naimi*	CPSGM To1‐239	121.0*	50.0	49.0	145.0	0.8	1.0	2.4	2.5	2.4
**Eusauropoda**										
*Shunosaurus lii*	ZDM 5402	125.0	49.0	65.0	150.0	0.8	0.8	2.6	1.9	2.2
*Jinchuanloong niedu*	JCMF 0132	80.0	–	35.0	90.5**	0.9	–	–	2.3	2.3
*Bagualia alba*	MPEF‐PV 3301/13	110.0	44.0	50.0	150.0	0.7	0.9	2.5	2.2	2.3
*Patagosaurus fariasi*	MACN‐CH 936	157.6**	–	64.7**	167.2**	0.9	–	–	2.4	2.4
**Mamenchisauridae**										
*Xinjiangtitan shanshanesis*	SSV120001	165.0	115.0	95.0	205.0	0.8	1.2	1.4	1.7	1.6
*Mamenchisaurus sinocanadorum*	IVPP V10603	187.3	95.9	89.4	159.2**	1.2	1.1	2.0	2.1	2.0
*Mamenchisaurus hochuanensis*	CCG V 20401	160.0	75.0	80.0	175.0	0.9	0.9	2.1	2.0	2.1
*Omeisaurus tianfuensis*	ZDM 5701	185.0	49.0	64.0	113.0	1.6	0.8	3.8	2.9	3.3
**Turiasauria**										
*Moabosaurus utahensis*	BYU 10815	148.2**	45.5**	68.6**	223.3**	0.7	0.7	3.3	2.2	2.6
**Dicraeosauridae**										
*Amargasaurus cazaui*	MACN‐N 15	119.4	63.0	66.4	300*	0.4	0.9	1.9	1.8	1.8
*Smitanosaurus agilis*	USNM 5384	61.0	29.0	32.0	98.0	0.6	0.9	2.1	1.9	2.0
*Bajadasaurus pronuspinax*	MMCh‐PV 75	93.0	45.0	40.0	115.0	0.8	1.1	2.1	2.3	2.2
*Dicraeosaurus hansemanni*	MB.R.4886	160.0	71.0	67.0	273.0	0.6	1.1	2.3	2.4	2.3
**Diplodocidae**										
*Apatosaurus louisae*	CMNH 3018	190.0	85.0	100.0**	–	–	0.9	2.2	1.9	2.1
*Diplodocus carnegii*	CMNH 84	165.0	54.0	78.0**	171.0	1.0	0.7	3.1	2.1	2.5
*Galeamopus hayi*	AMNH 969	200.5**	67.9**	63.7**	189.2**	1.1	1.1	3.0	3.1	3.0
*Kaatedocus siberi*	SMA 0004	87.9**	31.4**	25.1**	89.6**	1.0	1.3	2.8	3.5	3.1
**Rebbachisauridae**										
Zapala axis	MOZ‐Pv 088	92.0	31.6	35.5	143.0	0.6	0.9	2.9	2.6	2.7
*Demandasaurus darwini*	MDS‐RVII.606	100.0	34.0	38.0*	180.0	0.6	0.9	2.9	2.6	2.8
*Lavocatisaurus agrioensis*	MOZ‐Pv 1232	118.0**	49.1**	31.7**	150.0*, **	0.8	1.5	2.4	3.7	2.9
**Camarasauridae**										
*Camarasaurus grandis*	YPM 1905	129.8**	86.0**	81.8**	221.5**	0.6	1.1	1.5	1.6	1.5
*Camarasaurus* sp. indet.	UUVP 6341	161.0	121.0	85.0	264.0	0.6	1.4	1.3	1.9	1.6
*Camarasaurus supremus*	AMNH 5761	235.0	132.0**	122.0	372.0**	0.6	1.1	1.8	1.9	1.9
*Camarasaurus lentus*	DINO 088	165.3**	96.3**	67.2**	211.9**	0.8	1.4	1.7	2.5	2.0
*Camarasaurus lewisi*	BYU 9047	155.0	75.0	73.0	–	–	1.0	2.1	2.1	2.1
**Brachiosauridae**										
*Giraffatitan brancai*	MB.R.2180.23	203.0	83.0*	99.0	249.0	0.8	0.8	2.4	2.1	2.2
*Abydosaurus mcintoshi*	DINO 16488	180.0	83.1	–	–	–	–	2.2	–	2.2
**Euhelopodidae**										
*Yunmenglong ruyangensis*	KLR‐07‐50‐31	250.0	–	130.0	325.3**	0.8	–	–	1.9	1.9
*Mongolosaurus haplodon*	AMNH 6710	167.0	70.0*	76.0*	–	–	0.9	2.4	2.2	2.3
*Euhelopus zdanskyi*	PMU.R233a	94.0	33.0	37.0	132.0	0.7	0.9	2.8	2.5	2.7
*Erketu ellisoni*	IGM 100/1803	160.0	50.0	49.0	127.3**	1.3	1.0	3.2	3.3	3.2
**Titanosauria**										
*Neuquensaurus australis*	MCS‐PV 174‐80	44.3	40.0	33.1	104.9	0.4	1.2	1.1	1.3	1.2
*Alamosaurus sanjuanensis*	TMM 43621‐1	135.0**	85.0**	120.0**	–	–	0.7	1.6	1.1	1.3
*Diamantinasaurus matildae*	AODF 836	113.0	71.0	84.0	211.0	0.5	0.8	1.6	1.3	1.5
*Saltasaurus loricatus*	PVL 4017‐1	55.3	38.7	35.6	114.0	0.5	1.1	1.4	1.6	1.5
*Neuquensaurus australis*	MLP‐CS 1311	49.6	32.6	29.9	115.2	0.4	1.1	1.5	1.7	1.6
Nand axis	196/CRP/GSI/05	126.0	63.0	57.0	136.0	0.9	1.1	2.0	2.2	2.1
*Bonitasaura salgadoi*	MPCA 460	135.0	54.5*	57.0	182.0	0.7	1.0	2.5	2.4	2.4
*Sarmientosaurus musacchioi*	MDT‐PV 2	102.0*	40.7	45.5*	143.8*	0.7	0.9	2.5	2.2	2.4
Roca axis A	MPCN unnumbered	166.0	71.6	61.2	153.0	1.1	1.2	2.3	2.7	2.5
Peirópolis axis B	MCT.R. unnumbered	93.0**	37.5**	34.3**	94.0**	1.0	1.1	2.5	2.7	2.6
*Pitekunsaurus macayai*	MAU‐Pv‐AG‐446/3	116.0	50.8	39.2	103.7	1.1	1.3	2.3	3.0	2.6
Peirópolis axis A (Series A)	MCT.R.1487	103.0**	41.0**	35.3**	93.1**	1.1	1.2	2.5	2.9	2.7
Vila Ventura axis	MPPC/GPRD 25‐013	206.0	78.7	73.3	158.5*	1.3	1.1	2.6	2.8	2.7
Roca axis B	MPCN unnumbered	149.0	63.7*	42.3*	130.0*	1.1	1.5	2.3	3.5	2.8
*Futalognkosaurus dukei*	MUCPv‐323	245.0	84.0	87.0*	216.0*	1.1	1.0	2.9	2.8	2.9
*Inawentu oslatus*	MAU‐PV‐LI‐595	144.0	41.0*	53.0	–	–	0.8	3.5	2.7	3.1
*Tapuiasaurus macedoi*	MZSP‐PV 807	163.0	49.0	46.0	128.0	1.3	1.1	3.3	3.5	3.4

*Note*: An asterisk (*) marks the measurements based on preserved portions, while a double asterisk (**) corresponds to measurements taken from scaled photographs (ImageJ), and a dash (−) denotes when the region is not preserved or indicates measurements that could not be taken.

Abbreviations: aEI, average Elongation Index; CH, centrum height; CL, centrum length; CW, centrum width; TH, total height.

^a^ Measured from ventral surface (excluding the odontoid process).

^b^ Measured from posterior surface.

^c^ Elongation Index sensu Upchurch (1998).

^d^ Elongation Index sensu Wilson and Sereno (1998).

^e^ Average Elongation Index sensu Chure et al. (2010), corresponds to the anteroposterior length of centrum divided by mean average value of the transverse width and dorsoventral height of posterior articular surface of the centrum (cotyle).

**FIGURE 7:**
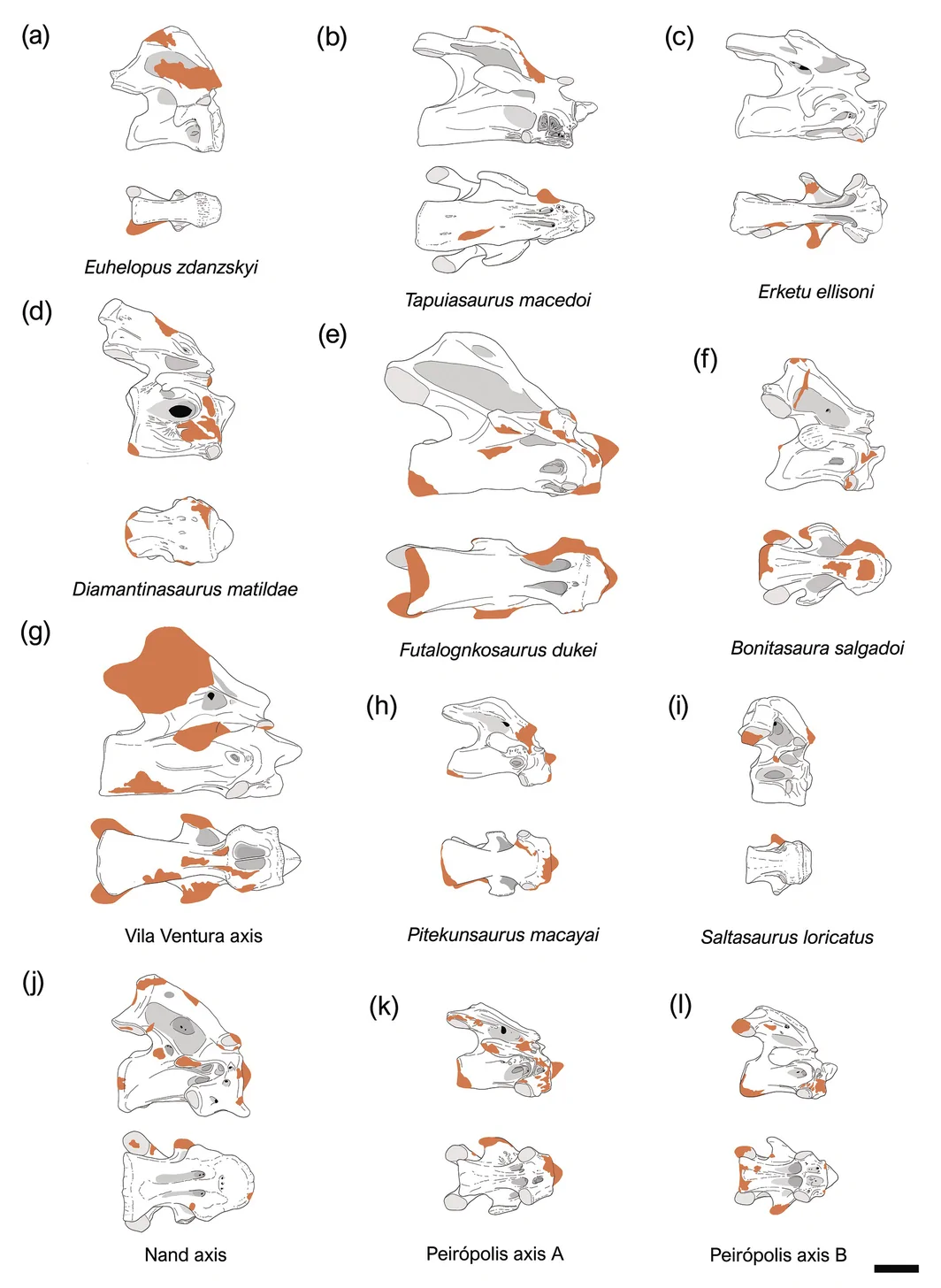
Comparisons (to scale) among selected somphospondylian axes in right lateral (top) and ventral (bottom) views: (a) *Euhelopus zdanskyi* (PMU.R233*); (b) *Tapuiasaurus macedoi* (MZSP‐PV 807*); (c) *Erketu ellisoni* (IGM 100/1803); (d) *Diamantinasaurus matildae* (AODF 836*); (e) *Futalognkosaurus dukei* (MUCPv‐323); (f) *Bonitasaura salgadoi* (MPCA 460*); (g) Vila Ventura axis (MPPC/GPRD 25‐013*); (h) *Pitekunsaurus macayai* (MAU‐Pv‐AG‐446/3); (i) *Saltasaurus loricatus* (PVL 4017‐1); (j) Nand axis (196/CRP/GSI/05); (k) Peirópolis axis A (“DGM Series A", MCT.R.1487); (l) Peirópolis axis B (MCT.R. unnumbered*). Asterisks above indicate reversed views, although reconstructions are based on the most complete structures preserved on either side. Gray tones indicate fossae and articular surfaces, while orange tones represent reconstructed damaged areas. Taxa are ordered in the rows in decreasing order of their geological age. Scale bar equals 5 cm.

The atlantal pleurocentrum (i.e., odontoid) in the Vila Ventura axis is as broad as the anteroposterior length of the intercentrum and projects anteriorly to an equivalent extent (Table 1). This condition contrasts with the anteroposteriorly abbreviated odontoid observed in saltasaurines (Zurriaguz et al., 2025; Zurriaguz & Powell, 2015), the Nand axis (Wilson & Mohabey, 2006), and *Euhelopus zdanskyi* (Wilson & Upchurch, 2009). Moreover, the odontoid of the Vila Ventura axis bears a ventral longitudinal groove, likely representing an autapomorphic condition of this morphotype.

In ventral view, the intercentrum of the Vila Ventura axis exhibits two broad and deep fossae separated by a thin bony septum, a condition also observed in MCT.R.1487, identified as ‘DGM Series A’ by Powell (1987, 2003) (Figure 7k), and in a second, distinct axis collected from the same outcrop (hereafter, Peirópolis axis B; MCT.R. unnumbered; Figure 7l). Both correspond to unnamed taxa from late Maastrichtian deposits of the Serra da Galga Formation (Bauru Group) at the Mumbuca site, located along the BR‐262 Highway, Peirópolis District, Minas Gerais State, Brazil (Campos & Kellner, 1999). However, the Vila Ventura axis can be distinguished from the MCT.R specimens by the development of such fossae, as well as by its proportionally wider intercentrum (Figure 7g), measuring approximately 75% of the transverse width of the posterior cotyle and one‐third of the overall anteroposterior length of the centrum (Table 1).

The Vila Ventura axis bears a midline ventral keel that originates at the presumed junction with the intercentrum and extends to just before the mid‐length of the pleurocentrum (Figure 7). Similar conditions are observed in *Camarasaurus* spp. (Madsen et al., 1995), brachiosaurids (e.g., *Giraffatitan brancai*; Janensch, 1950), in some somphospondylians (e.g., *Erketu ellisoni*; Ksepka & Norell, 2006), and titanosaurs (e.g., *Tapuiasaurus macedoi*, *Futalognkosaurus dukei*, *Inawentu oslatus*, and both axes from Peirópolis; pers. obs.). As in the Nand axis, the midline ventral keel in the Vila Ventura axis is developed as a low ribbon‐like ridge, rather than a sharp rim (Figure 7j). Ventrolateral excavations are associated with this longitudinal median ridge, although these structures are more developed in the aforementioned taxa than in the Vila Ventura axis (Figure 7). These conditions differ from the morphology present in *Bonitasaura* and *Neuquensaurus australis*, where a ventral keel is restricted to a pointed eminence positioned posteriorly and appressed to the cotyle (Gallina & Apesteguía, 2015; Zurriaguz et al., 2025). These conditions also differ from *Diamantinasaurus matildae* and *Saltasaurus loricatus* which an incipient median ventral ridge extends along the entire length of the pleurocentrum (Poropat et al., 2021; Zurriaguz & Powell, 2015).

The posterior cotyle of the Vila Ventura axis bears a broad notch on its dorsal margin (Figure 3). This subtle feature is usually observed in postaxial cervical vertebrae, and it is present in basally diverging macronarians (e.g., *Europasaurus holgeri*; Carballido & Sander, 2014), non‐titanosaur somphospondylians (e.g., *Euhelopus*; Wilson & Upchurch, 2009), and saltasaurine titanosaurs, such as *Rocasaurus muniozi*, *Saltasaurus*, and *Ibirania* (Navarro et al., 2022). This condition is also observed in the anterior dorsal vertebra of *Diamantinasaurus* (Poropat et al., 2015).

The parapophyses in the Vila Ventura axis are elliptical and transversely wide and originate at the presumed transition between the intercentrum and the pleurocentrum, as in *Erketu*, *Futalognkosaurus*, *Bonitasaura*, *Pitekunsaurus macayai*, the Nand axis, and the Peirópolis axes (Figure 7). This condition diverges from the condition observed in somphospondylians (e.g., *Mongolosaurus haplodon*; Mannion, 2011), early titanosaurs (e.g., *Tapuiasaurus*; pers. obs.), and saltasauroids (e.g., *Diamantinasaurus*, *Neuquensaurus*, and *Saltasaurus*), in which the parapophyses are rounded in shape, restricted to the intercentrum, and laterally projecting (Figure 7).

Although not fully preserved, the diapophyses of the Vila Ventura axis appear trapezoidal in shape and extend posterolaterally, resembling those of *Tapuiasaurus* and other colossosaurs (e.g., *Futalognkosaurus*, *Bonitasaura*, *Pitekunsaurus*; pers. obs.). This condition suggests the presence of unicapitate ribs, as preserved in *Tapuiasaurus* (pers. obs.) and a specimen of *Diamantinasaurus* (AODF 0906; see Poropat et al., 2023, figure 21). This condition contrasts with the ventrally developed diapophyses present in non‐titanosaur somphospondylians (e.g., *Euhelopus*, *Erketu*) and saltasaurines (e.g., *Neuquensaurus*, *Saltasaurus*), suggesting the articulation of bicapitate ribs, as in post‐axial cervical vertebrae (see Zurriaguz et al., 2025, figure 3).

The prezygapophyses in the Vila Ventura axis project anteriorly, as in *Yunmenglong ruyangensis*, *Erketu*, *Tapuiasaurus*, and the Peirópolis axis B. This condition differs from most sauropods, in which axial prezygapophyses are limited to articular facets closely appressed to the neural arch. In the Vila Ventura axis, the prezygodiapophyseal lamina and the anterior centrodiapophyseal lamina are marked ridges, but they remain separate along their extent, as in *Futalognkosaurus*. This contrasts with both laminae merging into a single, ribbon‐like structure, as in *Tapuiasaurus*, *Bonitasaura*, *Pitekunsaurus*, the Peirópolis axes, and saltasauroids.

The spinoprezygapophyseal laminae are laterally expanded and exhibit a stranded pattern in the Vila Ventura axis. They do not merge with the lateral margins of the neural spine nor approach the lateral surface of the prezygapophyses. Similar lateral expansions are present in the axial neural spines of *Pitekunsaurus* and in the Peirópolis axis A, but in these taxa they represent bulging outgrowths rather than sharply laminar prominences as in the Vila Ventura axis, as well as their spinoprezygapophyseal laminae extending towards the prezygapophyses. Hence, this pattern is regarded here as autapomorphic for the Vila Ventura axis.

The prespinal lamina is robust and thickens dorsally towards the neural spine as in *Futalognkosaurus*, despite being slender in the Vila Ventura axis than the latter. In the Vila Ventura axis, this lamina protrudes anteriorly and forms an anterior spinal projection. Although this projection occurs in several sauropods (e.g., *Bagualia alba*, *Xinjiangtitan shanshanesis*, *Amargasaurus cazaui*, *Kaatedocus siberi*, *Erketu*, *Diamantinasaurus*, *Bonitasaura*, and the Peirópolis axis A; Gomez et al., 2021; Tschopp & Mateus, 2012; Zhang et al., 2020), it is transversely wide in the Vila Ventura axis. In this respect, it resembles the condition present in *Galeamopus hayi* (Tschopp et al., 2015), although it is broader and bulge‐like in the Vila Ventura axis, approximating the breadth of the odontoid rather than forming a laminar flange or knob‐like tuberosity as in other sauropods. This feature may represent another potential autapomorphy of the Vila Ventura species.

The Vila Ventura axis exhibits a high degree of external and internal pneumatization, comparable to the condition observed in highly nested titanosaur clades (e.g., Zurriaguz & Cerda, 2017). Externally, large paired pneumatopores occur on both the ventral surface of the intercentrum and within the spinodiapophyseal fossae, as in *Bonitasaura*, *Pitekunsaurus*, and the Peirópolis axis A. This contrasts with *Saltasaurus*, *Diamantinasaurus*, the Peirópolis axis B, and the Nand axis, which lack large ventral pneumatopores in the intercentrum and, within the spinodiapophyseal fossae, pneumatopores are reduced to small perforations embedded into a pneumatocoel. CT‐scan data revealed pneumatic canals connecting to these pneumatopores, a condition not previously documented in saltasauroids. Internally, the presence of both circumferential and radial camellae with incipient internal bony plates is similar to the condition described in some diplodocids (e.g., OMNH 01094; Wedel, 2003), but is also documented in lognkosaurians (e.g., *Austroposeidon magnificus*; Bandeira et al., 2016), and in saltasaurines, including *Alamosaurus sanjuanensis*, *Ibirania*, and *Saltasaurus* (Aureliano, Nascimento, et al., 2021; Powell, 1992; Woodward & Lehman, 2009).

The morphology of the Vila Ventura axis closely resembles that observed in colossosaurians (Figure 7e–h), such as *Bonitasaura* and *Pitekunsaurus*, but particularly *Futalognkosaurus*. These anatomical similarities support the referral of the Vila Ventura axis (MPPC/GPRD 25‐013) to this clade, with probable affinities to Lognkosauria, pending further phylogenetic resolution or the discovery of more complete material. However, the absence of associated remains with the specimen, as well as atlantoaxial material associated with nominal titanosaur taxa from the Bauru Group (see above), precludes a conclusive taxonomic assessment of whether MPPC/GPRD 25‐013 belongs to a previously described species or represents a distinct, new taxon. As there is no anatomical overlap with other morphotypes from the same stratigraphic unit or locality, we refrain from erecting a new taxon at this stage.

### 5.2 Biomechanical and paleoecological implications

Sauropod axes exhibit broad morphological disparity, probably reflecting diverse biomechanic adaptations in the structure of the neck (Carabajal et al., 2014; Stevens, 2013) and, consequently, feeding behavior (Militello et al., 2026; Poropat et al., 2025, figure 6; Stevens & Parrish, 2005; Vidal et al., 2020). Although complete and articulated sauropod necks are exceptionally rare (Taylor, 2022), most of the available cervical series that are somewhat complete seem to exhibit a consistent pattern of morphological transitions along the column, likely reflecting biomechanical constraints near the craniovertebral and cervicodorsal boundaries (Fronimos et al., 2016). As observed in some titanosaur articulated series (e.g., MCT.R.1487, MAU‐Pv‐AC‐01, *Futalognkosaurus*, *Inawentu*; Calvo et al., 1997, 2007; Filippi et al., 2024; Powell, 2003), the longest cervical vertebrae are usually positioned near the middle of the neck (between 6th and 8th elements), whereas the anterior and posteriormost cervical elements, including atlas‐axis complex, are comparatively shorter. This pattern denotes that vertebrae adjacent to the cranial and trunk articulations are more compact, possibly for enhancing stability and potentially provide a structural reinforcement at these key functional anchors (Fronimos & Wilson, 2017).

Among Titanosauria, saltasauroids and closely related taxa (e.g., *Diamantinasaurus*, *Neuquensaurus*, *Saltasaurus*; Figure 7) possess an anteroposteriorly short axis centrum, with relatively broad and dorsoventrally tall neural arches, as also indicated by their low CL:TH and aEI values (Table 2). A similar pattern is observed in some mamenchisaurids (e.g., *Xinjiangtitan*), dicraeosaurids (e.g., *Amargasaurus*), early macronarians (e.g., *Camarasaurus*) and non‐titanosaur somphospondylians, such as *Euhelopus* (Figure 7a). In these taxa, the Axis to Cv‐III ratio is also lower than in forms with an elongated axis (Table 3), indicating that the shortening of this element could be part of a broader pattern of cervical compaction. This configuration may suggest a restricted range of motion between the skull and neck, in which compaction of the anterior cervical segment could have favored stability over extensive cranial mobility.

**TABLE 3:** Axis‐cervical III anteroposterior centrum length ratio (decreasing order) in selected sauropod taxa.

Taxon	Axis CL^a^	Cv‐III CL^a^	Ratio
**Basal Sauropoda**			
*Spinophorosaurus nigerensis*	156	195	0.80
**Eusauropoda**			
*Patagosaurus fariasi*	158**	155**	1.02
*Jinchuanloong niedu*	80	80	1.00
*Bagualia alba*	110	180	0.61
**Mamenchisauridae**			
*Omeisaurus tianfuensis*	185	241	0.77
*Mamenchisaurus hochuanensis*	160	215	0.74
*Mamenchisaurus youngi*	124	170	0.73
*Mamenchisaurus sinocanadorum*	187	294	0.64
*Xinjiangtitan shanshanesis*	165	260	0.63
*Qijianglong guokr*	143	256	0.56
**Turiasauria**			
*Moabosaurus utahensis*	148	136	1.09
**Dicraeosauridae**			
*Dicraeosaurus hansemanni*	160	185	0.86
*Smitanosaurus agilis*	61	83	0.73
*Amargasaurus cazaui*	119	187	0.64
**Diplodocidae**			
*Galeamopus hayi*	200**	240	0.83
*Apatosaurus louisae*	190	280	0.68
*Diplodocus carnegii*	163	243	0.67
*Kaatedocus siberi*	88**	134	0.66
**Camarasauridae**			
*Camarasaurus lewisi*	185	193	0.96
*Camarasaurus supremus*	235	265	0.89
**Brachiosauridae**			
*Giraffatitan brancai*	203	257	0.79
*Abydosaurus mcintoshi*	180	232	0.78
**Euhelopodidae**			
*Yunmenglong ruyangensis*	250	360	0.69
*Euhelopus zdanskyi*	94	146	0.64
*Erketu ellisoni*	160	268	0.60
**Titanosauria**			
MCT.R.1487 (Series A)	103**	122**	0.84
*Futalognkosaurus dukei*	245	340	0.72
*Tapuiasaurus macedoi*	163	231**	0.71
*Inawentu oslatus*	144	210*	0.69
*Neuquensaurus australis*	50	75	0.67
*Diamantinasaurus matildae*	113	171	0.66
*Alamosaurus sanjuanensis*	135**	223**	0.61
*Saltasaurus loricatus*	55	138	0.40

*Note*: To eliminate potential ontogenetic bias, all measurements (in mm) were taken exclusively from elements belonging to a single individual. An asterisk (*) marks the measurements based on preserved portions, while a double asterisk (**) corresponds to measurements taken from photographs (ImageJ).

Abbreviations: CL, centrum length; Cv, cervical vertebra.

^a^ Measures taken from the ventral surface (excluding the odontoid or articular condyles).

In contrast, colossosaurs comparatively exhibit more elongated axis centra (Table 2), with large‐bodied forms (lognkosaurians) having proportionally tall neural spines (e.g., *Futalognkosaurus*; Figure 7e), whereas smaller‐bodied taxa such as rinconsaurians (e.g., *Pitekunsaurus*) tend to have dorsoventrally lower spines (Figure 7h). This variability suggests a size‐dependent scaling of some structures in the axis (e.g., odontoid, anterior spinal projection, neural spine development), potentially influencing muscle attachment sites or reflecting differences in the orientation of the skull and neck. Colossosaurs present high aEI (>2.4), CL:TH (>1.0), and Axis to Cv‐III ratio (>0.7) values, suggesting that the elongation of the axis centra was a defining morphological feature in this group and potentially tied to their characteristic general neck structure. Indeed, this clade displays unique cervical features within titanosaurs. Lognkosaurians exhibit taller and expanded postaxial cervical spines with laminae forming a fan‐like profile, while rinconsaurians and aeolosaurines have cervical spines bearing dorsolateral bulges that give an inflated aspect to the neural spine (Bandeira et al., 2016; Gallina, 2011; González Riga & Ortiz David, 2014; Pérez Moreno et al., 2021).

In sum, these patterns indicate that the elongation of the axis may serve as a proxy for overall neck proportions. If so, taxa with short axes should be expected to have relatively shorter necks compared to similar sized forms with elongate axes. Axis to Cv‐III ratio provides additional support for this interpretation (Table 3). Forms with longer axes tend to exhibit gradual increases in centrum length along the cervical series (e.g., *Futalognkosaurus*, MCT.R.1487) compared to taxa with short axes (e.g., *Saltasaurus*).

Nevertheless, both the elongation of cervical centra and the total number of cervical vertebrae vary considerably across Sauropoda (Taylor & Wedel, 2013). Taxa with proportionally short axes (CL:TH = 0.7–0.9; Table 2) may have high cervical counts, such as *Mamenchisaurus hochuanensis* (19 elements; Yang & Zhao, 1972) and *Euhelopus* (17 elements; Wilson & Upchurch, 2009). By contrast, most titanosaurs possess a relatively elongate axis (CL:TH = 0.9–1.3) but exhibit a broad range of cervical vertebrae counts: *Inawentu* (12 elements; Filippi et al., 2024), MCT.R.1487 and MAU‐Pv‐AC‐01 (13 elements; Powell, 1987; pers. obs.), *Futalognkosaurus* and *Abditosaurus kuehnei* (14 elements; Calvo et al., 2007; Vila et al., 2022), AODF 888 (15 elements; Poropat et al., 2025), and *Rapetosaurus* (16 elements; Curry Rogers, 2009). Saltasauroids deviate from the general titanosaur pattern, although their cervical count remains unknown, presenting a proportionally very short axis (CL:TH <0.5). A more comprehensive analysis of taxa with complete, articulated or associated cervical series could provide further insights into the correlation between axis elongation and overall neck length, in regard to both cervical count and centra elongation within the cervical series.

The possible relationship between axis morphology and overall neck length may point to differences in titanosaur feeding strategies, possibly reflecting adaptive responses to new ecological conditions during the Late Cretaceous (Bates et al., 2016; Chiarenza, 2024). After the Cenomanian–Turonian interval, which marked the extinction of the last rebbachisaurids and brachiosaurids, titanosaurs progressively expanded into newly available ecological niches and diversified both morphologically and functionally (Calvo et al., 2006; Filippi et al., 2024; Otero et al., 2021). This adaptive radiation likely involved modifications in neck structure and feeding mechanics that enabled titanosaurs to exploit a broader range of resources within Late Cretaceous terrestrial ecosystems.

### 5.3 Phylogenetic significance

Axis vertebrae remain an underexplored source of phylogenetic information in Sauropoda, despite their anatomical complexity and structural role within the cervical series. Among Titanosauria, where anatomical overlaps are often affected by the fragmentary nature of their fossil record, the axis can preserve a combination of discrete and meristic traits that are valuable to assess its systematic affinities, particularly when this element is preserved in isolation.

However, in most current phylogenetic datasets, axis‐related characters represent only a small fraction of the cervical character set, reflecting both the rarity of preserved atlantoaxial material and the challenges in defining discrete, scorable states for this highly modified vertebra. Characters of the axis vertebra are comparatively scarce in the widely used sauropod phylogenetic datasets (e.g., Carballido & Sander, 2014; D'Emic, 2012; Mannion et al., 2013; Tschopp et al., 2015; Whitlock, 2011; Wilson, 2002), representing <0.5% of the cervical‐related characters in these matrices, even in their most updated versions (e.g., Bellardini et al., 2022; D'Emic et al., 2016; Filippi et al., 2024; Gallina et al., 2021; Mannion et al., 2019; Pérez Moreno et al., 2022; Poropat et al., 2021, 2023; Whitlock & Wilson Mantilla, 2021). This scarcity stands in contrast to the rich morphological diversity present in sauropod axes, constraining the assessment of morphological variation along the cervical series of sauropod.

The Vila Ventura axis exemplifies the potential of axis elements to provide novel and phylogenetically informative characters. Through the analysis of the Vila Ventura axis and comparative examination of other sauropod axes, we compiled 20 characters (Table 4), including both features previously proposed in the literature (chars 435, 436, 440, 442, 446, 450, 451, 453) and novel ones identified in this study (chars 437–439, 441, 443–445, 447–449, 452, 454). Axis characters encompass general proportions (chars 435, 453), centrum proportions (chars 436–437) and morphology (chars 438–442), laminar configuration (chars 445–446, 449–450), external pneumatic traits (chars 439, 447–448), development of transverse processes (chars 443, 454), prezygapophysis (char 444), and neural spine (chars 451–452).

**TABLE 4:** Proposed coding of morphological variation in sauropod axes.

Char	Statement	Proposed states	Parsimony model	Source
435	Axis, general shape and proportions (centrum length: total height)	0 Centrum length is less than twice its total height (CL:TH <0.9) 1 Centrum length exceeds 2.5 times its total height (CL:TH ≥0.9 or greater)	Unordered	Modified from D'Emic, 2012: char 20; see Table 2
436	Axis, average Elongation Index (aEI sensu Chure et al., 2010)	0 aEI ≥3.0 or greater 1 aEI ≥2.0 and <3.0 2 aEI <2.0	Unordered	Modified from Mannion et al., 2019: char 322; see Table 2
437	Atlantal pleurocentrum (odontoid), anteroposterior length relative to axial intercentrum	0 Less than 50% of the anteroposterior length of the axial intercentrum 1 Equal to or >50% of the anteroposterior length of the axial intercentrum	Unordered	This study
438	Axial intercentrum, ventral surface, longitudinal ridge	0 Absent 1 Present as a developed keel or rim	Unordered	This study
439	Axial intercentrum, excavations on the ventral surface	0 Absent 1 Incipient, as pneumatopores 2 Present as broad fossae	Unordered	This study
440	Axial pleurocentrum, midline ventral keel or rim	0 Absent 1 Present	Unordered	Mannion et al., 2013: char 117
441	Axial pleurocentrum, midline ventral keel or rim, position	0 Restricted on the anteroventral surface 1 Restricted on the posteroventral surface 2 Extending along the entire ventral surface	Unordered	This study
442	Axial pleurocentrum, excavations on ventrolateral edges, posterior and adjacent to parapophyses	0 Absent 1 Present	Unordered	Tschopp et al., 2015: char 150
443	Axial parapophyses, articular facet shape and position	0 Round and projected laterally, mostly in the intercentrum 1 Elliptical and projected posterolaterally, extending onto pleurocentrum	Unordered	This study
444	Axial neural arch, development of prezygapophyses	0 Restricted to articular facets or slightly projected anteriorly, <50% of odontoid process anteroposterior length 1 Well‐developed, projecting anteriorly at least 50% of odontoid process anteroposterior length	Unordered	This study
445	Axial neural arch, development of the prezygodiapophyseal (PRDL) and anterior centrodiapophyseal laminae (ACDL)	0 Undifferentiated, forming a single, ribbon‐like lamina (PRDL+ACDL) 1 Both arise as distinct laminae	Unordered	This study
446	Axial neural arch, prespinal lamina (PRSL) with projected, knob‐like process at its anterior end	0 Absent or incipient, being less developed than the anteroposterior length of prezygapophyses 1 Present and well‐developed, extending as far as the prezygapophyses	Unordered	Modified from Tschopp et al., 2015: char 151
447	Axial neural arch, pneumatocoel in the spinodiapophyseal fossae (SDF)	0 Absent 1 Present	Unordered	This study
448	Axial neural arch, large pneumatopores in the spinodiapophyseal fossae (SDF)	0 Absent 1 Present	Unordered	This study
449	Axial neural arch, posterolateral expansion of the spinoprezygapophyseal laminae (SPRL), forming pointed prominences	0 Absent 1 Present	Unordered	This study
450	Axial neural arch, postspinal lamina (POSL)	0 Absent 1 Present	Unordered	Tschopp et al., 2015: char 152
451	Axial neural spine, anteroposterior development	0 Restricted anterior to postzygapophyseal facets 1 Terminates in front of or at posterior border of centrum 2 Projects beyond the posterior border of centrum	Ordered	Tschopp et al., 2015: char 153
452	Axial neural spine, transverse development (relative to cotyle width)	0 Equal to or less than the transverse width of the cotyle 1 Greater (at least 1.5 times) the transverse width of the cotyle	Unordered	This study
453	Axis to cervical vertebra III, anteroposterior centrum length ratio (excluding the odontoid and articular condyle)	0 Equivalent centrum lengths: Axis:Cv‐III ratio ≥0.9 or greater 1 Slight to moderate centrum reduction: Axis:Cv‐III ratio ≥0.7 and <0.9 2 Considerable centrum reduction: Axis:Cv‐III ratio <0.7	Ordered	Modified from Tschopp et al., 2015: char 155; see Table 3
454	Axial ribs, proximal articulation	0 Bicapitate, capitulum and tuberculum present on ribs, diapophyses in the neural arch projects ventrally 1 Unicapitate, only the capitulum is present on ribs, diapophyses in the neural arch are wing‐shaped and projects posteriorly	Unordered	This study

Several of these characters show clear distributional patterns within Titanosauria. Colossosaurian taxa predominantly display an elongate axis (chars 435[1], 436[0&1], 453[1]), elliptical parapophyses extending onto the pleurocentrum (char 443[1]), associated ribs with unicapitate proximal articulations (char 454[1]), well‐developed anterior spinal projection (char 446[1]), and external pneumaticity evidenced by simpler, large pneumatopores (chars 439[2], 447[0], 448[1]). In contrast, saltasauroids more commonly exhibit a short axis (chars 435[0], 436[2], 453[2]), rounded and laterally projecting parapophyses (char 443[0]), associated ribs with bicapitate proximal articulations (char 454[0]), reduced or absent anterior spinal projections (char 446[0]), and external pneumaticity with large fossae or pneumatopores in the intercentrum absent (char 439[0]) and multiple pneumatopores in the spinodiapophyseal fossa being small and embedded into pneumatocoels (chars 447[1], 448[0]).

The combination of features in the Vila Ventura axis aligns more closely with the condition in colossosaurs, particularly lognkosaurians, than with that in saltasauroids. These include an elongate centrum (chars 435[1], 436[1]), paired ventral fossae in the intercentrum (char 439[2]), ventrolateral excavations on the pleurocentrum (char 442[1]), presence of an anterior spinal projection extending as far as prezygapophyses (char 446[1]), and large spinodiapophyseal pneumatopores (char 448[1]). Several of these features have no equivalent characters in existing matrices, so their inclusion could improve resolution both at higher taxonomic levels (e.g., Colossosauria vs. Saltasauroidea) and in the distinction of specific morphotypes within these clades (e.g., Lognkosauria vs. Rinconsauria).

Despite being an exploratory approach, the inclusion of these characters in a phylogenetic analysis, together with the scoring of the Vila Ventura specimen, allowed the evaluation of whether axis morphology carries phylogenetic signals. The iteration under equal weighting resulted in 261 most parsimonious trees (MPTs) with 1925 steps (CI = 0.308; RI = 0.686). The strict consensus tree exhibits polytomies within Somphospondyli and Titanosauriformes, although Lognkosauria, Saltasauroidea, and the clade ((*Patagotitan* + *Argentinosaurus*) + (*Kaijutitan* + *Bonitasaura*)) were still recovered (Figure 9). The Vila Ventura axis was nested in a broad polytomy with other somphospondylians, with five autapomorphic characters. Despite the limited resolution, several of the newly proposed axial characters were recovered as local autapomorphies for multiple OTUs, while others were retrieved as synapomorphic at both higher and lower phylogenetic levels (Table 5). These results indicate that axis morphology contains informative phylogenetic signal even when represented by an isolated element. The implied weighting iteration (*K* = 9) resulted in five MPTs with a length of 94.21404 (CI = 0.306, RI = 0.682). Node resolution was improved compared to the equal weighting analysis (Figure 10), although polytomies still persisted in Brachiosauridae and Lognkosauria. The Vila Ventura axis was recovered within Colossosauria but did not occupy a stable position inside Lognkosauria. Instead, MPPC/GPRD 25‐013 formed an unresolved cluster with *Futalognkosaurus*, *Mendozasaurus*, and the cervical series BIBE 45854 referred to *Alamosaurus* (Tykoski & Fiorillo, 2017). Likewise, four characters were recovered as autapomorphies of the Vila Ventura axis. Additionally, several of the newly proposed axial characters were retrieved as autapomorphic for individual OTUs and as synapomorphic for specific clades (Table 5), supporting the potential diagnostic utility of the axis morphology in Sauropoda.

**TABLE 5:** Apomorphies recovered for axis characters under Equal‐ and Implied‐weighting analyses.

OTU	Equal weighted analysis	Implied weighted analysis
**Autapomorphies**		
*Omeisaurus* spp.	435 (1 → 0), 436 (1 → 0)	435 (1 → 0), 436 (1 → 0)
*Mamenchisaurus* spp.	445 (0 → 1)	–
*Jobaria tiguidensis*	450 (1 → 2)	–
*Suuwassea emiliae*	450 (1 → 0)	450 (1 → 0)
*Amargasaurus cazaui*	435 (1 → 2), 439 (1 → 0)	435 (1 → 2), 439 (1 → 0)
*Bellusaurus sui*	439 (1 → 0)	439 (1 → 0)
*Camarasaurus* spp.	449 (0 → 1), 452 (1 → 0)	445 (1 → 0), 449 (0 → 1), 452 (1/2 → 0)
*Euhelopus zdanskyi*	436 (1 → 0), 439 (1 → 0)	–
*Erketu ellisoni*	435 (1 → 0), 438 (0 → 1), 443 (0 → 1)	–
*Tapuiasaurus macedoi*	435 (1 → 0), 438 (0 → 1), 442 (1 → 0), 443 (0 → 1), 445 (1 → 0), 452 (2 → 1)	435 (1 → 0), 438 (0 → 1), 442 (1 → 0)
*Pitekunsaurus macayai*	439 (1 → 0), 448 (0 → 1)	–
*Diamantinasaurus matildae*	435 (1 → 2), 440 (0 → 2), 442 (1 → 0), 445 (1 → 0), 446 (0 → 1), 450 (0 → 1)	–
*Neuquensaurus australis*	440 (2 → 1)	440 (2 → 1)
Vila Ventura axis	437 (0 → 1), 438 (0 → 2), 443 (0 → 1), 444 (0 → 1), 448 (0 → 1)	437 (0 → 1), 438 (0 → 2), 447 (0 → 1), 448 (0 → 1)
**Synapomorphies**		
	Equal weighted analysis	Implied weighted analysis
Node 117: Mamenchisauridae	450 (1 → 0)	–
Node 121: *Mamenchisaurus* + Neosauropoda	–	439 (0 → 1), 445 (0 → 1)
Node 122: *Amargasaurus* + Dicraeosaurinae	449 (0 → 1), 450 (1 → 2)	–
Node 128: *Amargasaurus* + Dicraeosaurinae	–	449 (0 → 1), 450 (1 → 2)
Node 137: Macronaria	442 (0 → 1)	–
Node 140: Somphospondyli	445 (0 → 1), 452 (1 → 2)	–
Node 172: *Tapuiasaurus* + Lognkosauria	–	443 (0 → 1)
Node 173: Colossosauria	–	453 (0 → 1)
Node 175: Lognkosauria	–	444 (0 → 1)

*Note*: Character numbering follows that incorporated into the matrix (see Table 4). The full topology and associated data are available in the Data S1.

Integrating axis morphology into a phylogenetic framework is also relevant for interpreting broader evolutionary trends in the neck structure of sauropods. For example, the development of prezygapophyses and odontoid in the axis implies directly on the atlantal morphology and their articulation with craniodental skeleton. Likewise, several features observed in morphotypes with an elongated axis, exemplified by the Vila Ventura specimen, closely resemble those of the third cervical vertebra in the morphotypes with a shorter axis, such as *Euhelopus* (Figure 8). This suggests that characters typically associated with postaxial cervical vertebrae may also occur in taxa with elongated axes, thus suggesting that these features have a functional significance and can vary serially.

**FIGURE 8:**
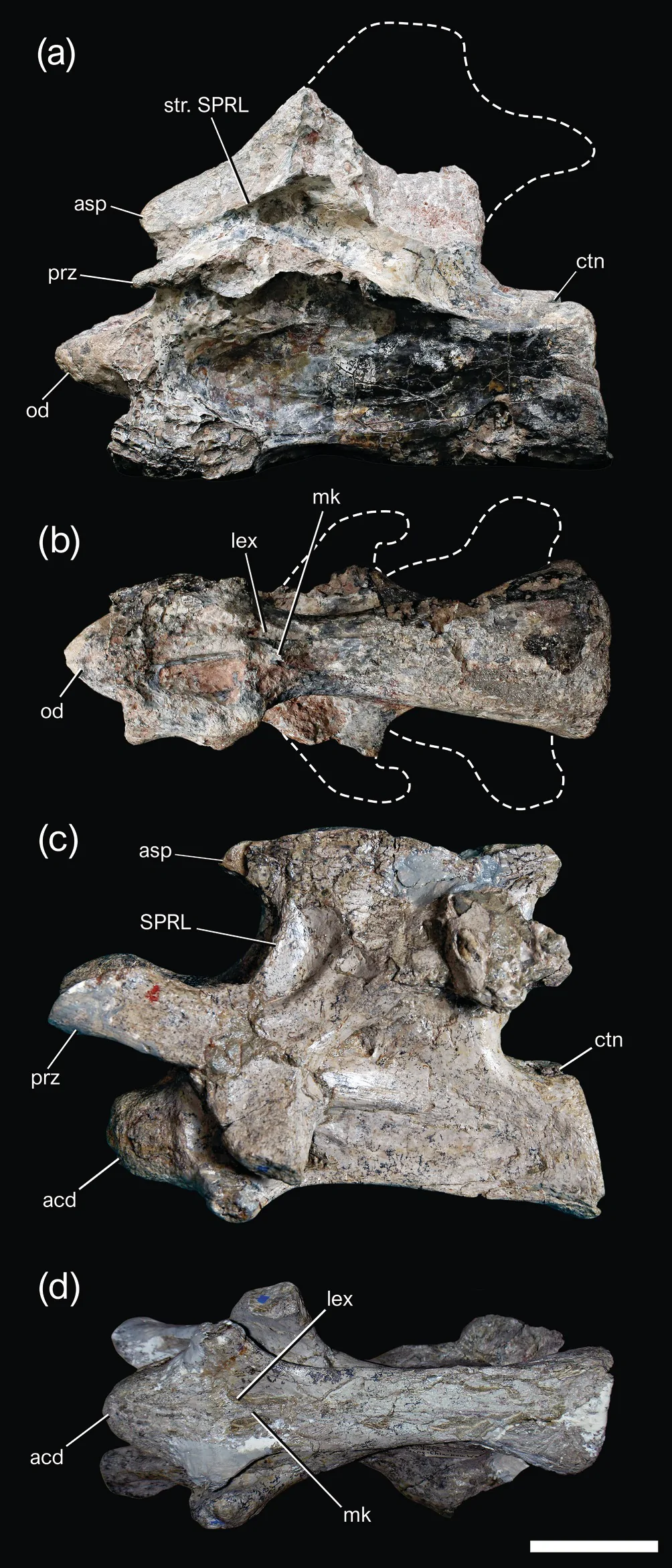
Comparison (to scale) between the Vila Ventura axis (MPPC/GPRD 25‐013), an elongated axis morphotype (a–b), and the third cervical vertebra of *Euhelopus zdanskyi* (PMU.R233), an example of a morphotype with a short axis (c–d), depicting some shared structures (e.g., asp, ctn, lex, mk). Abbreviations: acd, articular condyle; asp, anterior spinal projection; ctn, cotylar notch; lex, lateral excavation; mk, midline ventral keel; od, odontoid; prz, prezygapophysis; SPRL, spinoprezygapophyseal lamina; str., stranded. Scale bar equals 5 cm.

**FIGURE 9:**
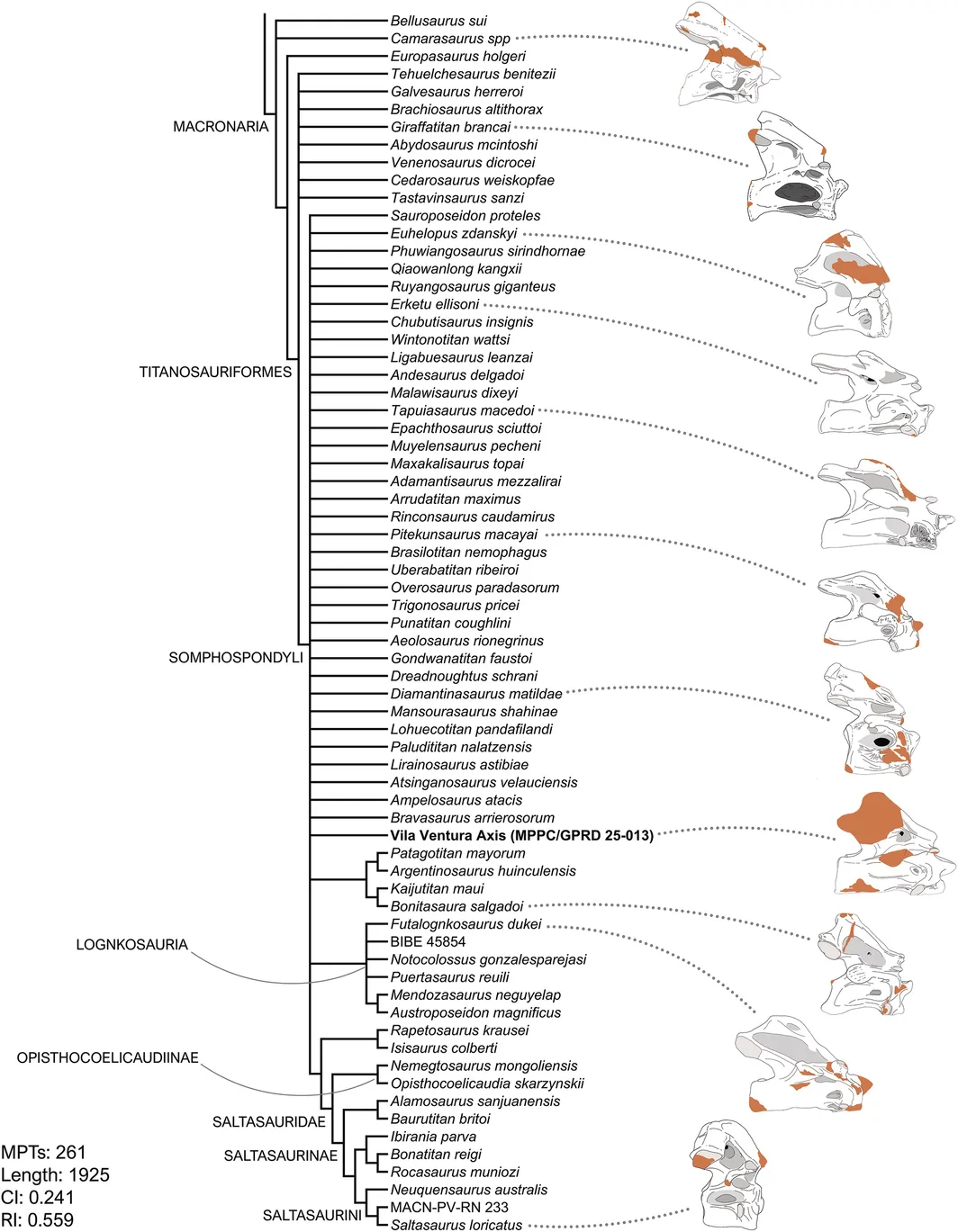
Strict consensus of 261 most parsimonious trees (MPTs) obtained under equal‐weighting iteration, with reconstructions of the axis morphotypes. Gray tones indicate fossae and articular surfaces, whereas orange tones highlight reconstructed damaged areas. Abbreviations: CI, Consistency Index; RI, Retention Index.

**FIGURE 10:**
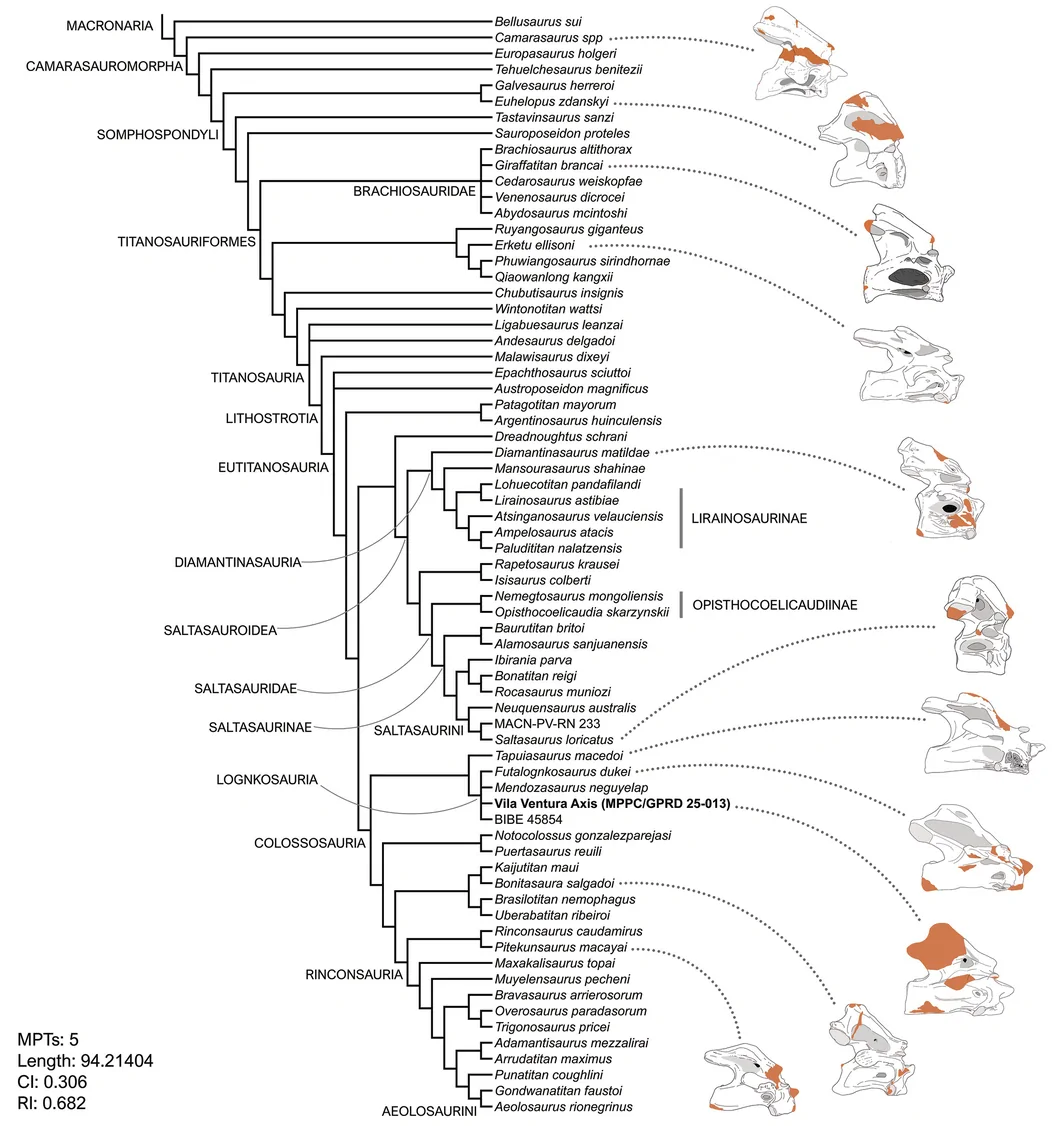
Strict consensus of five most parsimonious trees (MPTs) obtained under implied‐weighting iteration (K = 9), with reconstructions of the axis morphotypes. Gray tones indicate fossae and articular surfaces, whereas orange tones highlight reconstructed damaged areas. Abbreviations: CI, Consistency Index; RI, Retention Index.

## 6 CONCLUDING REMARKS

Axis vertebrae are relatively rare in the sauropod fossil record, likely due to their structure and taphonomic biases that affect sauropod skeleton preservation. This scarcity constrains direct comparative analyses of this vertebral element across several taxa. In this context, the Vila Ventura axis offers valuable anatomical information on that undersampled region of the sauropod postcranial skeleton. Although the absence of associated skeletal elements precludes a precise taxonomic assignment, morphological evidence and phylogenetic analyses support referral of the Vila Ventura axis (MPPC/GPRD 25‐013) to Colossosauria, with affinities to Lognkosauria. Based on linear measurements and proportions, the Vila Ventura axis belonged to an animal with overall dimensions comparable to those of *Futalognkosaurus*, indicating the presence of giant lognkosaurians in the Bauru Group prior to the Campanian.

The elongation of the axis vertebra potentially holds implications for neck structure and skull orientation in Sauropoda and may provide new perspectives into ecomorphological adaptations of the group. The centrum length to total height ratio (CL:TH), average Elongation Index (aEI), and the relative proportion of the axis to the third cervical vertebra (Axis to Cv‐III ratio) can offer valuable insights into the variation in vertebral proportions and their potential morphofunctional significance. This represents a promising framework that should be explored using articulated series and quantitative range‐of‐motion approaches.

The morphology of the axis vertebra has received little attention in currently used phylogenetic datasets, compared to other axial modules of the sauropod skeleton. Yet, diagnostic characters in the axis, as we recognized, provide a robust framework for taxonomic assignments and the assessment of systematic affinities at both higher and lower phylogenetic levels, particularly when associated skeletal elements are absent. Our new characters, together with the coding of these characters as observed on the identified morphotypes, suggest that it is possible to distinguish anatomical patterns between the two major lineages in Titanosauria (i.e., Colossosauria and Saltasauroidea), clades that were previously diagnosed primarily on the basis of their appendicular anatomy. Cladistic analyses recovered axis morphology as phylogenetically informative, revealing autapomorphic traits in specific taxa and diagnostic characters at both higher and lower phylogenetic levels across different sauropod clades.

The analysis of the Vila Ventura axis reinforces that atlantoaxial material, even when isolated, can yield an informative set of characters with diagnostic value for titanosaur systematics, highlighting its importance in recognizing distinct morphotypes. Future discoveries of more complete specimens will be crucial for further clarifying the evolutionary pathways that shaped the neck morphology in Sauropoda.

## AUTHOR CONTRIBUTIONS

**Bruno A. Navarro:** Conceptualization; investigation; funding acquisition; writing – original draft; methodology; validation; writing – review and editing; formal analysis; project administration. **Jeffrey A. Wilson Mantilla:** Writing – original draft; methodology; validation; visualization; writing – review and editing; supervision; conceptualization. **Jhonata H. A. Martins:** Conceptualization; validation; visualization; writing – review and editing; writing – original draft. **Fabiano V. Iori:** Validation; visualization; writing – review and editing; data curation; writing – original draft. **Leonardo S. Paschoa:** Visualization; validation; data curation. **Alberto B. Carvalho:** Writing – original draft; validation; visualization; software; formal analysis; methodology. **Joseli M. Piranha:** Writing – review and editing; supervision; validation; visualization. **Hussam Zaher:** Supervision; writing – review and editing; visualization; validation; resources; project administration.

## FUNDING INFORMATION

São Paulo Research Foundation (FAPESP) scholarship 2023/11098‐3.

## CONFLICT OF INTEREST STATEMENT

The authors declare no competing interests.

## Supporting information


**DATA S1.** Supporting information.

**DATA S2.** Supporting information.

## References

[ar70118-bib-0001] Apesteguía, S. (2004). *Bonitasaura salgadoi* gen. et sp. nov.: A beaked sauropod from the Late Cretaceous of Patagonia. Naturwissenschaften, 91(10), 493–497. 10.1007/s00114-004-0560-6 15729763

[ar70118-bib-0002] Arai, M. , & Dias‐Brito, D. (2023). Supersequência Bauru (Cretáceo da Bacia do Paraná): Revisão estratigráfica com base em dados paleontológicos recentes. Derbyana, 44, e800. 10.14295/derb.v44.800

[ar70118-bib-0003] Arid, F. M. , & Vizotto, L. D. (1963). Sobre vertebrados fósseis no município de Ibirá, São Paulo. Ciência e Cultura, 15(3), 181–182.

[ar70118-bib-0004] Arid, F. M. , & Vizotto, L. D. (1971). *Antarctosaurus brasiliensis*, um novo saurópode do Cretáceo Superior do sul do Brasil. Anais, 25(1971), 297–305.

[ar70118-bib-0005] Arid, F. M. , Vizotto, L. D. , & Landim, P. M. B. (1962). A ocorrência de um jazigo fossilífero nos arredores de São José do Rio Preto. Ciência e Cultura, 14(3), 172.

[ar70118-bib-0006] Aureliano, T. , Almeida, W. , Fernandes, M. A. , & Ghilardi, A. M. (2026). Several occurrences of osteomyelitis in dinosaurs from a site in the Bauru Group, Cretaceous of Southeast Brazil. The Anatomical Record, 309(3), 642–648. 10.1002/ar.70003 PMC12882049 40448361

[ar70118-bib-0007] Aureliano, T. , Ghilardi, A. M. , Navarro, B. A. , Fernandes, M. A. , Ricardi‐Branco, F. , & Wedel, M. J. (2021). Exquisite air sac histological traces in a hyperpneumatized nanoid sauropod dinosaur from South America. Scientific Reports, 11(1), 24207. 10.1038/s41598-021-03689-8 PMC8683417 34921226

[ar70118-bib-0008] Aureliano, T. , Nascimento, C. S. I. , Fernandes, M. A. , Ricardi‐Branco, F. , & Ghilardi, A. M. (2021). Blood parasites and acute osteomyelitis in a non‐avian dinosaur (Sauropoda, Titanosauria) from the Upper Cretaceous Adamantina Formation, Bauru Basin, Southeast Brazil. Cretaceous Research, 118, 104672. 10.1016/j.cretres.2020.104672

[ar70118-bib-0009] Bandeira, K. L. N. , Machado, E. B. , Campos, D. A. , & Kellner, A. W. A. (2019). New titanosaur (Sauropoda, Dinosauria) records from the Morro do Cambambe unit (Upper Cretaceous), Mato Grosso State, Brazil. Cretaceous Research, 103, 104155. 10.1016/j.cretres.2019.06.001

[ar70118-bib-0010] Bandeira, K. L. N. , Simbras, F. M. , Machado, E. B. , Campos, D. d. A. , Oliveira, G. , & Kellner, A. W. A. (2016). A new giant Titanosauria (Dinosauria: Sauropoda) from the Late Cretaceous Bauru Group, Brazil. PLoS One, 11(10), e0163373. 10.1371/journal.pone.0163373 PMC5051738 27706250

[ar70118-bib-0011] Barbosa, F. H. S. , Marinho, T. S. , Iori, F. V. , & Paschoa, L. S. (2019). A case of infection in an Aeolosaurini (Sauropoda) dinosaur from the Upper Cretaceous of São Paulo, southeastern Brazil, and the impact on its life. Cretaceous Research, 96, 1–5. 10.1016/j.cretres.2018.12.004

[ar70118-bib-0012] Basilici, G. , & Sgarbi, G. N. (2012). A Sub‐Bacia Bauru: Um sistema continental entre deserto e cerrado. In Y. Hasui , F. F. M. de Almeida , C. D. R. Carneiro , & A. Bartorelli (Eds.), Geologia do Brasil (pp. 520–543). Beca.

[ar70118-bib-0013] Bates, K. T. , Mannion, P. D. , Falkingham, P. L. , Brusatte, S. L. , Hutchinson, J. R. , Otero, A. , Sellers, W. I. , Sullivan, C. , Stevens, K. A. , & Allen, V. (2016). Temporal and phylogenetic evolution of the sauropod dinosaur body plan. Royal Society Open Science, 3(3), 150636. 10.1098/rsos.150636 PMC4821263 27069652

[ar70118-bib-0014] Batezelli, A. (2010). Arcabouço tectono‐estratigráfico e evolução das Bacias Caiuá e Bauru no Sudeste brasileiro. Revista Brasileira de Geociencias, 40, 265–285. 10.25249/0375-7536.2010402265285

[ar70118-bib-0015] Batezelli, A. (2015). Continental systems tracts of the Brazilian Cretaceous Bauru Basin and their relationship with the tectonic and climatic evolution of South America. Basin Research, 29(S1), 1–25. 10.1111/bre.12128

[ar70118-bib-0016] Bellardini, F. , Coria, R. A. , Pino, D. , Windholz, G. , Baiano, M. , & Martinelli, A. G. (2022). Osteology and phylogenetic relationships of *Ligabuesaurus leanzai* (Dinosauria: Sauropoda) from the Early Cretaceous of the Neuquén Basin, Patagonia, Argentina. Zoological Journal of the Linnean Society, 196(4), 1333–1393. 10.1093/zoolinnean/zlac003

[ar70118-bib-0017] Bonaparte, J. F. , & Coria, R. A. (1993). Un nuevo y gigantesco sauropodo titanosaurio de la Formación Río Limay (Albiano‐Cenomaniano) de la Provincia del Neuquén, Argentina. Ameghiniana, 30(3), 271–282.

[ar70118-bib-0018] Calvo, J. O. , Coria, R. A. , & Salgado, L. (1997). Uno de los más completos Titanosauridae (Dinosauria—Sauropoda) registrados en el mundo. Ameghiniana, 34, 534.

[ar70118-bib-0019] Calvo, J. O. , Gandossi, P. , & Porfiri, J. (2006). Dinosaur faunal replacement during Cenomanian times in Patagonia, Argentina. Ninth International Symposium on Mesozoic Terrestrial Ecosystems and Biota, Manchester.

[ar70118-bib-0020] Calvo, J. O. , Porfiri, J. D. , González Riga, B. J. , & Kellner, A. W. A. (2007). Anatomy of *Futalognkosaurus dukei* Calvo, Porfiri, González Riga & Kellner, 2007 (Dinosauria, Titanosauridae) from the Neuquén group (Late Cretaceous), Patagonia, Argentina. Arquivos Do Museu Nacional, Rio de Janeiro, 65(4), 511–526.

[ar70118-bib-0021] Campos, D. A. , & Kellner, A. W. A. (1999). On some sauropod (Titanosauridae) pelves from the continental Cretaceous of Brazil. National Science Museum Monographs, 15, 143–166.

[ar70118-bib-0022] Campos, D. A. , Kellner, A. W. A. , Bertini, R. J. , & Santucci, R. M. (2005). On a titanosaurid (Dinosauria, Sauropoda) vertebral column from the Bauru Group, Late Cretaceous of Brazil. Arquivos Do Museu Nacional, Rio de Janeiro, 63(3), 565–593.

[ar70118-bib-0023] Carabajal, A. P. , Carballido, J. L. , & Currie, P. J. (2014). Braincase, neuroanatomy, and neck posture of *Amargasaurus cazaui* (Sauropoda, Dicraeosauridae) and its implications for understanding head posture in sauropods. Journal of Vertebrate Paleontology, 34(4), 870–882.

[ar70118-bib-0024] Carballido, J. L. , Otero, A. , Mannion, P. D. , Salgado, L. , & Pérez Moreno, A. (2022). Titanosauria: A critical reappraisal of its systematics and the relevance of the South American record. In South American Sauropodomorph dinosaurs: Record, diversity and evolution (pp. 269–298). Springer International Publishing. 10.1007/978-3-030-95959-3_8

[ar70118-bib-0025] Carballido, J. L. , & Sander, P. M. (2014). Postcranial axial skeleton of *Europasaurus holgeri* (Dinosauria, Sauropoda) from the Upper Jurassic of Germany: Implications for sauropod ontogeny and phylogenetic relationships of basal Macronaria. Journal of Systematic Palaeontology, 12(3), 335–387. 10.1080/14772019.2013.764935

[ar70118-bib-0026] Carvalho, I. S. , Salgado, L. , Lindoso, R. M. , de Araújo‐Júnior, H. I. , Nogueira, F. C. C. , & Soares, J. A. (2017). A new basal titanosaur (Dinosauria, Sauropoda) from the Lower Cretaceous of Brazil. Journal of South American Earth Sciences, 75, 74–84. 10.1016/j.jsames.2017.01.010

[ar70118-bib-0027] Cashmore, D. , Mannion, P. D. , Upchurch, P. , & Butler, R. (2020). Ten more years of discovery: Revisiting the quality of the sauropodomorph dinosaur fossil record. Palaeontology, 63, 1–28. 10.1111/pala.12496

[ar70118-bib-0028] Castro, M. C. , Goin, F. J. , Ortiz‐Jaureguizar, E. , Vieytes, E. C. , Tsukui, K. , Ramezani, J. , Batezelli, A. , Marsola, J. C. A. , & Langer, M. C. (2018). A Late Cretaceous mammal from Brazil and the first radioisotopic age for the Bauru Group. Royal Society Open Science, 5(5), 180482. 10.1098/rsos.180482 PMC5990825 29892465

[ar70118-bib-0029] Cerda, I. A. , Paulina Carabajal, A. , Salgado, L. , Coria, R. A. , Reguero, M. A. , Tambussi, C. P. , & Moly, J. J. (2012). The first record of a sauropod dinosaur from Antarctica. Naturwissenschaften, 99(1), 83–87. 10.1007/s00114-011-0869-x 22173579

[ar70118-bib-0030] Chiarenza, A. A. (2024). The macroecology of Mesozoic dinosaurs. Biology Letters, 20(11), 20240392. 10.1098/rsbl.2024.0392 PMC11558851 39535111

[ar70118-bib-0142] Chure, D. , Britt, B. B. , Whitlock, J. A. , & Wilson, J. A. (2010). First complete sauropod dinosaur skull from the Cretaceous of the Americas and the evolution of sauropod dentition. Naturwissenschaften, 97(4), 379–391. 10.1007/s00114-010-0650-6 PMC2841758 20179896

[ar70118-bib-0031] Curry Rogers, K. (2005). Titanosauria: A phylogenetic overview. In K. Curry Rogers & J. A. Wilson (Eds.), The sauropods: Evolution and Paleobiology (pp. 50–103). University of California Press. 10.1525/9780520932333-005

[ar70118-bib-0032] Curry Rogers, K. (2009). The postcranial osteology of *Rapetosaurus krausei* (Sauropoda: Titanosauria) from the Late Cretaceous of Madagascar. Journal of Vertebrate Paleontology, 29(4), 1046–1086. 10.1671/039.029.0432

[ar70118-bib-0033] de Paula e Silva, F. , Chang, H. K. , & Caetano‐Chang, M. R. (2005). Estratigrafia de subsuperfície do Grupo Bauru (K) no Estado de São Paulo. Revista Brasileira de Geociencias, 35, 77–88. 10.25249/0375-7536.20053517788

[ar70118-bib-0034] de Paula e Silva, F. , Chang, H. K. , & Caetano‐Chang, M. R. (2009). Sedimentation of the Cretaceous Bauru Group in São Paulo, Paraná Basin, Brazil. Journal of South American Earth Sciences, 28(1), 25–39. 10.1016/j.jsames.2009.02.008

[ar70118-bib-0035] de Souza, G. A. , Soares, M. B. , Weinschütz, L. C. , Wilner, E. , Lopes, R. T. , de Araújo, O. M. O. , & Kellner, A. W. A. (2021). The first edentulous ceratosaur from South America. Scientific Reports, 11(1), 1. 10.1038/s41598-021-01312-4 PMC8602317 34795306

[ar70118-bib-0036] D'Emic, M. D. (2012). The early evolution of titanosauriform sauropod dinosaurs. Zoological Journal of the Linnean Society, 166(3), 624–671. 10.1111/j.1096-3642.2012.00853.x

[ar70118-bib-0037] D'Emic, M. D. , Foreman, B. , & Jud, N. (2016). Anatomy, systematics, paleoenvironment, growth, and age of the sauropod dinosaur *Sonorasaurus thompsoni* from the Cretaceous of Arizona, USA. Journal of Paleontology, 90(1), 102–132. 10.1017/jpa.2015.67

[ar70118-bib-0038] Dias, A. N. C. , Chemale, F. , Candeiro, C. R. A. , Lana, C. C. , Guadagnin, F. , & Sales, A. S. W. (2021). Unraveling multiple tectonic events and source areas in the intracratonic Bauru Basin through combined zircon geo and thermochronological studies. Journal of South American Earth Sciences, 106, 103061. 10.1016/j.jsames.2020.103061

[ar70118-bib-0039] Dias‐Brito, D. , Musacchio, E. A. , de Castro, J. C. , Maranhão, M. S. A. S. , Suárez, J. M. , & Rodrigues, R. (2001). The Bauru group: A continental Cretaceous unit in Brazil—Concepts based on micropaleontological, oxygen isotope and stratigraphical data. Revue de Paléobiologie, 20(1), 245–304.

[ar70118-bib-0040] Fernandes, L. A. , & Basilici, G. (2009). Transition of ephemeral palustrine to aeolian deposits in a continental arid – Semi‐arid environment (Upper Cretaceous Bauru Basin, Brazil). Cretaceous Research, 30(3), 605–614. 10.1016/j.cretres.2008.12.002

[ar70118-bib-0041] Fernandes, L. A. , & Coimbra, A. M. (2000). Revisão estratigráfica da parte oriental da Bacia Bauru (Neocretáceo). Revista Brasileira de Geociencias, 30(4), 717–728.

[ar70118-bib-0042] Fernandes, L. A. , & Magalhães Ribeiro, C. M. (2015). Evolution and palaeoenvironment of the Bauru Basin (Upper Cretaceous, Brazil). Journal of South American Earth Sciences, 61, 71–90. 10.1016/j.jsames.2014.11.007

[ar70118-bib-0043] Filippi, L. S. , Juárez Valieri, R. D. , Gallina, P. A. , Méndez, A. H. , Gianechini, F. A. , & Garrido, A. C. (2024). A rebbachisaurid‐mimicking titanosaur and evidence of a Late Cretaceous faunal disturbance event in South‐West Gondwana. Cretaceous Research, 154, 105754. 10.1016/j.cretres.2023.105754

[ar70118-bib-0044] Fronimos, J. A. , & Wilson, J. A. (2017). Concavo‐convex intercentral joints stabilize the vertebral column in sauropod dinosaurs and crocodylians. Ameghiniana, 54(2), 151–176. 10.5710/AMGH.12.09.2016.3007

[ar70118-bib-0045] Fronimos, J. A. , Wilson, J. A. , & Baumiller, T. K. (2016). Polarity of concavo‐convex intervertebral joints in the necks and tails of sauropod dinosaurs. Paleobiology, 42(4), 624–642. 10.1017/pab.2016.16

[ar70118-bib-0046] Gallina, P. A. (2011). Notes on the axial skeleton of the titanosaur *Bonitasaura salgadoi* (Dinosauria‐Sauropoda). Anais da Academia Brasileira de Ciências, 83(1), 235–246. 10.1590/S0001-37652011005000001 21308348

[ar70118-bib-0047] Gallina, P. A. (2012). Histología ósea del titanosaurio *Bonitasaura salgadoi* (Dinosauria: Sauropoda) del Cretácico Superior de Patagonia. Ameghiniana, 49(3), 289–302. 10.5710/AMGH.v49i3(519)

[ar70118-bib-0048] Gallina, P. A. , & Apesteguía, S. (2015). Postcranial anatomy of *Bonitasaura salgadoi* (Sauropoda, Titanosauria) from the Late Cretaceous of Patagonia. Journal of Vertebrate Paleontology, 35(3), e924957. 10.1080/02724634.2014.924957

[ar70118-bib-0049] Gallina, P. A. , Canale, J. , & Carballido, J. L. (2021). The earliest known titanosaur sauropod dinosaur. Ameghiniana, 58, 35–51. 10.5710/AMGH.20.08.2020.3376

[ar70118-bib-0050] Ghilardi, A. M. , Aureliano, T. , Duque, R. R. C. , Fernandes, M. A. , Barreto, A. M. F. , & Chinsamy, A. (2016). A new titanosaur from the Lower Cretaceous of Brazil. Cretaceous Research, 67, 16–24. 10.1016/j.cretres.2016.07.001

[ar70118-bib-0051] Gil, L. M. , Bandeira, K. L. N. , Brusatte, S. L. , Pereira, P. V. L. G. C. , Kuhn, C. E. S. , de Souza, A. B. , Hirooka, S. S. , & Candeiro, C. R. d. A. (2020). New records of Titanosauria (Dinosauria: Sauropoda) from the Upper Cretaceous of Mid‐Western Brazil (Mato Grosso). Journal of South American Earth Sciences, 101, 102596. 10.1016/j.jsames.2020.102596

[ar70118-bib-0052] Goldberg, K. , & Garcia, A. J. V. (2000). Palaeobiogeography of the Bauru Group, a dinosaur‐bearing Cretaceous unit, northeastern Parana Basin, Brazil. Cretaceous Research, 21(2), 241–254. 10.1006/cres.2000.0207

[ar70118-bib-0053] Goloboff, P. A. (2014). Extended implied weighting. Cladistics, 30(3), 260–272. 10.1111/cla.12047 34788977

[ar70118-bib-0054] Goloboff, P. A. , & Catalano, S. A. (2016). TNT version 1.5, including a full implementation of phylogenetic morphometrics. Cladistics, 32(3), 221–238. 10.1111/cla.12160 34727670

[ar70118-bib-0055] Goloboff, P. A. , Torres, A. , & Arias, J. S. (2018). Weighted parsimony outperforms other methods of phylogenetic inference under models appropriate for morphology. Cladistics, 34(4), 407–437. 10.1111/cla.12205 34649370

[ar70118-bib-0056] Gomez, K. L. , Jose, C. , & Pol, D. (2021). The axial skeleton of *Bagualia alba* (Dinosauria: Eusauropoda) from the Early Jurassic of Patagonia. Palaeontologia Electronica, 24, 3.37A. 10.26879/1176

[ar70118-bib-0057] González Riga, B. J. (2010). Paleobiology of South American titanosaurs. In J. O. Calvo , J. Porfiri , B. J. González Riga , & D. Dos Santos (Eds.), Paleontología y dinosaurios desde America Latina (pp. 125–141). EDIUNC, Universidad Nacional de Cuyo.

[ar70118-bib-0058] González Riga, B. J. , Casal, G. A. , Fiorillo, A. R. , & Ortiz David, L. D. (2022). Taphonomy: Overview and new perspectives related to the paleobiology of giants. In A. Otero , J. L. Carballido , & D. Pol (Eds.), South American Sauropodomorph dinosaurs: Record, diversity and evolution (pp. 541–582). Springer International Publishing. 10.1007/978-3-030-95959-3_15

[ar70118-bib-0059] González Riga, B. J. , & Ortiz David, L. (2014). A new titanosaur (Dinosauria, Sauropoda) from the Upper Cretaceous (Cerro Lisandro Formation) of Mendoza Province, Argentina. Ameghiniana, 51(1), 3–25.

[ar70118-bib-0060] Griffin, C. T. , Stocker, M. R. , Colleary, C. , Stefanic, C. M. , Lessner, E. J. , Riegler, M. , Formoso, K. , Koeller, K. , & Nesbitt, S. J. (2021). Assessing ontogenetic maturity in extinct saurian reptiles. Biological Reviews, 96(2), 470–525. 10.1111/brv.12666 33289322

[ar70118-bib-0061] Ikejiri, T. , Tidwell, V. , & Trexler, D. (2005). New adult specimens of *Camarasaurus lentus* highlight ontogenetic variation within the species. In V. Tidwell & K. Carpenter (Eds.), Thunder lizards: The sauropodomorph dinosaurs (pp. 154–179). Indiana University Press.

[ar70118-bib-0062] Iori, F. V. , Marinho, T. S. , da Silva Junior, J. C. G. , & Paschoa, L. S. (2017). A paleofauna da Formação São José do Rio Preto (Bacia Bauru, Cretáceo Superior). Boletim de resumos, 144.

[ar70118-bib-0063] Irmis, R. B. (2007). Axial skeleton ontogeny in the Parasuchia (Archosauria: Pseudosuchia) and its implications for ontogenetic determination in archosaurs. Journal of Vertebrate Paleontology, 27, 350–361. 10.1671/0272-4634(2007)27[350:ASOITP]2.0.CO;2

[ar70118-bib-0064] Janensch, W. (1950). Die wirbelsaule von *Brachiosaurus brancai* . Palaeontographica, 3(7), 27–93.

[ar70118-bib-0065] Kellner, A. W. A. , Campos, D. d. A. , de Azevedo, S. A. K. , Trotta, M. N. F. , Henriques, D. D. , Craik, M. , & Silva, H. d. P. (2006). On a new titanosaur sauropod from the Bauru Group, Late Cretaceous of Brazil. Boletim Do Museu Nacional, Nova Série (Geologia), 74, 1–31.

[ar70118-bib-0066] Kellner, A. W. A. , & de Azevedo, S. A. K. (1999). A new sauropod dinosaur (Titanosauria) from the Late Cretaceous of Brazil. National Science Museum Monographs, 15(111), e142.

[ar70118-bib-0067] Kellner, A. W. A. , Weinschutz, L. , Holgado, B. , Bantim, R. , & Sayão, J. (2019). A new toothless pterosaur (Pterodactyloidea) from southern Brazil with insights into the paleoecology of a Cretaceous desert. Anais da Academia Brasileira de Ciências, 91(2), e20190768. 10.1590/0001-3765201920190768 31432888

[ar70118-bib-0068] Klein, N. , & Sander, P. M. (2008). Ontogenetic stages in the bone histology of sauropod dinosaurs. Paleobiology, 34, 247–263. 10.1666/0094-8373(2008)034[0247:OSITLB]2.0.CO;2

[ar70118-bib-0069] Ksepka, D. T. , & Norell, M. A. (2006). *Erketu ellisoni*, a long‐necked sauropod from Bor Guvé (Dornogov Aimag, Mongolia). American Museum Novitates, 2006, 1–16. 10.1206/0003-0082(2006)3508[1:EEALSF]2.0.CO;2

[ar70118-bib-0070] Langer, M. C. , Delcourt, R. , Montefeltro, F. C. , Da Silva Júnior, J. C. G. , Soler, M. , Ferreira, G. S. , Ruiz, J. V. , Barcelos, L. A. , Onary, S. , Marsola, J. C. d. A. , Castro, M. C. , Cidade, G. M. , & Batezelli, A. (2022). The Bauru Basin in São Paulo and its tetrapods. Derbyana, 43, e776. 10.14295/derb.v43.776

[ar70118-bib-0071] Langer, M. C. , Martins, N. , Manzig, P. , Ferreira, G. , Marsola, J. C. d. A. , Fortes, E. , Lima, R. , Sant'ana, L. , Vidal, L. , Lorençato, R. , & Ezcurra, M. (2019). A new desert‐dwelling dinosaur (Theropoda, Noasaurinae) from the Cretaceous of south Brazil. Scientific Reports, 9, 9379. 10.1038/s41598-019-45306-9 PMC6594977 31243312

[ar70118-bib-0072] Lindoso, R. M. , Marinho, T. S. , Santucci, R. M. , Medeiros, M. A. A. , & Carvalho, I. S. (2013). A titanosaur (Dinosauria: Sauropoda) osteoderm from the Alcântara Formation (Cenomanian), São Luís Basin, northeastern Brazil. Cretaceous Research, 45, 43–48. 10.1016/j.cretres.2013.07.005

[ar70118-bib-0073] Machado, E. B. , Avilla, L. S. , Nava, W. R. , Campos, D. A. , & Kellner, A. W. A. (2013). A new titanosaur sauropod from the Late Cretaceous of Brazil. Zootaxa, 3701(3), 301–321. 10.11646/zootaxa.3701.3.1 26191585

[ar70118-bib-0074] Madsen, J. H., Jr. , McIntosh, J. S. , & Berman, D. S. (1995). Skull and atlas‐axis complex of the Upper Jurassic sauropod *Camarasaurus* cope (Reptilia: Saurischia). Bulletin of Carnegie Museum of Natural History, 31, 1–115.

[ar70118-bib-0075] Mannion, P. D. (2011). A reassessment of *Mongolosaurus haplodon* Gilmore, 1933, a titanosaurian sauropod dinosaur from the Early Cretaceous of Inner Mongolia, People's Republic of China. Journal of Systematic Palaeontology, 9(3), 355–378. 10.1080/14772019.2010.527379

[ar70118-bib-0076] Mannion, P. D. , & Upchurch, P. (2010). Completeness metrics and the quality of the sauropodomorph fossil record through geological and historical time. Paleobiology, 36(2), 283–302. 10.2307/40792289

[ar70118-bib-0077] Mannion, P. D. , Upchurch, P. , Barnes, R. , & Mateus, O. (2013). Osteology of the Late Jurassic Portuguese sauropod dinosaur *Lusotitan atalaiensis* (Macronaria) and the evolutionary history of basal titanosauriforms. Zoological Journal of the Linnean Society, 168(1), 98–206. 10.1111/zoj.12029

[ar70118-bib-0078] Mannion, P. D. , Upchurch, P. , Carrano, M. , & Barrett, P. (2011). Testing the effect of the rock record on diversity: A multidisciplinary approach to elucidating the generic richness of sauropodomorph dinosaurs through time. Biological Reviews of the Cambridge Philosophical Society, 86, 81–157. 10.1111/j.1469-185X.2010.00139.x 20412186

[ar70118-bib-0079] Mannion, P. D. , Upchurch, P. , Schwarz, D. , & Wings, O. (2019). Taxonomic affinities of the putative titanosaurs from the Late Jurassic Tendaguru Formation of Tanzania: Phylogenetic and biogeographic implications for eusauropod dinosaur evolution. Zoological Journal of the Linnean Society, 185(3), 784–909. 10.1093/zoolinnean/zly068

[ar70118-bib-0080] Manzig, P. C. , Kellner, A. W. A. , Weinschütz, L. C. , Fragoso, C. E. , Vega, C. S. , Guimarães, G. B. , Godoy, L. C. , Liccardo, A. , Ricetti, J. H. Z. , & de Moura, C. C. (2014). Discovery of a rare pterosaur bone bed in a Cretaceous desert with insights on ontogeny and behavior of flying reptiles. PLoS One, 9(8), e100005. 10.1371/journal.pone.0100005 PMC4131874 25118592

[ar70118-bib-0081] Marinho, T. S. , & Iori, F. V. (2011). A large titanosaur (Dinosauria, Sauropoda) osteoderm with possible bite marks from Ibirá, São Paulo state, Brazil. In I. S. Carvalho , N. K. Srivastava , O. Strochschoen, Jr. , & C. C. Lana (Eds.), Paleontologia: Cenários de Vida (Vol. 4, 1st ed., pp. 339–349). Interciência.

[ar70118-bib-0082] Marsh, O. C. (1878). Principal characters of American Jurassic dinosaurs. American Journal of Science, s3‐16(95), 411–416. 10.2475/ajs.s3-16.95.411

[ar70118-bib-0083] Martinelli, A. G. , Marinho, T. S. , Filippi, L. S. , Ribeiro, L. C. B. , Ferraz, M. L. F. , Cavellani, C. L. , & Teixeira, V. P. A. (2015). Cranial bones and atlas of titanosaurs (Dinosauria, Sauropoda) from Late Cretaceous (Bauru Group) of Uberaba, Minas Gerais state, Brazil. Journal of South American Earth Sciences, 61, 164–170. 10.1016/j.jsames.2015.02.009

[ar70118-bib-0084] Martinelli, A. G. , & Teixeira, V. P. A. (2015). The Late Cretaceous vertebrate record from the Bauru Group in the Triângulo Mineiro, southeastern Brazil. Boletín Geológico y Minero, 126(1), 129–158.

[ar70118-bib-0085] Medeiros, M. A. , Lindoso, R. M. , Mendes, I. D. , & Carvalho, I. S. (2014). The Cretaceous (Cenomanian) continental record of the Laje do Coringa flagstone (Alcântara Formation), northeastern South America. Journal of South American Earth Sciences, 53, 50–58. 10.1016/j.jsames.2014.04.002

[ar70118-bib-0086] Menegazzo, M. C. , Catuneanu, O. , & Chang, H. K. (2016). The South American retroarc foreland system: The development of the Bauru Basin in the back‐bulge province. Marine and Petroleum Geology, 73, 131–156. 10.1016/j.marpetgeo.2016.02.027

[ar70118-bib-0087] Militello, M. , Otero, A. , & Carballido, J. L. (2026). The occiput of *Amargasaurus* (Sauropoda, Dicraeosauridae): Reconstruction of the craniocervical muscular insertions with comments on feeding strategy. Journal of Anatomy, 1, 1–23. 10.1111/joa.70071 PMC12779418 41284356

[ar70118-bib-0088] Navarro, B. A. , Ghilardi, A. M. , Aureliano, T. , Díaz, V. , Bandeira, K. L. N. , Cattaruzzi, A. G. S. , Iori, F. V. , Martine, A. M. , Carvalho, A. B. , Anelli, L. E. , Fernandes, M. A. , & Zaher, H. (2022). A new nanoid titanosaur (Dinosauria: Sauropoda) from the Upper Cretaceous of Brazil. Ameghiniana, 59(5), 317–354. 10.5710/AMGH.25.08.2022.3477

[ar70118-bib-0089] Navarro, B. A. , Giaretta, A. A. , Fernandes, M. A. , Carvalho, A. B. , & Zaher, H. (2025). First dinosaur ichnofauna from the Bauru Group indicates Cenomanian–Turonian events led to an ‘ornithischian hiatus’ in the Upper Cretaceous of Southeast Brazil. Cretaceous Research, 168, 106075. 10.1016/j.cretres.2024.106075

[ar70118-bib-0090] Otero, A. , Carballido, J. L. , Salgado, L. , Canudo, J. I. , & Garrido, A. C. (2021). Report of a giant titanosaur sauropod from the Upper Cretaceous of Neuquén Province, Argentina. Cretaceous Research, 122, 104754. 10.1016/j.cretres.2021.104754

[ar70118-bib-0091] Owen, R. (1842). Report on British fossil reptiles. Part II Report of the Eleventh Meeting of the British Association for the Advancement of Science 11 60 204.

[ar70118-bib-0092] Pêgas, R. V. (2024). A taxonomic note on the tapejarid pterosaurs from the pterosaur graveyard site (Caiuá Group, ?Early Cretaceous of southern Brazil): Evidence for the presence of two species. Historical Biology, 37(5), 1277–1298. 10.1080/08912963.2024.2355664

[ar70118-bib-0093] Pereira, P. V. L. G. C. , Bandeira, K. L. N. , Vidal, L. S. , Ribeiro, T. B. , Candeiro, C. R. A. , & Bergqvist, L. P. (2024). A new sauropod species from north‐western Brazil: Biomechanics and the radiation of Titanosauria (Sauropoda: Somphospondyli). Zoological Journal of the Linnean Society, 202(4), zlae054. 10.1093/zoolinnean/zlae054

[ar70118-bib-0094] Pereira, P. V. L. G. C. , Marinho, T. S. , Candeiro, C. R. A. , & Bergqvist, L. P. (2018). A new titanosaurian (Sauropoda, Dinosauria) osteoderm from the Cretaceous of Brazil and its significance. Ameghiniana, 55(6), 644–650. 10.5710/AMGH.26.08.2018.3168

[ar70118-bib-0095] Pérez Moreno, A. , Jose, C. , Otero, A. , Salgado, L. , & Calvo, J. (2021). The axial skeleton of *Rinconsaurus caudamirus* (Sauropoda: Titanosauria) from the Late Cretaceous of Patagonia, Argentina. Ameghiniana, 59(1), 1–47. 10.5710/AMGH.13.09.2021.3427

[ar70118-bib-0096] Pérez Moreno, A. , Otero, A. , Carballido, J. L. , Salgado, L. , & Calvo, J. O. (2022). The appendicular skeleton of *Rinconsaurus caudamirus* (Sauropoda: Titanosauria) from the Upper Cretaceous of Patagonia, Argentina. Cretaceous Research, 142, 105389. 10.1016/j.cretres.2022.105389

[ar70118-bib-0097] Poropat, S. F. , Kundrát, M. , Mannion, P. D. , Upchurch, P. , Tischler, T. R. , & Elliott, D. A. (2021). Second specimen of the Late Cretaceous Australian sauropod dinosaur *Diamantinasaurus matildae* provides new anatomical information on the skull and neck of early titanosaurs. Zoological Journal of the Linnean Society, 192(2), 610–674. 10.1093/zoolinnean/zlaa173

[ar70118-bib-0098] Poropat, S. F. , Mannion, P. D. , Rigby, S. , Duncan, R. , Pentland, A. , Bevitt, J. , Sloan, T. , & Elliott, D. (2023). A nearly complete skull of the sauropod dinosaur *Diamantinasaurus matildae* from the Upper Cretaceous Winton formation of Australia and implications for the early evolution of titanosaurs. Royal Society Open Science, 10, 221618. 10.1098/rsos.221618 PMC10090887 37063988

[ar70118-bib-0099] Poropat, S. F. , Tosolini, A.‐M. P. , Beeston, S. L. , Enchelmaier, M. J. , Pentland, A. H. , Mannion, P. D. , Upchurch, P. , Chin, K. , Korasidis, V. A. , Bell, P. R. , Enriquez, N. J. , Holman, A. I. , Brosnan, L. M. , Elson, A. L. , Tripp, M. , Scarlett, A. G. , Godel, B. , Madden, R. H. C. , Rickard, W. D. A. , … Grice, K. (2025). Fossilized gut contents elucidate the feeding habits of sauropod dinosaurs. Current Biology, 35(11), 2597–2613. 10.1016/j.cub.2025.04.053 40494280

[ar70118-bib-0100] Poropat, S. F. , Upchurch, P. , Mannion, P. D. , Hocknull, S. A. , Kear, B. P. , Sloan, T. , Sinapius, G. H. K. , & Elliott, D. A. (2015). Revision of the sauropod dinosaur *Diamantinasaurus matildae* Hocknull et al. 2009 from the mid‐Cretaceous of Australia: Implications for Gondwanan titanosauriform dispersal. Gondwana Research, 27(3), 995–1033. 10.1016/j.gr.2014.03.014

[ar70118-bib-0101] Powell, J. E. (1987). Morfología del esqueleto axial de los dinosaurios titanosauridos (Saurischia, Sauropoda) del Estado de Minas Gerais, Brasil. Anais, 1, 155–171.

[ar70118-bib-0102] Powell, J. E. (1992). Osteología de *Saltasaurus loricatus* (Sauropoda‐Titanosauridae) del Cretácico Superior argentino. Actas, 2, 165–230.

[ar70118-bib-0103] Powell, J. E. (2003). Revision of South American titanosaurids dinosaurs: Palaeobiological, palaeobiogeographical and phylogenetic aspects. Records of the Queen Victoria Museum, 111(1), 173.

[ar70118-bib-0104] Reis, L. F. D. S. , Ghilardi, A. M. , & Fernandes, M. (2022). Bite traces in a sauropod rib from the Upper Cretaceous São José do Rio Preto Formation (Bauru Basin), Brazil. Historical Biology, 35(8), 1329–1340. 10.1080/08912963.2022.2090248

[ar70118-bib-0105] Salgado, L. , & Carvalho, I. S. (2008). *Uberabatitan ribeiroi*, a new titanosaur from the Marília Formation (Bauru Group,Upper Cretaceous), Minas Gerais, Brazil. Palaeontology, 51(4), 881–901. 10.1111/j.1475-4983.2008.00781.x

[ar70118-bib-0106] Salgado, L. , Coria, R. A. , & Calvo, J. O. (1997). Evolution of titanosaurid sauropods: Phylogenetic analysis based on the postcranial evidence. Ameghiniana, 34(1), 3–32.

[ar70118-bib-0107] Santucci, R. M. , & Arruda‐Campos, A. C. D. (2011). A new sauropod (Macronaria, Titanosauria) from the Adamantina Formation, Bauru Group, Upper Cretaceous of Brazil and the phylogenetic relationships of Aeolosaurini. Zootaxa, 3085(1), 1–33. 10.11646/zootaxa.3085.1.1

[ar70118-bib-0108] Santucci, R. M. , & Filippi, L. S. (2022). Last titans: Titanosaurs from the Campanian–Maastrichtian age. In A. Otero , J. L. Carballido , & D. Pol (Eds.), South American Sauropodomorph dinosaurs: Record, diversity and evolution (pp. 341–391). Springer International Publishing. 10.1007/978-3-030-95959-3_10

[ar70118-bib-0109] Silva Junior, J. C. G. , Marinho, T. S. , Martinelli, A. G. , & Langer, M. C. (2019). Osteology and systematics of *Uberabatitan ribeiroi* (Dinosauria; Sauropoda): A Late Cretaceous titanosaur from Minas Gerais, Brazil. Zootaxa, 4577(3), 401–438. 10.11646/zootaxa.4577.3.1 31715707

[ar70118-bib-0110] Simões, T. R. , Wilner, E. , Caldwell, M. W. , Weinschütz, L. C. , & Kellner, A. W. A. (2015). A stem acrodontan lizard in the Cretaceous of Brazil revises early lizard evolution in Gondwana. Nature Communications, 6(1), 8149. 10.1038/ncomms9149 PMC4560825 26306778

[ar70118-bib-0111] Stevens, K. A. (2013). The articulation of sauropod necks: Methodology and mythology. PLoS One, 8(10), e78572. 10.1371/journal.pone.0078572 PMC3812995 24205266

[ar70118-bib-0112] Stevens, K. A. , & Parrish, J. M. (2005). Neck posture, dentition, and feeding strategies in Jurassic sauropod dinosaurs. In V. Tidwell & K. Carpenter (Eds.), Thunder lizards: The sauropodomorph dinosaurs (pp. 212–232). Indianan University Press.

[ar70118-bib-0113] Taylor, M. P. (2022). Almost all known sauropod necks are incomplete and distorted. PeerJ, 10, e12810. 10.7717/peerj.12810 PMC8793732 35127288

[ar70118-bib-0114] Taylor, M. P. , & Wedel, M. J. (2013). Why sauropods had long necks; and why giraffes have short necks. PeerJ, 1, e36. 10.7717/peerj.36 PMC3628838 23638372

[ar70118-bib-0115] Tschopp, E. , & Mateus, O. (2012). The skull and neck of a new flagellicaudatan sauropod from the Morrison Formation and its implication for the evolution and ontogeny of diplodocid dinosaurs. Journal of Systematic Palaeontology, 11(7), 853–888. 10.1080/14772019.2012.746589

[ar70118-bib-0116] Tschopp, E. , Mateus, O. , & Benson, R. (2015). A specimen‐level phylogenetic analysis and taxonomic revision of Diplodocidae (Dinosauria, Sauropoda). PeerJ, 3, e857. 10.7717/peerj.857 PMC4393826 25870766

[ar70118-bib-0117] Tykoski, R. S. , & Fiorillo, A. R. (2017). An articulated cervical series of *Alamosaurus sanjuanensis* Gilmore, 1922 (Dinosauria, Sauropoda) from Texas: New perspective on the relationships of North America's last giant sauropod. Journal of Systematic Palaeontology, 15(5), 339–364. 10.1080/14772019.2016.1183150

[ar70118-bib-0141] Upchurch, P. (1998). The phylogenetic relationships of sauropod dinosaurs. Zoological Journal of the Linnean Society, 124(1), 43–103. 10.1111/j.1096-3642.1998.tb00569.x

[ar70118-bib-0118] Upchurch, P. , Barrett, P. M. , & Dodson, P. (2004). Sauropoda. In D. Weishampel , P. Dodson , & H. Osmólska (Eds.), The Dinosauria (2nd ed., pp. 259–322). University of California Press.

[ar70118-bib-0119] Vidal, D. , Mocho, P. , Aberasturi, A. , Sanz, J. L. , & Ortega, F. (2020). High browsing skeletal adaptations in *Spinophorosaurus* reveal an evolutionary innovation in sauropod dinosaurs. Scientific Reports, 10, 6638. 10.1038/s41598-020-63439-0 PMC7171156 32313018

[ar70118-bib-0120] Vila, B. , Sellés, A. , Moreno‐Azanza, M. , Razzolini, N. L. , Gil‐Delgado, A. , Canudo, J. I. , & Galobart, À. (2022). A titanosaurian sauropod with Gondwanan affinities in the latest Cretaceous of Europe. Nature Ecology & Evolution, 6(3), 288–296. 10.1038/s41559-021-01651-5 35132183

[ar70118-bib-0121] Wedel, M. J. (2003). The evolution of vertebral pneumaticity in sauropod dinosaurs. Journal of Vertebrate Paleontology, 23(2), 344–357.

[ar70118-bib-0122] Whitlock, J. A. (2011). A phylogenetic analysis of Diplodocoidea (Saurischia: Sauropoda). Zoological Journal of the Linnean Society, 161(4), 872–915. 10.1111/j.1096-3642.2010.00665.x

[ar70118-bib-0123] Whitlock, J. A. , & Wilson Mantilla, J. A. (2021). The Late Jurassic sauropod dinosaur *‘Morosaurus’ agilis* Marsh, 1889 reexamined and reinterpreted as a dicraeosaurid. Journal of Vertebrate Paleontology, 40(6), e1780600. 10.1080/02724634.2020.1780600

[ar70118-bib-0124] Wilson, J. A. (1999). A nomenclature for vertebral laminae in sauropods and other saurischian dinosaurs. Journal of Vertebrate Paleontology, 19(4), 639–653.

[ar70118-bib-0125] Wilson, J. A. (2002). Sauropod dinosaur phylogeny: Critique and cladistic analysis. Zoological Journal of the Linnean Society, 136(2), 215–275. 10.1046/j.1096-3642.2002.00029.x

[ar70118-bib-0126] Wilson, J. A. (2006a). An overview of titanosaur evolution and phylogeny. Actas, 3, 169–190.

[ar70118-bib-0127] Wilson, J. A. (2006b). Anatomical nomenclature of fossil vertebrates: Standardized terms or ‘lingua franca'? Journal of Vertebrate Paleontology, 26(3), 511–518. 10.1671/0272-4634(2006)26[511:ANOFVS]2.0.CO;2

[ar70118-bib-0128] Wilson, J. A. (2012). New vertebral laminae and patterns of serial variation in vertebral laminae of sauropod dinosaurs. Contributions from the Museum of Paleontology, University of Michigan, 32(7), 91–110.

[ar70118-bib-0129] Wilson, J. A. , D'Emic, M. D. , Ikejiri, T. , Moacdieh, E. M. , & Whitlock, J. A. (2011). A nomenclature for vertebral fossae in sauropods and other saurischian dinosaurs. PLoS One, 6(2), e17114. 10.1371/journal.pone.0017114 PMC3046170 21386963

[ar70118-bib-0130] Wilson, J. A. , & Mohabey, D. M. (2006). A titanosauriform (Dinosauria: Sauropoda) axis from the Lameta Formation (Upper Cretaceous: Maastrichtian) of Nand, central India. Journal of Vertebrate Paleontology, 26(2), 471–479. 10.1671/0272-4634(2006)26[471:ATDSAF]2.0.CO;2

[ar70118-bib-0131] Wilson, J. A. , Pol, D. , Carvalho, A. B. , & Zaher, H. (2016). The skull of the titanosaur *Tapuiasaurus macedoi* (Dinosauria: Sauropoda), a basal titanosaur from the Lower Cretaceous of Brazil. Zoological Journal of the Linnean Society, 178(3), 611–662. 10.1111/zoj.12420

[ar70118-bib-0132] Wilson, J. A. , & Sereno, P. C. (1998). Early evolution and higher‐level phylogeny of sauropod dinosaurs. Journal of Vertebrate Paleontology, 18(s2), 1–79. 10.1080/02724634.1998.10011115

[ar70118-bib-0133] Wilson, J. A. , & Upchurch, P. (2009). Redescription and reassessment of the phylogenetic affinities of *Euhelopus zdanskyi* (Dinosauria: Sauropoda) from the Early Cretaceous of China. Journal of Systematic Palaeontology, 7, 199–239. 10.1017/S1477201908002691

[ar70118-bib-0134] Woodward, H. N. , & Lehman, T. M. (2009). Bone histology and microanatomy of *Alamosaurus sanjuanensis* (Sauropoda: Titanosauria) from the Maastrichtian of Big Bend National Park, Texas. Journal of Vertebrate Paleontology, 29(3), 807–821. 10.1671/039.029.0310

[ar70118-bib-0135] Yang, Z. J. , & Zhao, X.‐J. (1972). *Mamenchisaurus hochuanensis* sp. nov. In Monograph (ser. A), (Vol. 8, pp. 1–30). Institute of Vertebrate Paleontology and Paleoanthropology.

[ar70118-bib-0136] Zaher, H. , Pol, D. , Carvalho, A. B. , Nascimento, P. M. , Riccomini, C. , Larson, P. , Juarez‐Valieri, R. , Pires‐Domingues, R. , Da Silva, N. J. , & Campos, D. d. A. (2011). A complete skull of an Early Cretaceous sauropod and the evolution of advanced titanosaurians. PLoS One, 6(2), e16663. 10.1371/journal.pone.0016663 PMC3034730 21326881

[ar70118-bib-0137] Zhang, X.‐Q. , Li, D.‐Q. , Xie, Y. , & You, H.‐L. (2020). Redescription of the cervical vertebrae of the mamenchisaurid sauropod *Xinjiangtitan shanshanesis* Wu et al. 2013. Historical Biology, 32(6), 803–822. 10.1080/08912963.2018.1539970

[ar70118-bib-0138] Zurriaguz, V. L. , & Cerda, I. A. (2017). Caudal pneumaticity in derived titanosaurs (Dinosauria: Sauropoda). Cretaceous Research, 73, 14–24. 10.1016/j.cretres.2017.01.001

[ar70118-bib-0139] Zurriaguz, V. L. , Martinelli, A. G. , Citton, P. , Kaluza, J. , & Cerda, I. (2025). The atlas‐axis complex in the titanosaur *Neuquensaurus australis* (Dinosauria: Sauropoda). Historical Biology, 38(1), 1–10. 10.1080/08912963.2025.2486387

[ar70118-bib-0140] Zurriaguz, V. L. , & Powell, J. E. (2015). New contributions to the presacral osteology of *Saltasaurus loricatus* (Sauropoda, Titanosauria) from the upper cretaceous of northern Argentina. Cretaceous Research, 54, 283–300. 10.1016/j.cretres.2014.12.012

