# Supplementary material for: An unusual titanosaur axis from the Upper Cretaceous of Brazil and its significance for sauropod anatomy and systematics
Source: Anat Rec (Hoboken). 2026 Jan 6;309(11):3137–68. doi: 10.1002/ar.70118 (PMC13644894; doi:10.1002/ar.70118)
Supplement: Supplementary file 1 [file AR-309-3137-s002.pdf]

SUPPORTING INFORMATION

for

# An unusual titanosaur axis from the Upper Cretaceous of Brazil and its significance for sauropod anatomy and systematics

Bruno A. Navarro, Jeffrey A. Wilson Mantilla, Jhonata H. A. Martins, Fabiano V. Iori, Leonardo S. Paschoa, Alberto B. Carvalho, Joseli M. Piranha, and Hussam Zaher

**This file contains:**

**1. Equal weighting analysis data** ..... 2
1.1. Strict consensus tree ..... 2
1.2. List of apomorphies ..... 3
**2. Implied weighting analysis data** ..... 18
2.1. Strict consensus tree ..... 18
2.2. List of synapomorphies ..... 19

**Other Supporting Information files include:**

1. Data matrix (.NEX)
2. Data matrix (.TNT)
3. Equal weights analysis output (.OUT)
4. Equal weights analysis MPTs (.TRE)
5. Equal weights analysis strict consensus tree (.TRE)
6. Implied weights analysis output (.OUT)
7. Implied weights analysis MPTs (.TRE)
8. Implied weights analysis strict consensus tree (.TRE)

# 1. Equal weighting analysis data

## 1.1. Strict consensus tree

Strict consensus of 261 trees (0 taxa excluded)

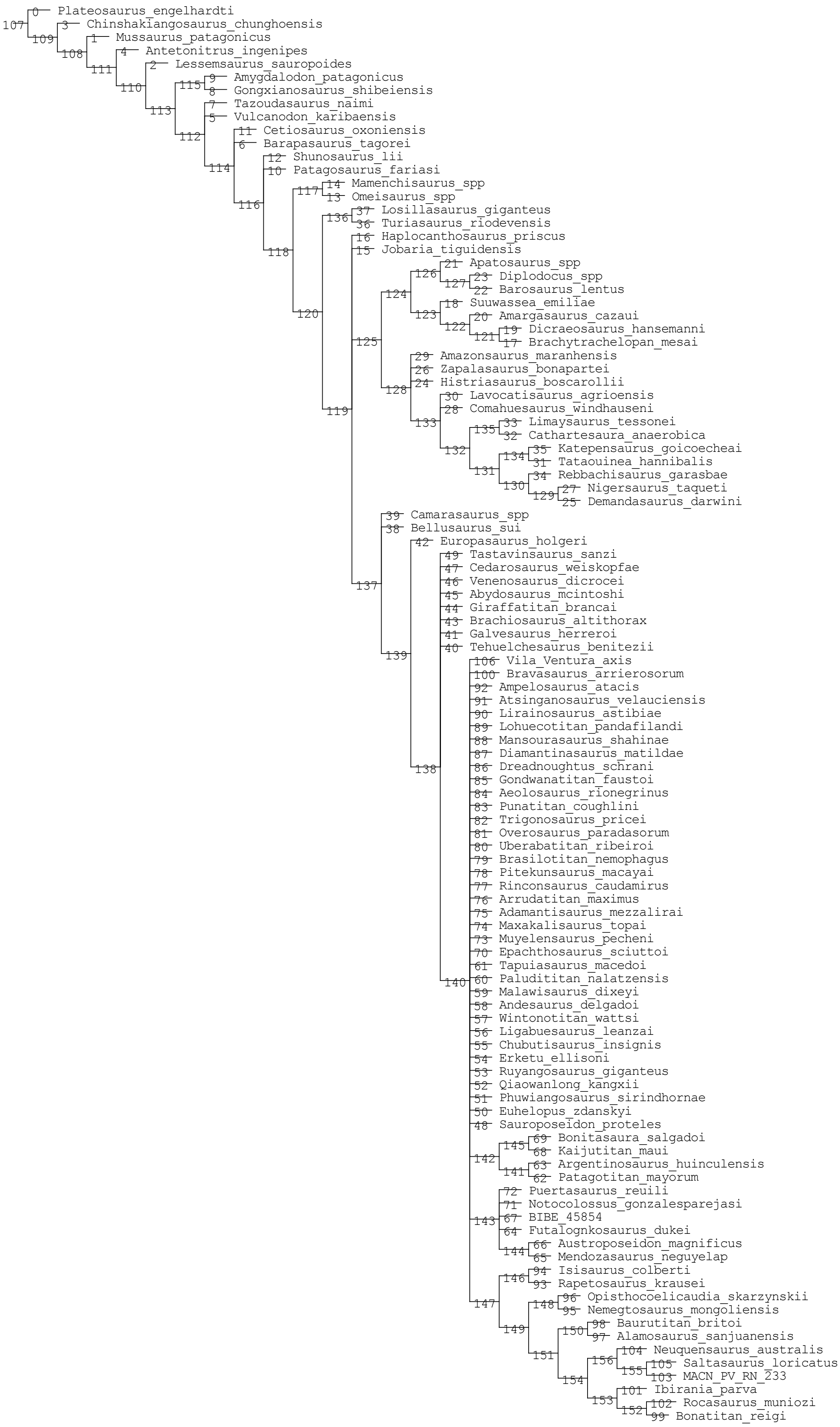

## 1.2. List of apomorphies

*Plateosaurus engelhardti*
No autapomorphies

*Mussaurus patagonicus*
Char. 86: 0 → 1
Char. 417: 0 → 1

*Lessemsaurus sauropoides*
Char. 123: 0 → 1
Char. 390: 0 → 1

*Chinshakiangosaurus chunghoensis*
Char. 101: 0 → 1

*Antetonitrus ingenipes*
Char. 162: 0 → 1
Char. 367: 0 → 1

*Vulcanodon karibaensis*
Char. 249: 0 → 1
Char. 385: 1 → 0
Char. 395: 1 → 0

*Barapasaurus tagorei*
Char. 176: 0 → 1
Char. 178: 0 → 1
Char. 192: 0 → 1
Char. 255: 2 → 1
Char. 274: 1 → 0
Char. 275: 1 → 0
Char. 367: 0 → 1
Char. 414: 0 → 1

*Tazoudasaurus naimi*
Char. 105: 1 → 0
Char. 172: 1 → 0
Char. 180: 0 → 1
Char. 258: 1 → 2
Char. 274: 1 → 0
Char. 275: 1 → 0
Char. 305: 0 → 1
Char. 328: 0 → 2
Char. 355: 0 → 1
Char. 370: 0 → 1
Char. 425: 0 → 1

*Gongxianosaurus shibeiensis*
No autapomorphies

*Amygdalodon patagonicus*
Char. 176: 0 → 1

*Patagosaurus fariasi*
Char. 180: 0 → 1
Char. 258: 1 → 2
Char. 302: 1 → 0

*Cetiosaurus oxoniensis*
Char. 123: 0 → 1
Char. 130: 0 → 2
Char. 155: 0 → 1
Char. 176: 0 → 2
Char. 193: 0 → 1
Char. 221: 1 → 2
Char. 280: 0 → 1
Char. 341: 0 → 1
Char. 411: 0 → 1

*Shunosaurus lii*
Char. 83: 0 → 1
Char. 108: 0 → 1
Char. 113: 0 → 1
Char. 114: 01 → 2
Char. 132: 0 → 1
Char. 144: 0 → 1
Char. 155: 0 → 1
Char. 164: 0 → 1
Char. 175: 02 → 1
Char. 192: 0 → 1
Char. 209: 1 → 0
Char. 267: 0 → 1
Char. 308: 1 → 0
Char. 328: 0 → 1
Char. 356: 0 → 1
Char. 361: 0 → 1

*Omeisaurus* spp.
Char. 144: 0 → 1
Char. 192: 0 → 1
Char. 193: 0 → 1
Char. 298: 1 → 2
Char. 308: 1 → 0
Char. 345: 0 → 1
Char. 357: 1 → 0
Char. 384: 0 → 1
Char. 413: 0 → 2
Char. 415: 0 → 1
Char. 435: 1 → 0
Char. 436: 1 → 0

*Mamenchisaurus* spp.
Char. 2: 0 → 1
Char. 15: 1 → 0
Char. 60: 1 → 0
Char. 65: 1 → 0
Char. 150: 0 → 1
Char. 169: 0 → 1
Char. 194: 1 → 2
Char. 203: 0 → 1
Char. 222: 0 → 1
Char. 223: 0 → 2
Char. 229: 0 → 3
Char. 258: 1 → 2
Char. 280: 0 → 1
Char. 349: 0 → 1
Char. 361: 0 → 1
Char. 374: 0 → 1
Char. 419: 0 → 1
Char. 445: 0 → 1

*Jobaria tiguidensis*
Char. 114: 12 → 0
Char. 127: 0 → 1
Char. 128: 0 → 1
Char. 134: 1 → 0
Char. 144: 0 → 1
Char. 162: 1 → 0
Char. 174: 02 → 1
Char. 192: 0 → 1
Char. 206: 2 → 1
Char. 274: 1 → 0
Char. 275: 3 → 0
Char. 287: 1 → 0
Char. 314: 1 → 0
Char. 450: 1 → 2

*Haplocanthosaurus priscus*
Char. 128: 0 → 1
Char. 153: 2 → 0
Char. 156: 0 → 1
Char. 175: 2 → 1
Char. 179: 0 → 1
Char. 201: 1 → 0
Char. 202: 0 → 1
Char. 208: 0 → 1
Char. 254: 0 → 1
Char. 277: 01 → 2
Char. 278: 0 → 1
Char. 413: 0 → 1

*Brachytrachelopan mesai*
No autapomorphies

*Suuwassea emiliae*
Char. 1: 0 → 1
Char. 101: 1 → 2
Char. 105: 0 → 1
Char. 168: 1 → 2
Char. 229: 2 → 1
Char. 302: 1 → 2
Char. 450: 1 → 0

*Dicraeosaurus hansemanni*
Char. 144: 0 → 1

*Amargasaurus cazaui*
Char. 46: 0 → 2
Char. 76: 0 → 1
Char. 145: 0 → 1
Char. 152: 1 → 0
Char. 178: 0 → 2
Char. 298: 1 → 2
Char. 308: 1 → 0
Char. 331: 0 → 1
Char. 435: 1 → 2
Char. 439: 1 → 0

*Apatosaurus* spp.
Char. 77: 1 → 0
Char. 128: 0 → 1
Char. 283: 0 → 1
Char. 302: 1 → 2
Char. 308: 1 → 0
Char. 340: 0 → 1
Char. 354: 0 → 1
Char. 377: 0 → 1
Char. 383: 0 → 1
Char. 402: 1 → 0
Char. 417: 0 → 1
Char. 419: 0 → 1

*Barosaurus lentus*
Char. 217: 1 → 0
Char. 302: 1 → 0

*Diplodocus* spp.
Char. 156: 0 → 1
Char. 205: 0 → 1

*Histriasaurus boscarollii*
Char. 156: 0 → 1
Char. 416: 0 → 1

*Demandasaurus darwini*
Char. 101: 01 → 2
Char. 239: 0 → 1
Char. 248: 1 → 0

*Zapalasaurus bonapartei*
Char. 231: 0 → 1
Char. 235: 3 → 1
Char. 240: 0 → 1
Char. 244: 1 → 0

*Nigersaurus taqueti*
Char. 180: 0 → 1

*Comahuesaurus windhauseni*
Char. 168: 1 → 2
Char. 169: 1 → 2
Char. 196: 0 → 1
Char. 217: 1 → 0
Char. 417: 0 → 1

*Amazonsaurus maranhensis*
Char. 417: 0 → 1

*Lavocatisaurus agrioensis*
Char. 91: 1 → 0
Char. 126: 1 → 0

*Tataouinea hannibalis*
Char. 230: 0 → 1
Char. 231: 0 → 1
Char. 239: 0 → 1

*Cathartesaura anaerobica*
Char. 234: 0 → 1
Char. 254: 0 → 1

*Limaysaurus tessonei*
No autapomorphies

*Rebbachisaurus garasbae*
Char. 116: 1 → 0

*Katepensaurus goicoecheai*
No autapomorphies

*Turiasaurus riodevensis*
Char. 114: 12 → 0
Char. 122: 0 → 1
Char. 128: 0 → 1
Char. 136: 0 → 1
Char. 144: 0 → 1
Char. 150: 0 → 1
Char. 165: 1 → 0

*Losillasaurus giganteus*
Char. 178: 0 → 1
Char. 187: 0 → 1
Char. 202: 0 → 1
Char. 227: 0 → 1

*Bellusaurus sui*
Char. 43: 0 → 1
Char. 208: 0 → 1
Char. 222: 0 → 1
Char. 223: 0 → 2
Char. 229: 0 → 3
Char. 355: 0 → 1
Char. 360: 1 → 0
Char. 428: 0 → 1
Char. 439: 1 → 0

*Camarasaurus* spp.
Char. 23: 1 → 0
Char. 59: 0 → 1
Char. 136: 0 → 1

Char. 150: 0 → 1
Char. 162: 1 → 0
Char. 165: 1 → 0
Char. 194: 1 → 2
Char. 275: 3 → 1
Char. 415: 0 → 1
Char. 425: 0 → 1
Char. 429: 0 → 2
Char. 449: 0 → 1
Char. 452: 1 → 0

*Tehuelchesaurus benitezii*
Char. 157: 1 → 0
Char. 158: 1 → 0
Char. 159: 2 → 1
Char. 162: 1 → 0
Char. 165: 1 → 0
Char. 178: 0 → 1
Char. 180: 0 → 1
Char. 191: 1 → 0
Char. 194: 1 → 0
Char. 206: 2 → 1
Char. 289: 0 → 1
Char. 298: 2 → 1
Char. 301: 1 → 0
Char. 302: 0 → 1
Char. 308: 1 → 0
Char. 332: 1 → 0
Char. 353: 2 → 1
Char. 357: 1 → 0

*Galvesaurus herreroi*
Char. 130: 2 → 0
Char. 133: 0 → 1
Char. 141: 1 → 2
Char. 142: 1 → 0
Char. 159: 2 → 1
Char. 167: 0 → 1
Char. 206: 2 → 1
Char. 207: 1 → 0
Char. 250: 0 → 1
Char. 306: 1 → 0
Char. 425: 0 → 2

*Europasaurus holgeri*
Char. 18: 1 → 0
Char. 38: 1 → 0
Char. 45: 0 → 1
Char. 46: 0 → 1
Char. 79: 0 → 1
Char. 131: 0 → 1
Char. 135: 0 → 1
Char. 137: 0 → 1
Char. 193: 1 → 0
Char. 202: 0 → 1
Char. 216: 1 → 0
Char. 239: 1 → 0
Char. 278: 0 → 1
Char. 362: 1 → 0
Char. 415: 0 → 1

*Brachiosaurus altithorax*
Char. 37: 0 → 1
Char. 44: 0 → 1
Char. 125: 1 → 2
Char. 168: 12 → 0
Char. 169: 1 → 0
Char. 172: 0 → 1
Char. 179: 0 → 1
Char. 192: 0 → 1
Char. 194: 1 → 2
Char. 203: 0 → 1
Char. 205: 2 → 1
Char. 304: 1 → 0
Char. 350: 0 → 1
Char. 360: 1 → 0

*Giraffatitan brancai*
Char. 27: 0 → 1
Char. 125: 1 → 2
Char. 169: 1 → 0
Char. 172: 0 → 1
Char. 176: 0 → 2
Char. 179: 0 → 1
Char. 183: 0 → 1
Char. 194: 1 → 2
Char. 203: 0 → 1
Char. 205: 2 → 1
Char. 210: 0 → 1
Char. 255: 1 → 2
Char. 275: 3 → 1
Char. 280: 0 → 1
Char. 281: 1 → 0
Char. 298: 2 → 3
Char. 367: 0 → 1
Char. 389: 1 → 0
Char. 414: 0 → 1
Char. 415: 0 → 1

*Abydosaurus mcintoshi*
Char. 12: 1 → 0
Char. 62: 1 → 0
Char. 77: 1 → 0
Char. 92: 1 → 0
Char. 104: 0 → 1
Char. 140: 1 → 0

*Venenosaurus dicrocei*
Char. 210: 0 → 1
Char. 255: 1 → 0

*Cedarosaurus weiskopfae*
Char. 239: 1 → 0
Char. 250: 0 → 1
Char. 255: 1 → 0
Char. 258: 1 → 0
Char. 259: 0 → 1
Char. 286: 0 → 1
Char. 298: 2 → 3
Char. 301: 1 → 0
Char. 356: 0 → 1
Char. 369: 0 → 1
Char. 399: 1 → 0

*Sauroposeidon proteles*
Char. 107: 2 → 1
Char. 108: 2 → 1
Char. 111: 1 → 0
Char. 122: 0 → 1
Char. 124: 3 → 2
Char. 125: 1 → 2
Char. 126: 0 → 1
Char. 132: 1 → 0
Char. 141: 0 → 1
Char. 146: 1 → 0
Char. 148: 1 → 0
Char. 161: 0 → 1
Char. 168: 2 → 1

Char. 170: 1 → 0
Char. 176: 0 → 1
Char. 177: 1 → 0
Char. 178: 2 → 0
Char. 196: 0 → 1
Char. 205: 2 → 1
Char. 212: 4 → 2
Char. 229: 3 → 1
Char. 241: 2 → 0
Char. 250: 3 → 0
Char. 255: 1 → 2
Char. 268: 1 → 0
Char. 273: 1 → 0
Char. 283: 1 → 0
Char. 290: 1 → 0
Char. 291: 1 → 0
Char. 297: 1 → 0
Char. 298: 2 → 1
Char. 302: 1 → 0
Char. 312: 1 → 0
Char. 330: 1 → 0
Char. 339: 1 → 0
Char. 346: 1 → 0
Char. 421: 1 → 0

*Tastavinsaurus sanzi*

Char. 162: 1 → 0
Char. 200: 1 → 0
Char. 204: 0 → 1
Char. 226: 1 → 0
Char. 235: 0 → 1
Char. 254: 0 → 1
Char. 258: 1 → 0
Char. 259: 0 → 1
Char. 270: 0 → 1
Char. 349: 0 → 1
Char. 353: 2 → 1
Char. 369: 0 → 1
Char. 384: 0 → 1
Char. 387: 1 → 0
Char. 396: 1 → 0

*Euhelopus zdanskyi*

Char. 1: 0 → 1
Char. 16: 0 → 1
Char. 24: 1 → 0
Char. 25: 1 → 0
Char. 86: 1 → 0
Char. 99: 2 → 1
Char. 104: 0 → 1
Char. 107: 2 → 1
Char. 108: 2 → 0
Char. 111: 1 → 0
Char. 113: 1 → 0
Char. 118: 3 → 7
Char. 120: 1 → 2
Char. 126: 0 → 1
Char. 127: 0 → 1
Char. 130: 2 → 0
Char. 131: 0 → 1
Char. 132: 1 → 0
Char. 133: 0 → 1
Char. 140: 1 → 0
Char. 144: 0 → 1
Char. 150: 0 → 1
Char. 153: 4 → 1
Char. 156: 0 → 1
Char. 161: 0 → 1
Char. 163: 2 → 1
Char. 164: 1 → 0
Char. 168: 2 → 1
Char. 169: 2 → 1
Char. 175: 2 → 3
Char. 176: 0 → 1
Char. 184: 0 → 1
Char. 193: 1 → 0
Char. 194: 1 → 2
Char. 196: 0 → 1
Char. 212: 4 → 3
Char. 273: 1 → 0
Char. 281: 0 → 1
Char. 284: 1 → 0
Char. 288: 1 → 0
Char. 297: 1 → 0
Char. 305: 2 → 0
Char. 329: 1 → 0
Char. 332: 1 → 0
Char. 339: 1 → 0
Char. 346: 1 → 0
Char. 348: 0 → 1
Char. 353: 2 → 1
Char. 356: 1 → 0
Char. 360: 1 → 0
Char. 369: 0 → 1
Char. 389: 1 → 0
Char. 412: 1 → 0
Char. 436: 1 → 0
Char. 439: 1 → 0

*Phuwiangosaurus sirindhornae*

Char. 26: 0 → 1
Char. 71: 0 → 1
Char. 72: 0 → 1
Char. 77: 1 → 0
Char. 108: 2 → 1
Char. 120: 1 → 2
Char. 124: 3 → 2
Char. 126: 0 → 1
Char. 127: 0 → 1
Char. 128: 0 → 1
Char. 129: 0 → 1
Char. 130: 2 → 1
Char. 132: 1 → 0
Char. 136: 0 → 1
Char. 141: 0 → 1
Char. 142: 1 → 0
Char. 144: 0 → 1
Char. 146: 1 → 0
Char. 148: 1 → 0
Char. 150: 0 → 1
Char. 153: 4 → 2
Char. 156: 0 → 1
Char. 161: 0 → 1
Char. 163: 2 → 1
Char. 164: 1 → 0
Char. 168: 2 → 1
Char. 169: 2 → 1
Char. 175: 2 → 1
Char. 176: 0 → 1
Char. 177: 1 → 0
Char. 178: 2 → 1
Char. 184: 0 → 1
Char. 194: 1 → 2
Char. 207: 1 → 0
Char. 208: 0 → 1
Char. 212: 4 → 2
Char. 217: 0 → 1

Char. 229: 3 → 1
Char. 231: 1 → 0
Char. 235: 1 → 0
Char. 239: 0 → 1
Char. 241: 2 → 0
Char. 248: 0 → 1
Char. 250: 3 → 0
Char. 254: 0 → 1
Char. 268: 1 → 0
Char. 273: 1 → 0
Char. 297: 1 → 0
Char. 302: 1 → 0
Char. 305: 2 → 0
Char. 309: 1 → 0
Char. 310: 0 → 1
Char. 328: 0 → 2
Char. 330: 1 → 0
Char. 339: 1 → 0
Char. 346: 1 → 0
Char. 350: 0 → 1
Char. 356: 1 → 0
Char. 363: 0 → 1
Char. 369: 0 → 1
Char. 412: 1 → 0
Char. 421: 1 → 0
Char. 428: 2 → 0

*Qiaowanlong kangxii*
Char. 124: 3 → 2
Char. 126: 0 → 1
Char. 127: 0 → 1
Char. 128: 0 → 1
Char. 129: 0 → 1
Char. 132: 1 → 0
Char. 136: 0 → 1
Char. 141: 0 → 1
Char. 144: 0 → 1
Char. 146: 1 → 0
Char. 335: 1 → 0
Char. 339: 1 → 0
Char. 346: 1 → 0
Char. 421: 1 → 0

*Ruyangosaurus giganteus*
Char. 120: 1 → 0
Char. 130: 2 → 3
Char. 142: 1 → 0
Char. 146: 1 → 0
Char. 148: 1 → 0
Char. 153: 4 → 1
Char. 156: 0 → 1
Char. 161: 0 → 1
Char. 163: 2 → 1
Char. 164: 1 → 0
Char. 168: 2 → 1
Char. 175: 2 → 3
Char. 176: 0 → 1
Char. 177: 1 → 0
Char. 178: 2 → 1
Char. 180: 0 → 1
Char. 181: 1 → 0
Char. 183: 1 → 0
Char. 216: 1 → 0
Char. 250: 3 → 0
Char. 273: 1 → 0
Char. 278: 0 → 1
Char. 279: 1 → 0
Char. 281: 0 → 1
Char. 283: 1 → 0
Char. 297: 1 → 0
Char. 305: 2 → 1
Char. 328: 0 → 1
Char. 330: 1 → 0
Char. 348: 0 → 1
Char. 356: 1 → 0
Char. 359: 0 → 1
Char. 423: 1 → 0
Char. 425: 0 → 2
Char. 426: 0 → 1
Char. 427: 1 → 0

*Erketu ellisoni*
Char. 120: 1 → 2
Char. 126: 0 → 1
Char. 128: 0 → 1
Char. 129: 0 → 1
Char. 130: 2 → 3
Char. 132: 1 → 0
Char. 136: 0 → 1
Char. 141: 0 → 1
Char. 146: 1 → 0
Char. 363: 0 → 1
Char. 375: 0 → 1
Char. 421: 1 → 0
Char. 435: 1 → 0
Char. 438: 0 → 1
Char. 443: 0 → 1

*Chubutisaurus insignis*
Char. 161: 0 → 1
Char. 162: 1 → 0
Char. 164: 1 → 0
Char. 169: 2 → 1
Char. 176: 0 → 1
Char. 177: 1 → 0
Char. 200: 1 → 0
Char. 205: 2 → 1
Char. 222: 1 → 0
Char. 223: 2 → 0
Char. 229: 3 → 1
Char. 231: 1 → 0
Char. 250: 3 → 0
Char. 268: 1 → 0
Char. 281: 0 → 1
Char. 283: 1 → 0
Char. 302: 1 → 0
Char. 305: 2 → 1
Char. 339: 1 → 0
Char. 345: 0 → 1
Char. 346: 1 → 0
Char. 418: 02 → 1
Char. 428: 2 → 0

*Ligabuesaurus leanzai*
Char. 108: 2 → 1
Char. 120: 1 → 0
Char. 132: 1 → 0
Char. 142: 1 → 0
Char. 146: 1 → 0
Char. 148: 1 → 2
Char. 151: 0 → 1
Char. 157: 13 → 0
Char. 163: 2 → 1
Char. 168: 2 → 1
Char. 169: 2 → 1
Char. 170: 1 → 0
Char. 171: 0 → 2
Char. 172: 0 → 1

Char. 173: 1 → 2
Char. 175: 2 → 0
Char. 176: 0 → 1
Char. 177: 1 → 0
Char. 178: 2 → 0
Char. 281: 0 → 1
Char. 297: 1 → 0
Char. 302: 1 → 0
Char. 304: 1 → 0
Char. 305: 2 → 1
Char. 384: 0 → 1
Char. 421: 1 → 2

*Wintonotitan wattsi*
Char. 229: 3 → 1
Char. 231: 1 → 0
Char. 232: 0 → 1
Char. 250: 3 → 0
Char. 259: 023 → 1
Char. 268: 1 → 0
Char. 281: 0 → 1
Char. 302: 1 → 0

*Andesaurus delgadoi*
Char. 161: 0 → 1
Char. 168: 2 → 1
Char. 169: 2 → 1
Char. 177: 1 → 0
Char. 178: 2 → 0
Char. 205: 2 → 1
Char. 206: 2 → 0
Char. 222: 1 → 0
Char. 224: 0 → 1
Char. 229: 3 → 2
Char. 232: 0 → 1
Char. 235: 1 → 0
Char. 241: 2 → 0
Char. 250: 3 → 0
Char. 255: 1 → 2
Char. 339: 1 → 0

*Malawisaurus dixeyi*
Char. 0: 0 → 1
Char. 1: 0 → 1
Char. 46: 0 → 2
Char. 68: 0 → 1
Char. 86: 1 → 0
Char. 118: 3 → 2
Char. 126: 0 → 1
Char. 128: 0 → 1
Char. 130: 2 → 3
Char. 132: 1 → 0
Char. 147: 1 → 2
Char. 153: 4 → 3
Char. 170: 1 → 0
Char. 171: 0 → 1
Char. 172: 0 → 1
Char. 174: 23 → 0
Char. 192: 1 → 0
Char. 218: 0 → 1
Char. 220: 0 → 1
Char. 224: 0 → 1
Char. 225: 1 → 0
Char. 229: 3 → 2
Char. 231: 1 → 0
Char. 232: 0 → 1
Char. 235: 1 → 0
Char. 241: 2 → 0
Char. 250: 3 → 1
Char. 258: 1 → 0
Char. 288: 1 → 0
Char. 304: 1 → 0
Char. 305: 2 → 1
Char. 308: 1 → 0
Char. 309: 1 → 0
Char. 341: 0 → 1
Char. 363: 0 → 1
Char. 421: 1 → 2

*Paludititan nalatzensis*
Char. 155: 0 → 1
Char. 201: 1 → 0
Char. 205: 2 → 1
Char. 235: 1 → 0
Char. 241: 2 → 0
Char. 250: 3 → 01
Char. 258: 1 → 0
Char. 326: 1 → 0
Char. 335: 1 → 0
Char. 346: 1 → 0
Char. 427: 1 → 0

*Tapuiasaurus macedoi*
Char. 7: 0 → 1
Char. 8: 0 → 1
Char. 13: 1 → 2
Char. 21: 1 → 2
Char. 25: 1 → 2
Char. 35: 0 → 1
Char. 46: 0 → 2
Char. 63: 0 → 1
Char. 68: 0 → 1
Char. 79: 0 → 1
Char. 92: 1 → 0
Char. 118: 3 → 4
Char. 119: 0 → 1
Char. 122: 0 → 1
Char. 130: 2 → 1
Char. 147: 1 → 2
Char. 168: 2 → 1
Char. 181: 1 → 0
Char. 183: 1 → 0
Char. 187: 0 → 2
Char. 192: 1 → 0
Char. 193: 1 → 0
Char. 196: 0 → 1
Char. 205: 2 → 1
Char. 207: 1 → 0
Char. 229: 3 → 1
Char. 250: 3 → 2
Char. 259: 023 → 1
Char. 289: 0 → 1
Char. 296: 0 → 1
Char. 305: 2 → 0
Char. 309: 1 → 0
Char. 313: 0 → 1
Char. 369: 0 → 1
Char. 383: 0 → 2
Char. 384: 0 → 1
Char. 406: 0 → 1
Char. 409: 2 → 3
Char. 421: 1 → 23
Char. 427: 1 → 0
Char. 432: 2 → 0
Char. 435: 1 → 0
Char. 438: 0 → 1
Char. 442: 1 → 0
Char. 443: 0 → 1

Char. 445: 1 → 0
Char. 452: 2 → 1

*Patagotitan mayorum*
Char. 183: 1 → 0

*Argentinosaurus huinculensis*
Char. 160: 1 → 0
Char. 163: 2 → 1
Char. 174: 3 → 1
Char. 176: 0 → 1
Char. 424: 2 → 0

*Futalognkosaurus dukei*
Char. 234: 0 → 1
Char. 245: 0 → 1
Char. 350: 0 → 1

*Mendozasaurus neguyelap*
Char. 122: 0 → 1

*Austroposeidon magnificus*
Char. 148: 2 → 1

BIBE 45854
Char. 137: 0 → 1

*Kaijutitan maui*
Char. 210: 1 → 0
Char. 362: 1 → 0

*Bonitasaura salgadoi*
No autapomorphies

*Epachthosaurus sciuttoi*
Char. 161: 0 → 1
Char. 177: 1 → 0
Char. 178: 2 → 0
Char. 183: 1 → 2
Char. 193: 1 → 2
Char. 196: 0 → 1
Char. 206: 2 → 1
Char. 215: 0 → 1
Char. 220: 0 → 1
Char. 231: 1 → 2
Char. 235: 1 → 0
Char. 239: 0 → 1
Char. 242: 0 → 1
Char. 249: 0 → 1
Char. 258: 1 → 0
Char. 270: 0 → 1
Char. 298: 2 → 3
Char. 304: 1 → 0
Char. 309: 1 → 0
Char. 369: 0 → 1
Char. 370: 0 → 1
Char. 383: 0 → 1
Char. 402: 2 → 1
Char. 416: 0 → 1

*Notocolossus gonzalezparejasi*
Char. 160: 1 → 0
Char. 173: 1 → 0
Char. 244: 1 → 0
Char. 422: 1 → 2
Char. 424: 2 → 0

*Puertasaurus reuili*
Char. 120: 1 → 2
Char. 173: 1 → 0
Char. 422: 1 → 2
Char. 424: 2 → 0

*Muyelensaurus pecheni*
Char. 67: 1 → 0
Char. 142: 1 → 0
Char. 160: 1 → 0
Char. 173: 1 → 0
Char. 194: 1 → 0
Char. 203: 0 → 1
Char. 248: 0 → 3
Char. 260: 0 → 1
Char. 280: 1 → 0
Char. 302: 1 → 0
Char. 335: 1 → 0
Char. 409: 2 → 1
Char. 417: 0 → 1
Char. 418: 02 → 1
Char. 424: 2 → 0
Char. 425: 0 → 1
Char. 426: 0 → 1
Char. 427: 1 → 0
Char. 430: 0 → 1

*Maxakalisaurus topai*
Char. 110: 0 → 1
Char. 142: 1 → 0
Char. 144: 0 → 1
Char. 151: 0 → 1
Char. 155: 0 → 1
Char. 169: 2 → 1
Char. 181: 1 → 0
Char. 183: 1 → 2
Char. 194: 1 → 0
Char. 203: 0 → 1
Char. 208: 0 → 1
Char. 248: 0 → 3
Char. 260: 0 → 1
Char. 302: 1 → 0
Char. 409: 2 → 3
Char. 416: 0 → 1
Char. 424: 2 → 0

*Adamantisaurus mezzalirai*
Char. 176: 0 → 1
Char. 224: 0 → 1
Char. 228: 0 → 1
Char. 248: 0 → 3
Char. 252: 0 → 1
Char. 253: 0 → 1
Char. 260: 0 → 1
Char. 286: 0 → 1
Char. 289: 0 → 1
Char. 304: 1 → 0
Char. 359: 0 → 1
Char. 411: 1 → 0
Char. 416: 0 → 1
Char. 419: 0 → 1
Char. 429: 0 → 2
Char. 430: 0 → 1
Char. 431: 1 → 0

*Arrudatitan maximus*
Char. 126: 0 → 1
Char. 144: 0 → 1
Char. 169: 2 → 1
Char. 181: 1 → 0
Char. 194: 1 → 0
Char. 225: 1 → 0
Char. 228: 0 → 1

Char. 248: 0 → 3
Char. 252: 0 → 1
Char. 253: 0 → 1
Char. 260: 0 → 1
Char. 359: 0 → 1
Char. 409: 2 → 3
Char. 411: 1 → 0
Char. 416: 0 → 1
Char. 419: 0 → 1
Char. 429: 0 → 1
Char. 430: 0 → 1
Char. 431: 1 → 0

*Rinconsaurus caudamirus*
Char. 139: 1 → 2
Char. 142: 1 → 0
Char. 169: 2 → 1
Char. 180: 0 → 1
Char. 183: 1 → 2
Char. 231: 1 → 2
Char. 248: 0 → 3
Char. 255: 1 → 2
Char. 260: 0 → 1
Char. 277: 0 → 1
Char. 286: 0 → 1
Char. 289: 0 → 1
Char. 302: 1 → 0
Char. 409: 2 → 1
Char. 416: 0 → 1
Char. 418: 02 → 1
Char. 424: 2 → 01
Char. 425: 0 → 1
Char. 429: 0 → 1
Char. 430: 0 → 1

*Pitekunsaurus macayai*
Char. 38: 1 → 0
Char. 67: 1 → 2
Char. 76: 1 → 0
Char. 130: 2 → 3
Char. 141: 0 → 1
Char. 149: 1 → 0
Char. 160: 1 → 0
Char. 173: 1 → 0
Char. 231: 1 → 2
Char. 246: 0 → 1
Char. 248: 0 → 3
Char. 260: 0 → 1
Char. 277: 0 → 1
Char. 280: 1 → 0
Char. 409: 2 → 1
Char. 418: 02 → 1
Char. 424: 2 → 0
Char. 429: 0 → 1
Char. 430: 0 → 1
Char. 439: 1 → 0
Char. 448: 0 → 1

*Brasilotitan nemophagus*
Char. 85: 0 → 1
Char. 98: 1 → 2
Char. 126: 0 → 1
Char. 128: 0 → 1
Char. 130: 2 → 3
Char. 133: 0 → 1
Char. 139: 1 → 2
Char. 144: 0 → 1
Char. 149: 1 → 0

*Uberabatitan ribeiroi*
Char. 120: 1 → 2
Char. 126: 0 → 1
Char. 128: 0 → 1
Char. 130: 2 → 3
Char. 133: 0 → 1
Char. 139: 1 → 2
Char. 147: 1 → 2
Char. 148: 1 → 0
Char. 149: 1 → 0
Char. 151: 0 → 1
Char. 181: 1 → 0
Char. 193: 1 → 2
Char. 196: 0 → 1
Char. 203: 0 → 1
Char. 231: 1 → 2
Char. 246: 0 → 1
Char. 248: 0 → 3
Char. 252: 0 → 1
Char. 253: 0 → 1
Char. 258: 1 → 2
Char. 270: 0 → 1
Char. 288: 1 → 0
Char. 289: 0 → 1
Char. 313: 0 → 1
Char. 349: 0 → 1
Char. 350: 0 → 1
Char. 370: 0 → 1
Char. 372: 0 → 1
Char. 409: 2 → 3
Char. 416: 0 → 1
Char. 424: 2 → 0
Char. 429: 0 → 1
Char. 430: 0 → 1

*Overosaurus paradasorum*
Char. 120: 1 → 2
Char. 128: 0 → 1
Char. 130: 2 → 3
Char. 139: 1 → 2
Char. 144: 0 → 1
Char. 149: 1 → 0
Char. 158: 1 → 3
Char. 160: 1 → 0
Char. 173: 1 → 0
Char. 193: 1 → 2
Char. 194: 1 → 0
Char. 196: 0 → 1
Char. 221: 1 → 0
Char. 232: 0 → 1
Char. 248: 0 → 3
Char. 252: 0 → 1
Char. 409: 2 → 1
Char. 418: 02 → 1
Char. 424: 2 → 0

*Trigonosaurus pricei*
Char. 120: 1 → 2
Char. 128: 0 → 1
Char. 130: 2 → 3
Char. 139: 1 → 2
Char. 144: 0 → 1
Char. 149: 1 → 0
Char. 158: 1 → 3
Char. 160: 1 → 0
Char. 173: 1 → 0
Char. 180: 0 → 1
Char. 193: 1 → 2
Char. 194: 1 → 0

Char. 196: 0 → 1
Char. 203: 0 → 1
Char. 225: 1 → 0
Char. 232: 0 → 1
Char. 234: 0 → 1
Char. 248: 0 → 3
Char. 252: 0 → 1
Char. 409: 2 → 1
Char. 424: 2 → 0
Char. 425: 0 → 1
Char. 426: 0 → 1
Char. 429: 0 → 2
Char. 430: 0 → 1
Char. 431: 1 → 0

*Punatitan coughlini*
Char. 183: 1 → 2
Char. 193: 1 → 0
Char. 201: 1 → 0
Char. 203: 0 → 1
Char. 221: 1 → 0
Char. 232: 0 → 1
Char. 248: 0 → 3
Char. 249: 0 → 1
Char. 252: 0 → 2
Char. 253: 0 → 1
Char. 255: 1 → 0
Char. 409: 2 → 1
Char. 411: 1 → 0
Char. 419: 0 → 1
Char. 427: 1 → 0
Char. 430: 0 → 1

*Aeolosaurus rionegrinus*
Char. 248: 0 → 3
Char. 249: 0 → 1
Char. 251: 1 → 2
Char. 252: 0 → 2
Char. 253: 0 → 1
Char. 302: 1 → 0
Char. 313: 0 → 1
Char. 367: 0 → 1
Char. 411: 1 → 0
Char. 419: 0 → 1

*Gondwanatitan faustoi*
Char. 169: 2 → 1
Char. 183: 1 → 2
Char. 248: 0 → 3
Char. 249: 0 → 1
Char. 251: 1 → 2
Char. 252: 0 → 2
Char. 253: 0 → 1
Char. 255: 1 → 2
Char. 258: 1 → 2
Char. 302: 1 → 0
Char. 409: 2 → 1
Char. 411: 1 → 0

*Dreadnoughtus schrani*
Char. 122: 0 → 1
Char. 130: 2 → 1
Char. 142: 1 → 0
Char. 148: 1 → 0
Char. 168: 2 → 1
Char. 169: 2 → 0
Char. 176: 0 → 1
Char. 194: 1 → 0
Char. 225: 1 → 0
Char. 231: 1 → 0
Char. 246: 0 → 1
Char. 255: 1 → 2
Char. 260: 0 → 1
Char. 274: 1 → 0
Char. 275: 3 → 0
Char. 288: 1 → 0
Char. 289: 0 → 1
Char. 302: 1 → 2
Char. 308: 1 → 0
Char. 310: 0 → 1
Char. 312: 1 → 0
Char. 313: 0 → 1
Char. 328: 0 → 2
Char. 335: 1 → 0
Char. 349: 0 → 1
Char. 369: 0 → 1
Char. 412: 1 → 0
Char. 421: 1 → 0

*Diamantinasaurus matildae*
Char. 46: 0 → 2
Char. 68: 0 → 1
Char. 120: 1 → 0
Char. 124: 3 → 2
Char. 125: 1 → 0
Char. 132: 1 → 0
Char. 167: 1 → 0
Char. 168: 2 → 1
Char. 169: 2 → 0
Char. 170: 1 → 0
Char. 172: 0 → 1
Char. 176: 0 → 2
Char. 183: 1 → 0
Char. 184: 0 → 1
Char. 187: 0 → 2
Char. 273: 1 → 0
Char. 275: 3 → 0
Char. 279: 1 → 0
Char. 283: 1 → 0
Char. 298: 2 → 1
Char. 302: 1 → 2
Char. 305: 2 → 0
Char. 309: 1 → 0
Char. 310: 0 → 1
Char. 313: 0 → 1
Char. 320: 1 → 0
Char. 321: 1 → 0
Char. 322: 2 → 1
Char. 341: 0 → 1
Char. 357: 1 → 0
Char. 358: 0 → 1
Char. 363: 0 → 1
Char. 423: 1 → 0
Char. 425: 0 → 1
Char. 435: 1 → 2
Char. 440: 0 → 2
Char. 442: 1 → 0
Char. 445: 1 → 0
Char. 446: 0 → 1
Char. 450: 0 → 1

*Mansourasaurus shahinae*
Char. 85: 0 → 1
Char. 124: 3 → 2
Char. 125: 1 → 0
Char. 132: 1 → 0
Char. 283: 1 → 0

Char. 289: 0 → 1
Char. 295: 0 → 1
Char. 300: 0 → 1
Char. 302: 1 → 2
Char. 303: 0 → 1
Char. 305: 2 → 0
Char. 313: 0 → 1
Char. 384: 0 → 1

*Lohuecotitan pandafilandi*
Char. 122: 0 → 1
Char. 124: 3 → 1
Char. 130: 2 → 1
Char. 132: 1 → 0
Char. 155: 0 → 1
Char. 156: 0 → 1
Char. 163: 2 → 1
Char. 167: 1 → 0
Char. 172: 0 → 1
Char. 221: 1 → 2
Char. 225: 1 → 0
Char. 240: 0 → 1
Char. 243: 0 → 1
Char. 248: 0 → 1
Char. 250: 3 → 2
Char. 259: 023 → 1
Char. 260: 0 → 1
Char. 330: 1 → 0
Char. 331: 1 → 0
Char. 332: 1 → 0
Char. 353: 2 → 1
Char. 357: 1 → 0
Char. 358: 0 → 1
Char. 362: 1 → 0
Char. 365: 1 → 0
Char. 367: 0 → 1
Char. 368: 1 → 0
Char. 423: 1 → 0

*Lirainosaurus astibiae*
Char. 75: 1 → 0
Char. 76: 1 → 0
Char. 107: 2 → 3
Char. 110: 0 → 1
Char. 112: 0 → 1
Char. 155: 0 → 1
Char. 156: 0 → 1
Char. 158: 1 → 0
Char. 176: 0 → 2
Char. 191: 1 → 0
Char. 193: 1 → 0
Char. 194: 1 → 0
Char. 200: 1 → 0
Char. 203: 0 → 1
Char. 204: 0 → 1
Char. 219: 0 → 1
Char. 225: 1 → 0
Char. 235: 1 → 0
Char. 241: 2 → 1
Char. 248: 0 → 1
Char. 255: 1 → 2
Char. 259: 023 → 1
Char. 260: 0 → 1
Char. 274: 1 → 0
Char. 280: 1 → 0
Char. 283: 1 → 0
Char. 286: 0 → 1
Char. 288: 1 → 0
Char. 298: 2 → 1
Char. 300: 0 → 1
Char. 305: 2 → 1
Char. 309: 1 → 0
Char. 349: 0 → 1
Char. 357: 1 → 0
Char. 358: 0 → 1
Char. 359: 0 → 1
Char. 361: 1 → 0
Char. 362: 1 → 0
Char. 368: 1 → 0
Char. 409: 2 → 3
Char. 423: 1 → 0
Char. 424: 2 → 1
Char. 427: 1 → 0

*Atsinganosaurus velauciensis*
Char. 107: 2 → 3
Char. 110: 0 → 1
Char. 115: 1 → 0
Char. 120: 1 → 0
Char. 124: 3 → 0
Char. 132: 1 → 0
Char. 148: 1 → 0
Char. 217: 0 → 1
Char. 219: 0 → 1
Char. 235: 1 → 0
Char. 241: 2 → 0
Char. 244: 1 → 0
Char. 250: 3 → 1
Char. 258: 1 → 2
Char. 280: 1 → 0
Char. 286: 0 → 1
Char. 298: 2 → 1
Char. 300: 0 → 1
Char. 305: 2 → 1
Char. 309: 1 → 0
Char. 351: 1 → 2
Char. 361: 1 → 0
Char. 362: 1 → 0
Char. 381: 1 → 0

*Ampelosaurus atacis*
Char. 36: 1 → 0
Char. 67: 1 → 2
Char. 84: 1 → 0
Char. 106: 0 → 1
Char. 107: 2 → 1
Char. 108: 2 → 1
Char. 110: 0 → 1
Char. 113: 1 → 0
Char. 120: 1 → 0
Char. 124: 3 → 0
Char. 131: 0 → 1
Char. 132: 1 → 0
Char. 149: 1 → 0
Char. 155: 0 → 1
Char. 156: 0 → 1
Char. 158: 1 → 0
Char. 163: 2 → 1
Char. 167: 1 → 0
Char. 172: 0 → 1
Char. 176: 0 → 2
Char. 183: 1 → 2
Char. 187: 0 → 12
Char. 194: 1 → 0
Char. 231: 1 → 0
Char. 257: 0 → 1
Char. 259: 023 → 1
Char. 274: 1 → 0

Char. 280: 1 → 0
Char. 286: 0 → 1
Char. 298: 2 → 1
Char. 300: 0 → 1
Char. 305: 2 → 1
Char. 335: 1 → 0
Char. 346: 1 → 0
Char. 351: 1 → 2
Char. 423: 1 → 0
Char. 427: 1 → 0

*Rapetosaurus krausei*
Char. 252: 0 → 1
Char. 258: 1 → 2
Char. 330: 1 → 0
Char. 418: 0 → 1
Char. 423: 0 → 2
Char. 425: 0 → 1

*Isisaurus colberti*
Char. 137: 0 → 1
Char. 183: 2 → 0
Char. 193: 2 → 0
Char. 207: 1 → 0
Char. 241: 2 → 0
Char. 272: 1 → 0
Char. 327: 1 → 0
Char. 345: 0 → 1

*Nemegtosaurus mongoliensis*
Char. 159: 3 → 2

*Opisthocoelicaudia skarzynskii*
Char. 427: 1 → 0

*Alamosaurus sanjuanensis*
Char. 217: 0 → 1
Char. 224: 0 → 1
Char. 231: 1 → 0

*Baurutitan britoi*
Char. 234: 0 → 1
Char. 416: 0 → 1

*Bonatitan reigi*
Char. 124: 3 → 1
Char. 126: 1 → 0
Char. 358: 1 → 0

*Bravasaurus arrierosorum*
Char. 120: 1 → 2
Char. 128: 0 → 1
Char. 130: 2 → 3
Char. 139: 1 → 2
Char. 142: 1 → 0
Char. 144: 0 → 1
Char. 147: 1 → 2
Char. 180: 0 → 1
Char. 183: 1 → 2
Char. 187: 0 → 2
Char. 193: 1 → 2
Char. 194: 1 → 0
Char. 196: 0 → 1
Char. 203: 0 → 1
Char. 300: 0 → 1
Char. 305: 2 → 1
Char. 350: 0 → 1
Char. 367: 0 → 1
Char. 420: 0 → 3
Char. 424: 2 → 0
Char. 427: 1 → 0
Char. 430: 0 → 1

*Ibirania parva*
Char. 160: 0 → 1
Char. 161: 0 → 1
Char. 189: 0 → 1
Char. 190: 1 → 0
Char. 191: 1 → 0
Char. 201: 1 → 0
Char. 203: 0 → 1
Char. 206: 2 → 0
Char. 231: 1 → 0
Char. 426: 0 → 1

*Rocasaurus muniozi*
Char. 416: 0 → 1

MACN PV RN 233
No autapomorphies

*Neuquensaurus australis*
Char. 206: 2 → 1
Char. 369: 1 → 0
Char. 440: 2 → 1

*Saltasaurus loricatus*
No autapomorphies

Vila Ventura axis
Char. 437: 0 → 1
Char. 438: 0 → 2
Char. 443: 0 → 1
Char. 444: 0 → 1
Char. 448: 0 → 1

Node 108 - Sauropodiformes
Char. 84: 0 → 1

Node 109
No synapomorphies

Node 110
Char. 198: 0 → 1
Char. 206: 0 → 1
Char. 309: 0 → 1

Node 111 - Sauropoda
Char. 161: 0 → 1
Char. 199: 0 → 1
Char. 274: 0 → 1
Char. 275: 0 → 1
Char. 307: 0 → 1
Char. 385: 0 → 1

Node 112 - Gravisauria
Char. 130: 1 → 0
Char. 139: 0 → 1
Char. 164: 1 → 0
Char. 206: 1 → 2
Char. 301: 0 → 1
Char. 311: 0 → 1
Char. 315: 0 → 1
Char. 378: 0 → 1
Char. 391: 0 → 1
Char. 397: 0 → 1

Node 113
Char. 298: 0 → 1
Char. 351: 0 → 1
Char. 395: 0 → 1

Node 114
- Char. 132: 1 → 0
- Char. 166: 0 → 1
- Char. 334: 0 → 1
- Char. 352: 0 → 1
- Char. 357: 0 → 1
- Char. 365: 0 → 1
- Char. 366: 0 → 1
- Char. 394: 0 → 1
- Char. 398: 0 → 1

Node 115
- Char. 114: 0 → 2

Node 116 - Eusauropoda
- Char. 171: 0 → 1
- Char. 194: 0 → 1
- Char. 275: 1 → 3
- Char. 279: 0 → 1
- Char. 360: 1 → 0

Node 117 - Mamenchisauridae
- Char. 122: 0 → 1
- Char. 125: 01 → 3
- Char. 138: 0 → 1
- Char. 139: 1 → 0
- Char. 140: 0 → 1
- Char. 159: 0 → 3
- Char. 172: 1 → 0
- Char. 173: 2 → 1
- Char. 178: 0 → 1
- Char. 218: 1 → 0
- Char. 450: 1 → 0

Node 118
- Char. 18: 0 → 1
- Char. 55: 0 → 1
- Char. 123: 0 → 1
- Char. 124: 1 → 2
- Char. 154: 0 → 1
- Char. 162: 0 → 1
- Char. 165: 0 → 1
- Char. 216: 0 → 1
- Char. 323: 0 → 1
- Char. 388: 0 → 1

Node 119 - Neosauropoda
- Char. 77: 0 → 1
- Char. 169: 0 → 1
- Char. 244: 0 → 1
- Char. 361: 0 → 1
- Char. 372: 0 → 1
- Char. 374: 0 → 1

Node 120
- Char. 21: 1 → 0
- Char. 130: 0 → 1
- Char. 155: 0 → 1
- Char. 316: 0 → 1
- Char. 343: 0 → 1
- Char. 371: 0 → 1
- Char. 409: 0 → 2

Node 121 - Dicraeosaurinae
- Char. 202: 0 → 1

Node 122
- Char. 37: 0 → 1
- Char. 49: 0 → 1
- Char. 123: 1 → 0
- Char. 125: 12 → 3
- Char. 154: 1 → 0
- Char. 159: 1 → 0
- Char. 449: 0 → 1
- Char. 450: 1 → 2

Node 123 - Dicraeosauridae
- Char. 3: 0 → 1
- Char. 28: 0 → 1
- Char. 36: 1 → 0
- Char. 45: 0 → 1
- Char. 52: 0 → 1
- Char. 88: 0 → 1
- Char. 107: 3 → 2
- Char. 170: 0 → 2

Node 124 - Flagellicaudata
- Char. 13: 1 → 2
- Char. 150: 0 → 1
- Char. 189: 0 → 1
- Char. 229: 0 → 2
- Char. 234: 0 → 1
- Char. 258: 1 → 2
- Char. 274: 1 → 0
- Char. 275: 2 → 0
- Char. 333: 0 → 1
- Char. 342: 1 → 0
- Char. 343: 1 → 0
- Char. 344: 0 → 1
- Char. 384: 0 → 1
- Char. 428: 0 → 1

Node 125 - Diplodocoidea
- Char. 0: 1 → 0
- Char. 1: 1 → 0
- Char. 2: 0 → 2
- Char. 8: 0 → 1
- Char. 10: 0 → 1
- Char. 21: 0 → 2
- Char. 22: 1 → 2
- Char. 24: 0 → 1
- Char. 44: 1 → 2
- Char. 57: 0 → 1
- Char. 60: 1 → 2
- Char. 80: 0 → 1
- Char. 98: 1 → 2
- Char. 99: 1 → 3
- Char. 102: 0 → 1
- Char. 105: 1 → 0
- Char. 106: 1 → 0
- Char. 107: 1 → 3
- Char. 108: 0 → 3
- Char. 110: 0 → 1
- Char. 113: 0 → 1
- Char. 122: 0 → 1
- Char. 145: 1 → 0
- Char. 159: 0 → 1
- Char. 162: 1 → 0
- Char. 165: 1 → 2
- Char. 194: 1 → 0
- Char. 208: 0 → 1
- Char. 215: 0 → 1
- Char. 235: 0 → 3
- Char. 241: 0 → 1
- Char. 257: 0 → 1
- Char. 261: 0 → 1
- Char. 262: 0 → 2
- Char. 275: 3 → 2

Node 126 - Diplodocidae

Char. 27: 0 → 1
Char. 29: 0 → 1
Char. 30: 0 → 1
Char. 46: 0 → 1
Char. 109: 1 → 0
Char. 134: 1 → 2
Char. 153: 2 → 4
Char. 155: 1 → 0
Char. 180: 0 → 1
Char. 192: 0 → 1
Char. 237: 0 → 1
Char. 238: 0 → 1
Char. 243: 0 → 1

Node 127 - Diplodocinae

Char. 140: 0 → 1
Char. 143: 0 → 1
Char. 230: 0 → 1
Char. 231: 0 → 1
Char. 232: 0 → 1
Char. 240: 0 → 1
Char. 248: 0 → 2
Char. 249: 0 → 1
Char. 255: 2 → 3

Node 128 - Rebbachisauridae

Char. 133: 0 → 1
Char. 239: 1 → 0
Char. 248: 0 → 1
Char. 361: 1 → 2
Char. 408: 0 → 1
Char. 409: 2 → 3

Node 129 - Nigersaurini

Char. 178: 2 → 1
Char. 189: 0 → 1

Node 130

Char. 410: 0 → 1

Node 131 - Rebbachisaurinae

Char. 192: 0 → 1
Char. 233: 0 → 1
Char. 249: 0 → 1
Char. 338: 0 → 1
Char. 407: 0 → 1

Node 132 - Khebbashia

Char. 112: 0 → 1
Char. 117: 0 → 1
Char. 178: 1 → 2
Char. 302: 1 → 0
Char. 337: 0 → 1
Char. 345: 0 → 1

Node 133

Char. 178: 0 → 1
Char. 229: 0 → 4
Char. 336: 0 → 1

Node 134

Char. 234: 0 → 1

Node 135 - Limaysaurinae

Char. 243: 0 → 1
Char. 282: 0 → 1

Node 136 - Turiasauria

Char. 108: 0 → 1
Char. 156: 0 → 1
Char. 192: 0 → 1
Char. 229: 0 → 2

Node 137 - Macronaria

Char. 2: 0 → 1
Char. 92: 0 → 1
Char. 95: 0 → 1
Char. 130: 1 → 2
Char. 155: 1 → 0
Char. 159: 0 → 1
Char. 172: 1 → 0
Char. 193: 0 → 1
Char. 281: 0 → 1
Char. 298: 1 → 2
Char. 318: 0 → 1
Char. 319: 0 → 1
Char. 335: 0 → 1
Char. 340: 0 → 1
Char. 372: 1 → 0
Char. 414: 0 → 1
Char. 442: 0 → 1

Node 138 - Titanosauriformes

Char. 1: 1 → 2
Char. 21: 0 → 1
Char. 99: 1 → 2
Char. 108: 0 → 1
Char. 122: 0 → 1
Char. 134: 1 → 2
Char. 140: 0 → 1
Char. 159: 1 → 2
Char. 171: 1 → 02
Char. 207: 0 → 1
Char. 211: 0 → 1
Char. 229: 0 → 1
Char. 255: 2 → 1
Char. 288: 0 → 1
Char. 353: 1 → 2
Char. 354: 0 → 1
Char. 414: 1 → 0

Node 139

Char. 44: 1 → 0
Char. 75: 0 → 1
Char. 76: 0 → 1
Char. 106: 1 → 0
Char. 113: 0 → 1
Char. 138: 0 → 1
Char. 227: 0 → 1
Char. 251: 0 → 1
Char. 302: 1 → 0
Char. 306: 0 → 1
Char. 320: 0 → 1
Char. 321: 0 → 1
Char. 329: 0 → 1

Node 140 - Somphospondyli

Char. 0: 1 → 0
Char. 1: 2 → 0
Char. 2: 1 → 2
Char. 14: 1 → 0
Char. 24: 0 → 1
Char. 31: 0 → 1
Char. 42: 1 → 0
Char. 44: 0 → 2
Char. 56: 0 → 1
Char. 67: 0 → 1
Char. 86: 0 → 1
Char. 107: 1 → 2
Char. 108: 1 → 2

Char. 111: 0 → 1
Char. 120: 0 → 1
Char. 122: 1 → 0
Char. 124: 2 → 3
Char. 126: 1 → 0
Char. 132: 0 → 1
Char. 138: 1 → 0
Char. 141: 1 → 0
Char. 146: 0 → 1
Char. 148: 0 → 1
Char. 153: 2 → 4
Char. 159: 2 → 3
Char. 161: 1 → 0
Char. 163: 01 → 2
Char. 164: 0 → 1
Char. 165: 1 → 0
Char. 167: 0 → 1
Char. 169: 1 → 2
Char. 170: 0 → 1
Char. 174: 0 → 23
Char. 175: 0 → 2
Char. 177: 0 → 1
Char. 178: 0 → 2
Char. 183: 0 → 1
Char. 192: 0 → 1
Char. 210: 0 → 1
Char. 212: 2 → 4
Char. 217: 1 → 0
Char. 222: 0 → 1
Char. 223: 0 → 2
Char. 226: 1 → 0
Char. 229: 1 → 3
Char. 231: 0 → 1
Char. 235: 0 → 1
Char. 239: 1 → 0
Char. 241: 0 → 2
Char. 250: 0 → 3
Char. 268: 0 → 1
Char. 273: 0 → 1
Char. 280: 0 → 1
Char. 281: 1 → 0
Char. 283: 0 → 1
Char. 287: 1 → 0
Char. 290: 0 → 1
Char. 291: 0 → 1
Char. 292: 0 → 1
Char. 294: 0 → 1
Char. 297: 0 → 1
Char. 302: 0 → 1
Char. 305: 01 → 2
Char. 312: 0 → 1
Char. 322: 1 → 2
Char. 330: 0 → 1
Char. 339: 0 → 1
Char. 346: 0 → 1
Char. 356: 0 → 1
Char. 375: 1 → 0
Char. 421: 0 → 1
Char. 423: 0 → 1
Char. 428: 0 → 2
Char. 432: 1 → 2
Char. 445: 0 → 1
Char. 452: 1 → 2

Node 141
Char. 171: 0 → 2
Char. 177: 1 → 0
Char. 178: 2 → 1
Char. 194: 1 → 2

Node 142
Char. 130: 2 → 1
Char. 133: 0 → 1
Char. 147: 1 → 2
Char. 169: 2 → 1
Char. 170: 1 → 0
Char. 172: 0 → 1
Char. 173: 1 → 2
Char. 175: 2 → 0
Char. 220: 0 → 1
Char. 235: 1 → 2
Char. 246: 0 → 1
Char. 255: 1 → 2
Char. 348: 0 → 1
Char. 350: 0 → 1
Char. 425: 0 → 2
Char. 429: 0 → 2

Node 143 - Lognkosauria
Char. 118: 3 → 4
Char. 130: 2 → 1
Char. 142: 1 → 0
Char. 148: 1 → 2
Char. 149: 1 → 0
Char. 151: 0 → 1
Char. 169: 2 → 01
Char. 170: 1 → 0
Char. 231: 1 → 0
Char. 241: 2 → 0
Char. 246: 0 → 1
Char. 383: 0 → 1
Char. 421: 1 → 3
Char. 429: 0 → 2

Node 144
Char. 132: 1 → 0
Char. 157: 3 → 0

Node 145
Char. 122: 0 → 1
Char. 126: 0 → 1
Char. 224: 0 → 1
Char. 416: 0 → 1
Char. 419: 0 → 1

Node 146
Char. 158: 1 → 3
Char. 288: 1 → 0

Node 147
Char. 13: 1 → 2
Char. 26: 0 → 1
Char. 46: 0 → 3
Char. 51: 1 → 0
Char. 68: 0 → 1
Char. 107: 2 → 3
Char. 126: 0 → 1
Char. 145: 1 → 0
Char. 148: 1 → 0
Char. 153: 4 → 3
Char. 160: 1 → 0
Char. 176: 0 → 1
Char. 183: 1 → 2
Char. 193: 1 → 2
Char. 204: 0 → 1
Char. 232: 0 → 1
Char. 249: 0 → 1

Char. 255: 1 → 2
Char. 279: 1 → 0
Char. 303: 0 → 1
Char. 309: 1 → 0
Char. 341: 0 → 1
Char. 369: 0 → 1
Char. 406: 0 → 1
Char. 423: 1 → 0

Node 148 - Opisthocoelicaudiinae
Char. 176: 1 → 2
Char. 184: 0 → 1
Char. 207: 1 → 0
Char. 229: 3 → 5
Char. 428: 2 → 0

Node 149 - Saltasauridae
Char. 67: 1 → 0
Char. 72: 0 → 1
Char. 196: 0 → 1
Char. 258: 1 → 0
Char. 286: 0 → 1
Char. 300: 0 → 1
Char. 304: 1 → 0
Char. 313: 0 → 1
Char. 349: 0 → 1
Char. 358: 0 → 1

Node 150
Char. 255: 2 → 1
Char. 418: 0 → 1
Char. 429: 0 → 1

Node 151 - Saltasaurinae
Char. 256: 0 → 1
Char. 257: 0 → 1
Char. 328: 0 → 1
Char. 347: 0 → 1
Char. 368: 1 → 0

Node 152
Char. 427: 1 → 0

Node 153
Char. 131: 0 → 1
Char. 253: 0 → 1

Node 154
Char. 219: 0 → 1
Char. 260: 0 → 1
Char. 351: 1 → 2
Char. 417: 0 → 1
Char. 425: 0 → 12

Node 155
Char. 249: 1 → 2

Node 156 - Saltasaurini
Char. 156: 0 → 1
Char. 296: 0 → 1
Char. 416: 0 → 1

## 2. Implied weighting analysis data

### 2.1. Strict consensus tree

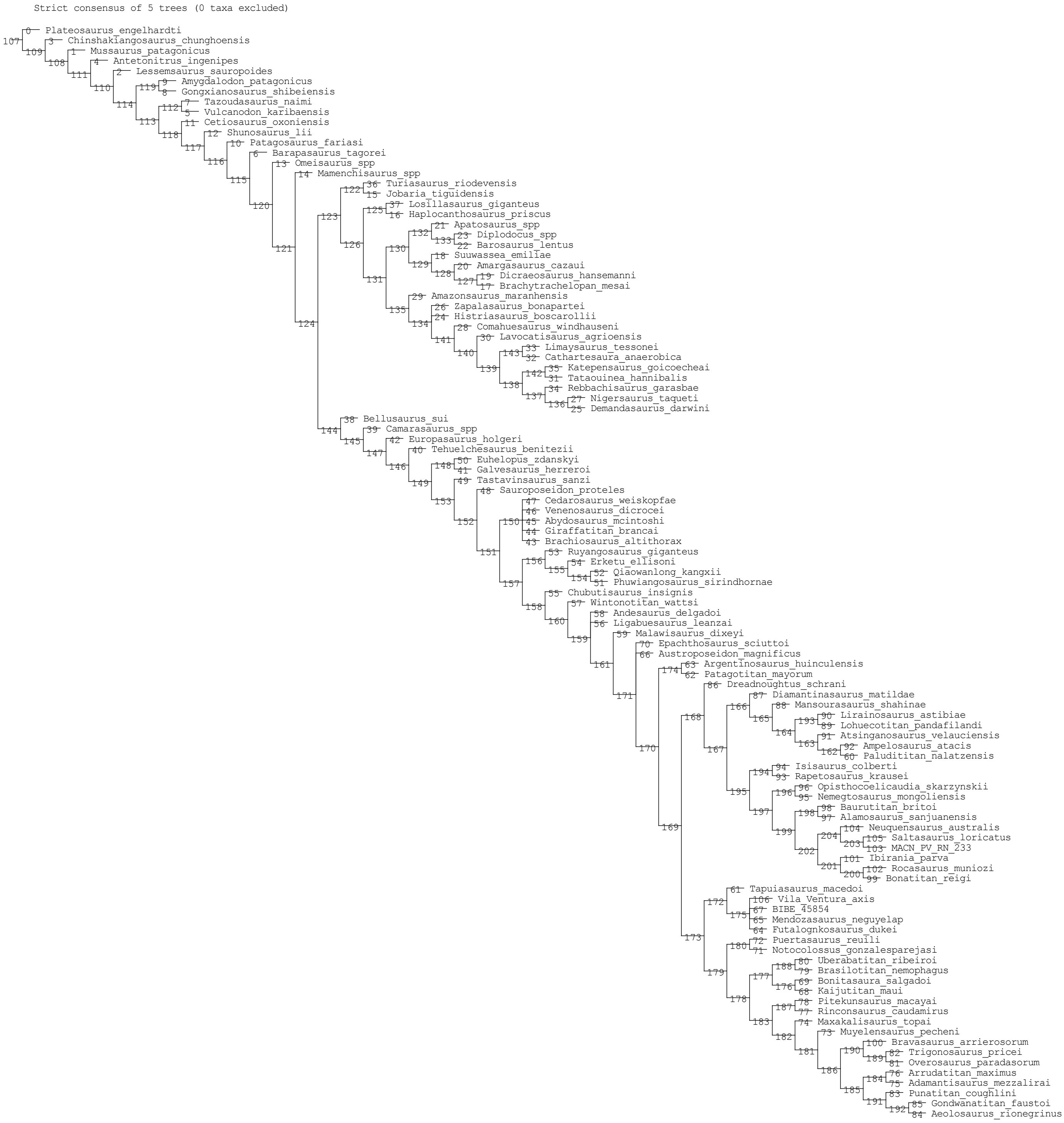

## 2.2. List of synapomorphies

*Plateosaurus engelhardti*
No autapomorphies

*Mussaurus patagonicus*
Char. 86: 0 → 1
Char. 417: 0 → 1

*Lessemsaurus sauropoides*
Char. 123: 0 → 1
Char. 390: 0 → 1

*Chinshakiangosaurus chunghoensis*
Char. 101: 0 → 1

*Antetonitrus ingenipes*
Char. 162: 0 → 1
Char. 367: 0 → 1

*Vulcanodon karibaensis*
Char. 249: 0 → 1
Char. 385: 1 → 0
Char. 395: 1 → 0

*Barapasaurus tagorei*
Char. 171: 1 → 0
Char. 176: 0 → 1
Char. 194: 1 → 0
Char. 255: 2 → 1
Char. 274: 1 → 0
Char. 275: 3 → 0
Char. 279: 1 → 0
Char. 414: 0 → 1

*Tazoudasaurus naimi*
Char. 370: 0 → 1

*Gongxianosaurus shibeiensis*
No autapomorphies

*Amygdalodon patagonicus*
Char. 176: 0 → 1

*Patagosaurus fariasi*
Char. 142: 0 → 1
Char. 180: 0 → 1
Char. 258: 1 → 2
Char. 302: 1 → 0

*Cetiosaurus oxoniensis*
Char. 123: 0 → 1
Char. 130: 01 → 2
Char. 142: 0 → 1
Char. 176: 0 → 2
Char. 193: 0 → 1
Char. 221: 1 → 2
Char. 280: 0 → 1
Char. 341: 0 → 1
Char. 411: 0 → 1

*Shunosaurus lii*
Char. 83: 0 → 1
Char. 108: 0 → 1
Char. 113: 0 → 1
Char. 114: 0 → 2
Char. 144: 0 → 1
Char. 164: 0 → 1
Char. 175: 02 → 1
Char. 209: 1 → 0
Char. 267: 0 → 1
Char. 308: 1 → 0
Char. 328: 0 → 1
Char. 356: 0 → 1
Char. 361: 0 → 1
Char. 380: 0 → 1

*Omeisaurus* spp.
Char. 144: 0 → 1
Char. 193: 0 → 1
Char. 269: 1 → 0
Char. 287: 1 → 0
Char. 298: 1 → 2
Char. 308: 1 → 0
Char. 345: 0 → 1
Char. 357: 1 → 0
Char. 384: 0 → 1
Char. 413: 0 → 2
Char. 415: 0 → 1
Char. 428: 0 → 1
Char. 434: 0 → 1
Char. 435: 1 → 0
Char. 436: 1 → 0

*Mamenchisaurus* spp.
Char. 15: 1 → 0
Char. 60: 1 → 0
Char. 65: 1 → 0
Char. 134: 1 → 2
Char. 150: 0 → 1
Char. 194: 1 → 2
Char. 203: 0 → 1
Char. 222: 0 → 1
Char. 223: 0 → 2
Char. 229: 0 → 3
Char. 258: 1 → 2
Char. 280: 0 → 1
Char. 349: 0 → 1
Char. 419: 0 → 1

*Jobaria tiguidensis*
Char. 127: 0 → 1
Char. 141: 1 → 0
Char. 162: 1 → 0
Char. 205: 1 → 0
Char. 206: 2 → 1
Char. 314: 1 → 0

*Haplocanthosaurus priscus*
Char. 128: 0 → 1
Char. 141: 1 → 0
Char. 201: 1 → 0

*Brachytrachelopan mesai*
No autapomorphies

*Suuwassea emiliae*
Char. 1: 0 → 1
Char. 101: 1 → 2
Char. 105: 0 → 1
Char. 168: 1 → 2
Char. 229: 2 → 1
Char. 302: 1 → 2
Char. 450: 1 → 0

*Dicraeosaurus hansemanni*
Char. 144: 0 → 1

*Amargasaurus cazaui*
Char. 46: 0 → 2
Char. 76: 0 → 1
Char. 145: 0 → 1
Char. 152: 1 → 0
Char. 178: 0 → 2
Char. 298: 1 → 2
Char. 308: 1 → 0
Char. 331: 0 → 1
Char. 435: 1 → 2
Char. 439: 1 → 0

*Apatosaurus* spp.
Char. 77: 1 → 0
Char. 128: 0 → 1
Char. 283: 0 → 1
Char. 302: 1 → 2
Char. 308: 1 → 0
Char. 340: 0 → 1
Char. 354: 0 → 1
Char. 377: 0 → 1
Char. 383: 0 → 1
Char. 402: 1 → 0
Char. 417: 0 → 1
Char. 419: 0 → 1

*Barosaurus lentus*
Char. 217: 1 → 0
Char. 302: 1 → 0

*Diplodocus* spp.
Char. 156: 0 → 1
Char. 205: 0 → 1

*Histriasaurus boscarollii*
Char. 156: 0 → 1
Char. 416: 0 → 1

*Demandasaurus darwini*
Char. 101: 01 → 2
Char. 239: 0 → 1
Char. 248: 1 → 0

*Zapalasaurus bonapartei*
Char. 231: 0 → 1
Char. 235: 3 → 1
Char. 240: 0 → 1
Char. 244: 1 → 0

*Nigersaurus taqueti*
Char. 180: 0 → 1

*Comahuesaurus windhauseni*
Char. 168: 1 → 2
Char. 169: 1 → 2
Char. 196: 0 → 1
Char. 217: 1 → 0
Char. 417: 0 → 1

*Amazonsaurus maranhensis*
Char. 417: 0 → 1

*Lavocatisaurus agrioensis*
Char. 91: 1 → 0
Char. 126: 1 → 0

*Tataouinea hannibalis*
Char. 230: 0 → 1
Char. 231: 0 → 1
Char. 239: 0 → 1

*Cathartesaura anaerobica*
Char. 234: 0 → 1
Char. 254: 0 → 1

*Limaysaurus tessonei*
No autapomorphies

*Rebbachisaurus garasbae*
Char. 116: 1 → 0

*Katepensaurus goicoecheai*
No autapomorphies

*Turiasaurus riodevensis*
Char. 125: 1 → 0
Char. 136: 0 → 1
Char. 150: 0 → 1
Char. 156: 0 → 1
Char. 229: 0 → 2
Char. 259: 0 → 4
Char. 361: 1 → 0
Char. 362: 1 → 0
Char. 374: 1 → 0

*Losillasaurus giganteus*
Char. 125: 1 → 0
Char. 134: 1 → 0
Char. 169: 1 → 0
Char. 178: 0 → 1
Char. 187: 0 → 1
Char. 227: 0 → 1
Char. 229: 0 → 2
Char. 233: 0 → 1
Char. 244: 1 → 0
Char. 326: 1 → 0

*Bellusaurus sui*
Char. 43: 0 → 1
Char. 208: 0 → 1
Char. 222: 0 → 1
Char. 223: 0 → 2
Char. 229: 0 → 3
Char. 355: 0 → 1
Char. 428: 0 → 1
Char. 439: 1 → 0

*Camarasaurus* spp.
Char. 23: 1 → 0
Char. 59: 0 → 1
Char. 136: 0 → 1
Char. 150: 0 → 1
Char. 162: 1 → 0
Char. 194: 1 → 2
Char. 275: 3 → 1
Char. 298: 1 → 2
Char. 328: 0 → 1
Char. 425: 0 → 1
Char. 429: 0 → 2
Char. 445: 1 → 0
Char. 449: 0 → 1
Char. 452: 12 → 0

*Tehuelchesaurus benitezii*
Char. 157: 1 → 0
Char. 158: 1 → 0
Char. 162: 1 → 0
Char. 178: 0 → 1
Char. 180: 0 → 1
Char. 191: 1 → 0
Char. 194: 1 → 0

Char. 206: 2 → 1
Char. 289: 0 → 1
Char. 301: 1 → 0
Char. 308: 1 → 0
Char. 357: 1 → 0

*Galvesaurus herreroi*
Char. 142: 1 → 0
Char. 206: 2 → 1
Char. 207: 1 → 0
Char. 306: 1 → 0

*Europasaurus holgeri*
Char. 18: 1 → 0
Char. 25: 0 → 2
Char. 38: 1 → 0
Char. 45: 0 → 1
Char. 46: 0 → 1
Char. 79: 0 → 1
Char. 131: 0 → 1
Char. 135: 0 → 1
Char. 137: 0 → 1
Char. 138: 0 → 1
Char. 173: 1 → 2
Char. 193: 1 → 0
Char. 201: 1 → 0
Char. 202: 0 → 1
Char. 216: 1 → 0
Char. 235: 1 → 0
Char. 239: 1 → 0
Char. 278: 0 → 1
Char. 302: 1 → 0
Char. 328: 0 → 2
Char. 362: 1 → 0
Char. 413: 2 → 1

*Brachiosaurus altithorax*
Char. 37: 0 → 1
Char. 44: 0 → 1
Char. 168: 1 → 0
Char. 183: 1 → 0
Char. 304: 1 → 0
Char. 350: 0 → 1
Char. 360: 1 → 0

*Giraffatitan brancai*
Char. 27: 0 → 1
Char. 255: 01 → 2
Char. 275: 3 → 1
Char. 281: 1 → 0
Char. 389: 1 → 0

*Abydosaurus mcintoshi*
Char. 12: 1 → 0
Char. 62: 1 → 0
Char. 77: 1 → 0
Char. 92: 1 → 0
Char. 104: 0 → 1
Char. 140: 1 → 0

*Venenosaurus dicrocei*
Char. 412: 1 → 0

*Cedarosaurus weiskopfae*
Char. 239: 1 → 0
Char. 250: 0 → 1
Char. 258: 1 → 0
Char. 259: 0 → 1
Char. 286: 0 → 1
Char. 301: 1 → 0
Char. 356: 0 → 1

*Sauroposeidon proteles*
Char. 120: 0 → 1
Char. 164: 0 → 1
Char. 196: 0 → 1
Char. 239: 1 → 0
Char. 281: 1 → 0
Char. 356: 0 → 1

*Tastavinsaurus sanzi*
Char. 162: 1 → 0
Char. 200: 1 → 0
Char. 204: 0 → 1
Char. 254: 0 → 1
Char. 258: 1 → 0
Char. 259: 0 → 1
Char. 349: 0 → 1
Char. 384: 0 → 1
Char. 387: 1 → 0
Char. 396: 1 → 0

*Euhelopus zdanskyi*
Char. 120: 0 → 2
Char. 124: 2 → 3
Char. 127: 0 → 1
Char. 131: 0 → 1
Char. 176: 0 → 1
Char. 184: 0 → 1
Char. 192: 0 → 1
Char. 193: 1 → 0
Char. 196: 0 → 1
Char. 210: 0 → 1
Char. 212: 2 → 3
Char. 284: 1 → 0

*Phuwiangosaurus sirindhornae*
Char. 130: 2 → 1
Char. 328: 0 → 2

*Qiaowanlong kangxii*
Char. 335: 1 → 0

*Ruyangosaurus giganteus*
Char. 126: 1 → 0
Char. 132: 0 → 1
Char. 153: 2 → 1
Char. 169: 1 → 2
Char. 180: 0 → 1
Char. 181: 1 → 0
Char. 183: 1 → 0
Char. 229: 1 → 3
Char. 278: 0 → 1
Char. 279: 1 → 0
Char. 328: 0 → 1
Char. 348: 0 → 1
Char. 359: 0 → 1

*Erketu ellisoni*
No autapomorphies

*Chubutisaurus insignis*
Char. 162: 1 → 0
Char. 200: 1 → 0
Char. 345: 0 → 1
Char. 418: 0 → 1

*Ligabuesaurus leanzai*
Char. 151: 0 → 1

Char. 157: 1 → 0
Char. 161: 1 → 0
Char. 163: 2 → 1
Char. 173: 1 → 2
Char. 384: 0 → 1

*Wintonotitan wattsi*
Char. 169: 1 → 2
Char. 176: 1 → 0
Char. 259: 0 → 1

*Andesaurus delgadoi*
Char. 206: 2 → 0
Char. 231: 0 → 1
Char. 255: 1 → 2

*Malawisaurus dixeyi*
Char. 118: 3 → 2
Char. 171: 2 → 1
Char. 192: 1 → 0
Char. 225: 1 → 0
Char. 288: 1 → 0
Char. 308: 1 → 0
Char. 341: 0 → 1
Char. 363: 0 → 1
Char. 418: 0 → 2

*Paludititan nalatzensis*
Char. 176: 2 → 0
Char. 259: 1 → 0

*Tapuiasaurus macedoi*
Char. 122: 0 → 1
Char. 147: 1 → 2
Char. 149: 0 → 1
Char. 183: 1 → 0
Char. 193: 1 → 0
Char. 205: 2 → 1
Char. 207: 1 → 0
Char. 229: 3 → 1
Char. 296: 0 → 1
Char. 305: 2 → 0
Char. 369: 0 → 1
Char. 383: 1 → 2
Char. 409: 2 → 3
Char. 432: 2 → 0
Char. 435: 1 → 0

Char. 438: 0 → 1
Char. 442: 1 → 0

*Patagotitan mayorum*
Char. 176: 1 → 0
Char. 183: 1 → 0

*Argentinosaurus huinculensis*
Char. 160: 1 → 0
Char. 163: 2 → 1
Char. 424: 2 → 0

*Futalognkosaurus dukei*
Char. 234: 0 → 1
Char. 245: 0 → 1
Char. 350: 0 → 1

*Mendozasaurus neguyelap*
Char. 122: 0 → 1
Char. 132: 1 → 0
Char. 157: 3 → 0
Char. 421: 3 → 4
Char. 429: 2 → 1

*Austroposeidon magnificus*
Char. 124: 3 → 12
Char. 157: 1 → 0

BIBE 45854
Char. 137: 0 → 1

*Kaijutitan maui*
Char. 210: 1 → 0
Char. 362: 1 → 0

*Bonitasaura salgadoi*
Char. 258: 1 → 0

*Epachthosaurus sciuttoi*
Char. 169: 1 → 2
Char. 170: 0 → 1
Char. 183: 1 → 2
Char. 193: 1 → 2
Char. 196: 0 → 1
Char. 206: 2 → 1
Char. 215: 0 → 1
Char. 231: 0 → 2
Char. 239: 0 → 1

Char. 241: 0 → 2
Char. 249: 0 → 1
Char. 298: 2 → 3
Char. 369: 0 → 1
Char. 416: 0 → 1

*Notocolossus gonzalezparejasi*
Char. 160: 1 → 0

*Puertasaurus reuili*
No autapomorphies

*Muyelensaurus pecheni*
Char. 160: 1 → 0
Char. 183: 2 → 1
Char. 417: 0 → 1

*Maxakalisaurus topai*
Char. 155: 0 → 1
Char. 181: 1 → 0
Char. 208: 0 → 1
Char. 409: 1 → 3

*Adamantisaurus mezzalirai*
No autapomorphies

*Arrudatitan maximus*
Char. 259: 2 → 3
Char. 429: 2 → 1

*Rinconsaurus caudamirus*
Char. 255: 1 → 2

*Pitekunsaurus macayai*
Char. 130: 2 → 3
Char. 141: 0 → 1
Char. 142: 0 → 1
Char. 160: 1 → 0

*Brasilotitan nemophagus*
No autapomorphies

*Uberabatitan ribeiroi*
Char. 120: 1 → 2
Char. 148: 1 → 0

*Overosaurus paradasorum*
Char. 418: 2 → 1

*Trigonosaurus pricei*
Char. 234: 0 → 1

*Punatitan coughlini*
Char. 232: 0 → 1
Char. 255: 1 → 0

*Aeolosaurus rionegrinus*
No autapomorphies

*Gondwanatitan faustoi*
Char. 419: 1 → 0

*Dreadnoughtus schrani*
Char. 168: 2 → 1
Char. 170: 0 → 1
Char. 260: 0 → 1
Char. 308: 1 → 0
Char. 312: 1 → 0
Char. 328: 0 → 2
Char. 349: 0 → 1

*Diamantinasaurus matildae*
Char. 168: 2 → 1
Char. 183: 12 → 0
Char. 184: 0 → 1
Char. 187: 0 → 2
Char. 194: 0 → 1
Char. 273: 1 → 0
Char. 425: 0 → 1

*Mansourasaurus shahinae*
Char. 85: 0 → 1
Char. 303: 0 → 1
Char. 384: 0 → 1

*Lohuecotitan pandafilandi*
Char. 243: 0 → 1
Char. 250: 3 → 2
Char. 353: 2 → 1
Char. 367: 0 → 1

*Lirainosaurus astibiae*
Char. 167: 0 → 1
Char. 241: 2 → 1
Char. 349: 0 → 1
Char. 359: 0 → 1
Char. 361: 1 → 0

*Atsinganosaurus velauciensis*
Char. 115: 1 → 0
Char. 244: 1 → 0
Char. 258: 1 → 2
Char. 259: 1 → 3
Char. 361: 1 → 0

*Ampelosaurus atacis*
Char. 187: 0 → 12
Char. 257: 0 → 1

*Rapetosaurus krausei*
Char. 142: 0 → 1
Char. 252: 0 → 1
Char. 258: 1 → 2
Char. 310: 1 → 0
Char. 330: 1 → 0
Char. 418: 0 → 1
Char. 423: 0 → 2
Char. 425: 0 → 1

*Isisaurus colberti*
Char. 137: 0 → 1
Char. 183: 2 → 0
Char. 193: 2 → 0
Char. 207: 1 → 0
Char. 241: 2 → 0
Char. 272: 1 → 0
Char. 274: 0 → 1
Char. 275: 0 → 3
Char. 302: 2 → 1
Char. 327: 1 → 0
Char. 345: 0 → 1
Char. 421: 0 → 3

*Nemegtosaurus mongoliensis*
Char. 159: 3 → 2

*Opisthocoelicaudia skarzynskii*
Char. 427: 1 → 0

*Alamosaurus sanjuanensis*
Char. 217: 0 → 1
Char. 224: 0 → 1
Char. 231: 1 → 0

*Baurutitan britoi*
Char. 234: 0 → 1
Char. 416: 0 → 1

*Bonatitan reigi*
Char. 124: 3 → 1
Char. 126: 1 → 0
Char. 358: 1 → 0

*Bravasaurus arrierosorum*
Char. 147: 1 → 2
Char. 173: 0 → 1
Char. 187: 0 → 2

*Ibirania parva*
Char. 160: 0 → 1
Char. 161: 0 → 1
Char. 189: 0 → 1
Char. 190: 1 → 0
Char. 191: 1 → 0
Char. 201: 1 → 0
Char. 203: 0 → 1
Char. 206: 2 → 0
Char. 231: 1 → 0
Char. 426: 0 → 1

*Rocasaurus muniozi*
Char. 416: 0 → 1

MACN PV RN 233
No autapomorphies

*Neuquensaurus australis*
Char. 206: 2 → 1
Char. 369: 1 → 0
Char. 440: 2 → 1

*Saltasaurus loricatus*
No autapomorphies

Vila Ventura axis
Char. 437: 0 → 1
Char. 438: 0 → 2
Char. 447: 0 → 1
Char. 448: 0 → 1

Node 108 - Sauropodiformes
Char. 84: 0 → 1

Node 109
No synapomorphies

Node 110
Char. 198: 0 → 1
Char. 206: 0 → 1
Char. 309: 0 → 1

Node 111 - Sauropoda
Char. 161: 0 → 1
Char. 199: 0 → 1
Char. 274: 0 → 1
Char. 275: 0 → 1
Char. 307: 0 → 1
Char. 385: 0 → 1

Node 112 - Vulcanodontidae
Char. 400: 0 → 1

Node 113 - Gravisauria
Char. 139: 0 → 1
Char. 164: 1 → 0
Char. 206: 1 → 2
Char. 301: 0 → 1
Char. 311: 0 → 1
Char. 315: 0 → 1
Char. 378: 0 → 1
Char. 391: 0 → 1
Char. 397: 0 → 1

Node 114
Char. 298: 0 → 1
Char. 351: 0 → 1
Char. 395: 0 → 1

Node 115
Char. 178: 0 → 1
Char. 195: 0 → 1
Char. 413: 1 → 0

Node 116
Char. 0: 0 → 1
Char. 66: 0 → 1
Char. 272: 0 → 1

Node 117 - Eusauropoda
Char. 171: 0 → 1
Char. 194: 0 → 1
Char. 275: 1 → 3
Char. 279: 0 → 1
Char. 360: 1 → 0

Node 118
Char. 124: 0 → 1
Char. 166: 0 → 1
Char. 357: 0 → 1

Node 119
Char. 114: 0 → 2

Node 120
Char. 114: 0 → 1
Char. 122: 0 → 1
Char. 123: 0 → 1
Char. 134: 0 → 1
Char. 154: 0 → 1
Char. 205: 0 → 1
Char. 380: 0 → 1

Node 121
Char. 14: 0 → 1
Char. 157: 0 → 1
Char. 158: 0 → 1
Char. 168: 0 → 1
Char. 169: 0 → 1
Char. 314: 0 → 1
Char. 317: 0 → 1
Char. 361: 0 → 1
Char. 374: 0 → 1
Char. 439: 0 → 1
Char. 445: 0 → 1

Node 122
Char. 114: 12 → 0
Char. 128: 0 → 1
Char. 134: 1 → 0
Char. 144: 0 → 1

Node 123 - Diplodocimorpha
Char. 151: 0 → 1
Char. 155: 0 → 1
Char. 172: 0 → 1
Char. 173: 1 → 2
Char. 277: 0 → 1
Char. 328: 0 → 1

Node 124 - Neosauropoda
Char. 20: 0 → 1
Char. 21: 1 → 0
Char. 91: 0 → 1
Char. 130: 0 → 1
Char. 142: 0 → 1
Char. 152: 0 → 1
Char. 178: 1 → 0
Char. 217: 0 → 1
Char. 244: 0 → 1
Char. 316: 0 → 1
Char. 343: 0 → 1
Char. 371: 0 → 1
Char. 409: 0 → 2

Node 125
Char. 122: 1 → 0
Char. 156: 0 → 1
Char. 202: 0 → 1

Node 126
Char. 85: 0 → 1
Char. 208: 0 → 1

Node 127 - Dicraeosaurinae
Char. 202: 0 → 1

Node 128
Char. 37: 0 → 1
Char. 49: 0 → 1
Char. 123: 1 → 0
Char. 125: 12 → 3
Char. 154: 1 → 0
Char. 159: 1 → 0
Char. 449: 0 → 1
Char. 450: 1 → 2

Node 129 - Dicraeosauridae
Char. 3: 0 → 1
Char. 28: 0 → 1
Char. 36: 1 → 0
Char. 45: 0 → 1
Char. 52: 0 → 1
Char. 88: 0 → 1
Char. 107: 3 → 2
Char. 170: 0 → 2

Node 130 - Flagellicaudata
- Char. 13: 1 → 2
- Char. 150: 0 → 1
- Char. 189: 0 → 1
- Char. 229: 0 → 2
- Char. 234: 0 → 1
- Char. 258: 1 → 2
- Char. 274: 1 → 0
- Char. 275: 2 → 0
- Char. 277: 12 → 0
- Char. 333: 0 → 1
- Char. 342: 1 → 0
- Char. 343: 1 → 0
- Char. 344: 0 → 1
- Char. 384: 0 → 1
- Char. 428: 0 → 1

Node 131 - Diplodocoidea
- Char. 98: 1 → 2
- Char. 99: 1 → 3
- Char. 105: 1 → 0
- Char. 106: 1 → 0
- Char. 107: 1 → 3
- Char. 108: 1 → 3
- Char. 145: 1 → 0
- Char. 162: 1 → 0
- Char. 165: 1 → 2
- Char. 194: 1 → 0
- Char. 205: 1 → 0
- Char. 215: 0 → 1
- Char. 235: 0 → 3
- Char. 241: 0 → 1
- Char. 257: 0 → 1

Node 132 - Diplodocidae
- Char. 27: 0 → 1
- Char. 29: 0 → 1
- Char. 30: 0 → 1
- Char. 46: 0 → 1
- Char. 109: 1 → 0
- Char. 134: 1 → 2
- Char. 141: 1 → 2
- Char. 153: 2 → 4
- Char. 155: 1 → 0
- Char. 180: 0 → 1
- Char. 237: 0 → 1
- Char. 238: 0 → 1
- Char. 243: 0 → 1

Node 133 - Diplodocinae
- Char. 140: 0 → 1
- Char. 143: 0 → 1
- Char. 151: 1 → 0
- Char. 230: 0 → 1
- Char. 231: 0 → 1
- Char. 232: 0 → 1
- Char. 240: 0 → 1
- Char. 248: 0 → 2
- Char. 249: 0 → 1
- Char. 255: 2 → 3

Node 134
- Char. 197: 0 → 1
- Char. 228: 0 → 1

Node 135 - Rebbachisauridae
- Char. 239: 1 → 0
- Char. 248: 0 → 1
- Char. 411: 1 → 0

Node 136 - Nigersaurini
- Char. 178: 2 → 1
- Char. 189: 0 → 1

Node 137
- Char. 410: 0 → 1

Node 138 - Rebbachisaurinae
- Char. 233: 0 → 1
- Char. 249: 0 → 1
- Char. 338: 0 → 1
- Char. 407: 0 → 1

Node 139 - Khebbashia
- Char. 112: 0 → 1
- Char. 117: 0 → 1
- Char. 302: 1 → 0

Node 140
- Char. 411: 0 → 1

Node 141
- Char. 178: 0 → 1
- Char. 229: 0 → 4
- Char. 336: 0 → 1

Node 142
- Char. 234: 0 → 1

Node 143 - Limaysaurinae
- Char. 243: 0 → 1
- Char. 282: 0 → 1

Node 144 - Macronaria
- Char. 122: 1 → 0
- Char. 130: 1 → 2
- Char. 163: 0 → 1
- Char. 193: 0 → 1
- Char. 235: 0 → 1
- Char. 281: 0 → 1
- Char. 335: 0 → 1
- Char. 340: 0 → 1
- Char. 414: 0 → 1

Node 145 - Camarasauromorpha
- Char. 413: 0 → 2

Node 146
- Char. 171: 1 → 0
- Char. 207: 0 → 1
- Char. 211: 0 → 1
- Char. 354: 0 → 1
- Char. 414: 1 → 0

Node 147
- Char. 44: 1 → 0
- Char. 75: 0 → 1
- Char. 76: 0 → 1
- Char. 106: 1 → 0
- Char. 227: 0 → 1
- Char. 251: 0 → 1
- Char. 306: 0 → 1
- Char. 320: 0 → 1
- Char. 321: 0 → 1

Node 148
- Char. 130: 2 → 0
- Char. 133: 0 → 1

Node 149 - Somphospondyli
- Char. 167: 0 → 1
- Char. 280: 0 → 1

Node 150 - Brachiosauridae
- Char. 1: 1 → 2
- Char. 4: 0 → 1
- Char. 5: 0 → 1
- Char. 89: 0 → 1
- Char. 100: 0 → 1
- Char. 138: 0 → 1
- Char. 163: 12 → 0
- Char. 167: 1 → 0
- Char. 169: 1 → 0
- Char. 179: 0 → 1
- Char. 194: 1 → 2
- Char. 201: 1 → 0
- Char. 203: 0 → 1
- Char. 226: 0 → 1
- Char. 298: 2 → 3
- Char. 390: 0 → 1

Node 151 - Titanosauriformes
- Char. 171: 0 → 2
- Char. 172: 0 → 1
- Char. 235: 1 → 0
- Char. 298: 1 → 2

Node 152
- Char. 183: 0 → 1
- Char. 192: 0 → 1
- Char. 205: 2 → 1
- Char. 210: 0 → 1
- Char. 353: 1 → 2

Node 153
- Char. 332: 0 → 1
- Char. 389: 0 → 1

Node 154
- Char. 124: 3 → 2
- Char. 127: 0 → 1
- Char. 144: 0 → 1

Node 155
- Char. 120: 0 → 2
- Char. 363: 0 → 1

Node 156
- Char. 156: 0 → 1
- Char. 178: 0 → 1
- Char. 205: 1 → 2

Node 157
- Char. 56: 0 → 1
- Char. 67: 0 → 1
- Char. 107: 1 → 2
- Char. 111: 0 → 1
- Char. 122: 1 → 0
- Char. 124: 2 → 3
- Char. 291: 0 → 1
- Char. 292: 0 → 1
- Char. 312: 0 → 1

Node 158
- Char. 273: 0 → 1
- Char. 356: 0 → 1

Node 159 - Titanosauria
- Char. 229: 1 → 2
- Char. 268: 0 → 1
- Char. 304: 1 → 0

Node 160
- Char. 164: 0 → 1
- Char. 232: 0 → 1
- Char. 346: 0 → 1

Node 161 - Lithostrotia
- Char. 120: 0 → 1
- Char. 146: 0 → 1
- Char. 220: 0 → 1
- Char. 222: 0 → 1
- Char. 258: 1 → 0
- Char. 302: 0 → 1
- Char. 339: 0 → 1

Node 162
- Char. 346: 1 → 0

Node 163
- Char. 241: 2 → 0
- Char. 351: 1 → 2

Node 164 - Lirainosaurinae
- Char. 280: 1 → 0
- Char. 289: 1 → 0
- Char. 305: 0 → 1

Node 165
- Char. 300: 0 → 1

Node 166 - Diamantinasauria
- Char. 124: 3 → 2
- Char. 167: 1 → 0
- Char. 172: 0 → 1
- Char. 176: 1 → 2
- Char. 283: 1 → 0
- Char. 305: 2 → 0
- Char. 357: 1 → 0

Node 167 - Saltasauroidea
- Char. 107: 2 → 3
- Char. 204: 0 → 1
- Char. 221: 1 → 2
- Char. 246: 1 → 0
- Char. 279: 1 → 0
- Char. 295: 0 → 1

Node 168 - Lohuecosauria
- Char. 148: 1 → 0
- Char. 194: 1 → 0
- Char. 225: 1 → 0
- Char. 241: 0 → 2
- Char. 274: 1 → 0
- Char. 275: 3 → 0
- Char. 288: 1 → 0
- Char. 302: 1 → 2
- Char. 310: 0 → 1
- Char. 412: 1 → 0
- Char. 421: 3 → 0

Node 169
- Char. 161: 1 → 0
- Char. 171: 2 → 0
- Char. 172: 1 → 0
- Char. 175: 0 → 2
- Char. 177: 0 → 1
- Char. 178: 1 → 2
- Char. 258: 0 → 1
- Char. 313: 0 → 1

Node 170
- Char. 132: 0 → 1
- Char. 178: 0 → 1
- Char. 246: 0 → 1

Char. 255: 1 → 2
Char. 304: 0 → 1

Node 171 - Eutitanosauria
Char. 126: 1 → 0
Char. 149: 1 → 0
Char. 229: 2 → 3
Char. 232: 1 → 0
Char. 259: 0 → 2
Char. 305: 1 → 2
Char. 421: 2 → 3

Node 172
Char. 118: 3 → 4
Char. 168: 2 → 1
Char. 250: 3 → 2
Char. 443: 0 → 1

Node 173 - Colossosauria
Char. 151: 0 → 1
Char. 153: 3 → 4
Char. 157: 1 → 3
Char. 176: 1 → 0
Char. 453: 0 → 1

Node 174
Char. 173: 1 → 2
Char. 194: 1 → 2

Node 175 - Lognkosauria
Char. 148: 1 → 2
Char. 444: 0 → 1

Node 176
Char. 122: 0 → 1
Char. 149: 0 → 1
Char. 224: 0 → 1
Char. 235: 1 → 2
Char. 255: 1 → 2
Char. 373: 1 → 0
Char. 419: 0 → 1

Node 177
Char. 98: 1 → 2
Char. 126: 0 → 1
Char. 128: 0 → 1
Char. 133: 0 → 1
Char. 142: 0 → 1
Char. 174: 2 → 03
Char. 252: 0 → 1
Char. 288: 1 → 0
Char. 350: 0 → 1

Node 178
Char. 231: 0 → 2
Char. 241: 0 → 2
Char. 421: 3 → 1
Char. 430: 0 → 1

Node 179
Char. 255: 2 → 1
Char. 424: 2 → 0

Node 180
Char. 169: 12 → 0
Char. 422: 1 → 2
Char. 423: 1 → 2

Node 181
Char. 259: 3 → 2
Char. 416: 1 → 0

Node 182
Char. 194: 1 → 0
Char. 231: 2 → 1

Node 183 - Rinconsauria
Char. 183: 1 → 2
Char. 220: 1 → 0
Char. 260: 0 → 1
Char. 286: 0 → 1
Char. 418: 0 → 1

Node 184
Char. 228: 0 → 1
Char. 416: 0 → 1
Char. 431: 1 → 0

Node 185
Char. 253: 0 → 1
Char. 411: 1 → 0
Char. 419: 0 → 1

Node 186
Char. 252: 0 → 1
Char. 418: 1 → 2

Node 187
Char. 277: 0 → 1
Char. 429: 2 → 1

Node 188
Char. 130: 12 → 3
Char. 139: 1 → 2

Node 189
Char. 142: 0 → 1
Char. 158: 1 → 3
Char. 160: 1 → 0
Char. 232: 0 → 1

Node 190
Char. 128: 0 → 1
Char. 139: 1 → 2
Char. 180: 0 → 1
Char. 193: 1 → 2
Char. 196: 0 → 1

Node 191
Char. 249: 0 → 1
Char. 252: 1 → 2
Char. 429: 2 → 0

Node 192 - Aeolosaurini
Char. 251: 1 → 2
Char. 430: 1 → 0

Node 193
Char. 248: 0 → 1
Char. 260: 0 → 1

Node 194
Char. 158: 1 → 3
Char. 285: 1 → 0
Char. 289: 1 → 0

Node 195
Char. 126: 0 → 1
Char. 145: 1 → 0
Char. 149: 0 → 1
Char. 160: 1 → 0
Char. 193: 1 → 2
Char. 232: 0 → 1

Char. 249: 0 → 1
Char. 303: 0 → 1

Node 196
Char. 176: 1 → 2
Char. 184: 0 → 1
Char. 207: 1 → 0
Char. 229: 3 → 5
Char. 428: 2 → 0

Node 197 - Saltasauridae
Char. 67: 1 → 0
Char. 72: 0 → 1
Char. 196: 0 → 1
Char. 258: 1 → 0
Char. 288: 0 → 1
Char. 300: 0 → 1
Char. 304: 1 → 0
Char. 349: 0 → 1

Node 198
Char. 255: 2 → 1
Char. 418: 0 → 1
Char. 429: 0 → 1

Node 199 - Saltasaurinae
Char. 194: 0 → 1
Char. 256: 0 → 1
Char. 257: 0 → 1
Char. 328: 0 → 1
Char. 347: 0 → 1
Char. 368: 1 → 0

Node 200
Char. 427: 1 → 0

Node 201
Char. 131: 0 → 1
Char. 142: 0 → 1
Char. 253: 0 → 1

Node 202
Char. 219: 0 → 1
Char. 260: 0 → 1
Char. 351: 1 → 2
Char. 417: 0 → 1
Char. 425: 0 → 12

Node 203
Char. 249: 1 → 2

Node 204 - Saltasaurini
Char. 156: 0 → 1
Char. 296: 0 → 1
Char. 416: 0 → 1
